# Data‐Driven Design and Fabrication of Heat‐Resistant, Ultrastrong, Lightweight Aluminum‐Based Entropy Alloy by Additive Manufacturing

**DOI:** 10.1002/advs.202522817

**Published:** 2026-01-21

**Authors:** Enmao Wang, Chao Ding, Danyang Zhou, Chenjin Xu, Swee Leong Sing, Jianzhong Jiang, Huibin Wu

**Affiliations:** ^1^ Collaborative Innovation Center of Steel Technology University of Science and Technology Beijing Beijing 100083 China; ^2^ Department of Mechanical Engineering National University of Singapore Singapore 117575 Singapore; ^3^ School of Materials Science and Engineering Fuyao University of Science and Technology Fuzhou Fujian 350109 China

**Keywords:** additive manufacturing, data‐driven design, heat‐resistant, hierarchical nanostructures, lightweight aluminum‐based entropy alloys

## Abstract

Additive manufacturing (AM) of heat‐resistant high‐strength aluminum (Al) alloys for load‐bearing components faces a fundamental dichotomy: traditional high‐strength compositions suffer from hot cracking, while printable alloys lack sufficient high‐temperature strength. This inherent conflict severely restricts the design space for novel alloys in demanding applications like aerospace. Addressing this challenge, a data‐driven design strategy leveraging quantum machine learning (QML) and high‐throughput computing identifies an ultrastrong Al_85_Cu_5_Li_4_Mg_3_Zn_3_ lightweight Al‐based entropy alloy (LAEA) tailored for AM. The AM process transforms potentially brittle microsized intermetallic compounds into deformable hierarchical nanostructures of cellular eutectics, quasicrystals, and dense nanosized planar defects (stacking faults, nanotwin boundaries, and 9R phases). This intricate microstructure endows the as‐printed alloy with exceptional properties: an ultrastrong compressive strength exceeding 1000 MPa coupled with considerable plasticity (∼20%), outstanding high‐temperature strength (>800 MPa at 200°C), and a specific strength (350 × 10^3^ N m/kg) rivaling titanium alloys. Furthermore, a controllable quasicrystal‐to‐crystal phase transformation activated by thermal exposure offers an additional mechanism for precisely tuning mechanical properties post‐fabrication. This work presents a novel design paradigm for AM‐compatible high‐performance lightweight Al‐based entropy alloys (LAEAs), effectively bridging advanced computational material design and advanced manufacturing.

## Graphical abstract

A data‐driven strategy integrating quantum machine learning (QML) and high‐throughput computing overcomes hot‐cracking limitation to design a novel lightweight aluminum‐based entropy alloy for additive manufacturing. The fabrication transforms brittle intermetallics into deformable hierarchical nanostructures, yielding ultrastrong strength (>1 GPa) and exceptional high‐temperature strength (>800 MPa at 200°C). A controllable quasicrystal‐to‐crystal phase transformation offers an additional mechanism for property tuning.

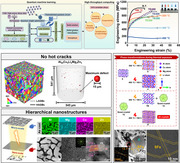

## 1 Introduction

The increasing demand for heat‐resistant, high‐strength, lightweight materials in advanced manufacturing sectors such as aerospace, automotive, and rail transportation has made Al alloys essential structural materials due to their excellent high ratio of strength to weight and corrosion resistance. AM is highly attractive for fabricating load‐bearing components with complex geometries from these alloys due to its ability for precise control [1, 2, 3]. However, traditional high‐strength Al alloys, such as Al‐Cu‐Mg and Al‐Zn‐Mg‐Cu systems, in AM face significant challenges [4]. The rapid cooling rates and complex thermal gradients in AM cause thermal stress accumulation and a broad solidification range, leading to hot cracking and instability in both microstructure and mechanical properties. Conversely, compositions that exhibit good printability, such as near‐eutectic Al‐Si alloys or modified systems, suffer from rapid strength degradation at elevated temperatures [5]. Therefore, this dichotomy between printability and heat resistance severely limits the development of novel high‐performance Al alloys for extreme thermal environments. To address these issues, most current research focuses on modifying existing high‐strength Al alloy systems via in situ micro‐alloying/non‐in situ ceramic particles to induce the precipitation of nanosized Al_3_X (i.e., L1_2_/D0_22_/D0_23_ ordered phases, e.g., Al_3_Sc, Al_3_Zr, Al_3_Ti, Al_3_Nb), thereby promoting extensive heterogeneous nucleation during rapid solidification, refining grains, facilitating equiaxed grain growth, improving mechanical properties, and mitigating hot cracking [6, 7, 8, 9, 10, 11]. Although impressive outcomes have been achieved in hot‐crack inhibition and property enhancement, the following issues remain: (1) elevated costs associated with rare earth elements; (2) fluctuations in densification and mechanical properties caused by variability in shape, size, and distribution of ceramic particles; (3) an inherent inability to fundamentally expand the compositional space for designing novel heat‐resistant and printable alloys. Breaking this trade‐off requires novel in situ alloy design strategies.

High‐entropy alloys (HEAs) have become a prominent focus in materials science due to their unique multicomponent design and excellent extreme properties [12, 13]. Initially, HEAs were designed with equimolar compositions and high configurational entropy to form a stable solid solution, exemplified by CrCoFeMnNi [14, 15]. Recently, HEAs evolved to incorporate nonequimolar and trace elements, with less emphasis on strict entropy values or single‐phase solid solution. Entropy modulation rather than solely on entropy maximization is focused, leading to the emergence of various novel HEAs, including eutectic high‐entropy alloys (EHEAs) [16], high‐entropy intermetallic compounds (HEIC) [17], and lightweight Al‐based entropy alloys (LAEAs) [18, 19]. LAEAs take advantage of aluminum's low density and high‐entropy effects to achieve enhanced mechanical properties at both room and elevated temperatures through element synergy, gradually becoming a highly promising category in lightweight alloys. LAEAs are mainly fabricated through casting and thermomechanical processing, and the significant differences in electronegativity and atomic radius among the constituent elements often lead to the inevitable formation of microsized intermetallic compounds (ICs), resulting in a severe compressive strength‐ductility trade‐off [20, 21, 22, 23, 24]. Therefore, Wu et al. [25] attempted to reduce the differences in atomic size and electronegativity between solute elements and Al by applying high pressure and high temperature (HPHT). Synergizing with high entropy effects, they successfully facilitated the transition from ICs to solid solutions, thus preparing a small‐sized lightweight single‐phase Al‐based complex concentrated alloy (Al_55_Mg_35_Li_5_Zn_5_) with high specific strength. This alloy exhibits a compressive strength of up to ∼900 MPa, but its compressive plasticity is only ∼4%. This indicates that a forced solid solution structure has not effectively resolved the strength‐ductility trade‐off. In addition, the application of LAEAs with complex components in AM remains unexplored.

The compositional design of LAEAs for AM applications is highly complex. Traditional alloy design concepts reliant on CALPHAD are often inefficient, costly, and insufficient for rapidly exploring vast compositional spaces [26]. Advances in machine learning (ML) and high‐throughput computing (HTC) have enabled data‐driven design (DDD) to significantly accelerate the materials development process [27, 28, 29]. Integrating DDD with AM enables efficient compositional design and precise manufacturing of high‐performance LAEAs. Quantum machine learning (QML), an emerging fusion of quantum computing and machine learning, provides transformative solutions for material design. QML excels in parallel computing within high‐dimensional data spaces, efficiently analyzing complex multicomponent alloy systems and capturing nonlinear relationships between material properties and microstructure [30, 31]. Specifically, QML outperforms traditional ML in predictive accuracy and generalization when applied to phase structure prediction of HEAs [32]. Furthermore, QML efficiently processes high‐dimensional quantum features by utilizing quantum superposition and quantum entanglement, enabling rapid compression, extraction, and optimization of extensive material datasets.

Focusing on the compositional frameworks of traditional high‐strength Al‐Cu and Al‐Zn‐Mg‐Cu alloys, incorporating Li to reduce density, in which the atomic fraction of Al is maintained between 80% and 90%, with other elements contributing at least 2% each, ensuring a sufficient proportion of solid solution and distinguishing it from the low‐alloyed aluminium alloys, we apply DDD (QML combined with HTC) to systematically screen and optimize all possible compositions. We successfully find a novel Al_85_Cu_5_Li_4_Mg_3_Zn_3_ LAEA and fabricate an alloy via selective laser melting (SLM), transforming brittle, microsized ICs into deformable nanosized cellular eutectic phases, quasicrystalline strengthening phases, and noncoherent precipitates. This transformation induces a heterogeneous structure and heterogeneous deformation‐induced (HDI) stress of more than 400 MPa. The precisely constructed composition unexpectedly introduces nanosized planar defects through SLM, including stacking faults (SFs), nanotwin boundaries, and 9R phases. The as‐printed alloys exhibit ultrastrong compressive strength exceeding 1000 MPa and nearly 20% compressive plasticity. Its specific strength (350 × 10^3^ N m/kg) surpasses that of most titanium alloys. The high volume fraction of Cu‐containing phases with low diffusion coefficients and stable nanosized planar defects enhances high‐temperature properties, particularly maintaining 95% yield strength (507 MPa) retention ratio and ∼800 MPa compressive strength at 200°C. In situ micropillar compression at 200°C reveals high‐temperature HDI stress exceeding 300 MPa in microzones. Additionally, the phase transformation from the quasicrystalline phase (T2‐Al_6_CuLi_3_) to the body‐centered cubic phase (R‐Al_5_CuLi_3_) which occurs at 400°C, activates inherent SFs within the nanosized R phase during room‐temperature deformation, synergistically enhancing compressive strength (921 MPa) and compressive plasticity (34%). This work provides an innovative design strategy and a practical pathway for the precise customization of high‐performance LAEAs using AM.

## 2 Results

### 2.1 Data‐Driven Design of Alloy Composition

**FIGURE 1:**
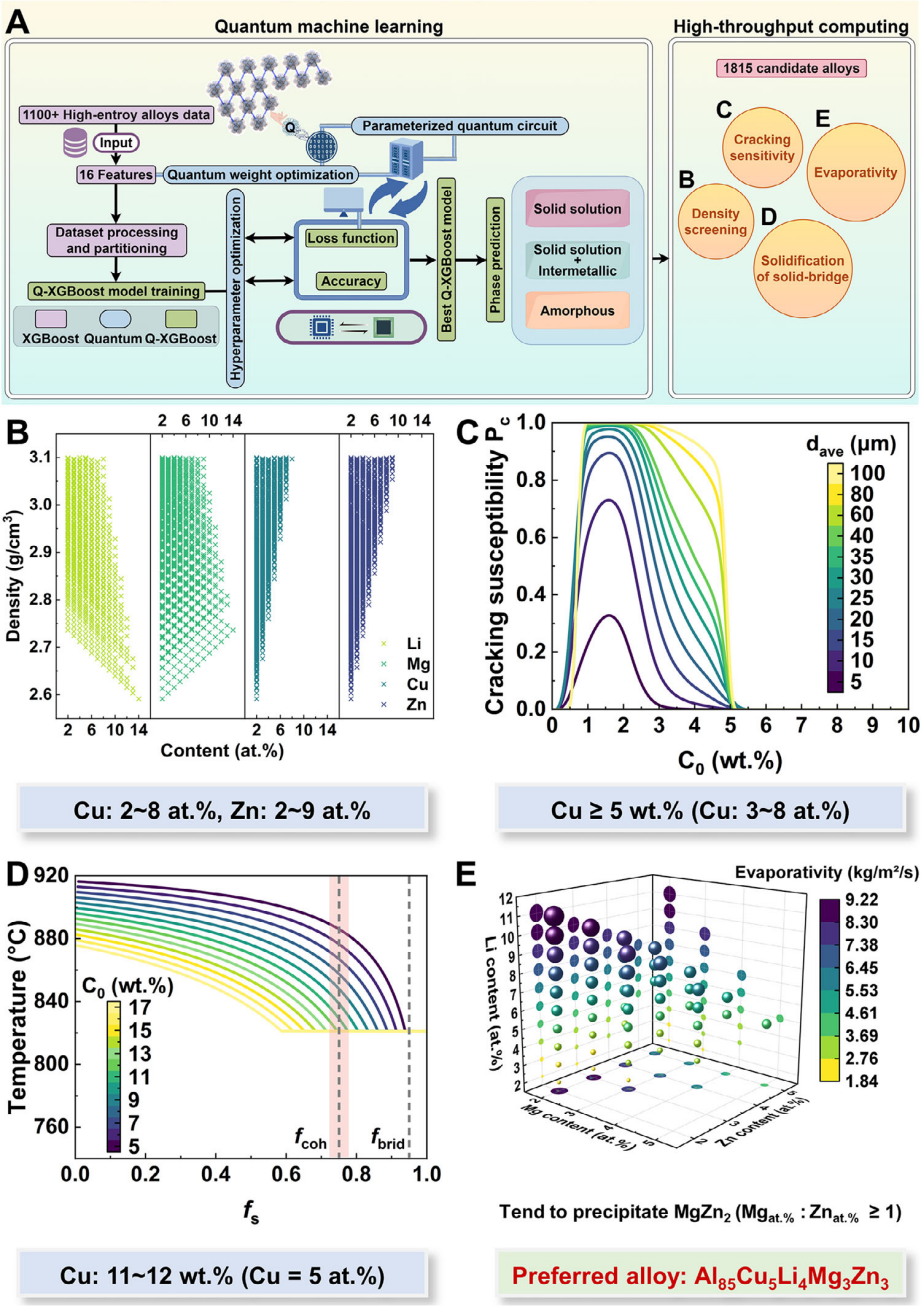
Data‐driven design strategy for as‐printed Al_85_Cu_5_Li_4_Mg_3_Zn_3_ lightweight Al‐based entropy alloy (LAEA). A) Schematic view of quantum machine learning (QML) and high‐throughput computing (HTC) processes. B) Distribution of elemental content after density screening. C) Hot cracking susceptibility at different Cu contents and grain diameters under rapid cooling of selective laser melting (SLM). D) Solidification curves at different Cu contents under rapid cooling of SLM. E) 3D visualization showing the theoretical evaporation rates of low melting point elements (Li, Mg, and Zn) in the SLM process.

Herein, we examine 1,815 candidate alloys composed of five elements—Al, Li, Mg, Cu, and Zn—that meet specific atomic ratio requirements. Since LAEAs are more prone to developing ordered phases than traditional HEAs, phase stability directly influences the mechanical properties of printed alloys. Thus, we require a broader set of physical characteristics to accurately identify the phase structures of candidate alloys and assess the likelihood of single solid solution formation [33]. Figure 1A illustrates the QML process using a quantum‐XGBoost hybrid model for training and ternary classification of phase structures (Solid solution, solid solution + intermetallic, amorphous). As a gradient boosting ensemble model, the validity of XGBoost as a classic baseline has been verified by numerous studies on phase prediction of high‐entropy alloys due to its outstanding performance and interpretability in classification tasks [34, 35, 36, 37]. Although deep learning has great potential, its predictive advantages are not significant under the current scale of high‐entropy alloy data, especially since data augmentation can easily lead to high variance and overfitting [32, 38, 39, 40]. XGBoost enables simple and efficient training while directly providing a feature importance ranking based on physical descriptors, making it well‐suited for rapid iterative compositional design. However, traditional XGBoost relies on gain or split‐tree structures to assess feature importance, which may lead to misjudgments in high‐dimensional datasets [34]. By using quantum optimization algorithms, the quantum‐XGBoost hybrid model efficiently and accurately evaluates each feature's contribution to model predictions, enabling it to detect previously overlooked but crucial features. Without feature dimensionality reduction, quantum computing can rapidly explore the multidimensional space to examine various feature combinations, leveraging quantum superposition and entanglement to more effectively uncover nonlinear higher order interactions among features (Figure S1, Supporting Information). The quantum‐XGBoost hybrid model's acute capture of subtle boundaries reduces misclassification in solid solution and solid solution + intermetallic (Figure S2, Supporting Information). The receiver operating characteristic (ROC) curves further reveal that the quantum‐XGBoost hybrid model achieves higher area under the curve (AUC) values for all classes compared to traditional models, indicating enhanced discriminative power. We also examined the loss and accuracy curves during training and validation, noting that the quantum‐XGBoost hybrid model converges more rapidly, exhibits lower validation loss, and demonstrates superior generalization performance. Notably, three‐dimensional performance visualizations demonstrate the quantum‐XGBoost hybrid model's comprehensive superiority in precision, recall, and F1‐score, achieving near‐perfect recognition in the solid solution/solid solution + intermetallic class. All candidate alloys are classified as solid solution + intermetallic by the quantum‐XGBoost hybrid model. To ensure the reliability of the identify, we have also performed 50 iterations of Bootstrap resampling to quantify the cognitive uncertainty of the two machine learning models. It should be emphasized that although both the traditional XGBoost and Q‐XGBoost models achieve high classification accuracy on the validation set, their underlying predictive behaviors exhibit fundamental differences. As shown in Figure S3 (Supporting Information), while the traditional XGBoost model correctly classifies candidates, it operates with marginal confidence, where predicted probabilities fall within the range of 0.55 to 0.81. This indicates that decisions are made near the classification boundary, rendering the model susceptible to data noise and diminishing the reliability of its potential generalization. However, our Q‐XGBoost model effectively disentangles complex feature correlations using quantum‐weighted features, pushing predictions away from the decision boundary and shifting the probability distribution toward the high‐confidence region between 0.83 and 1.0. This confirms that the QML model not only delivers accurate predictions but also enhances robustness, thereby providing a greater margin of safety for the experiments.

The SHapley Additive exPlanations (SHAP) analysis reveals the contributions and nonlinear interactions of key features in different classes, further confirming the superior modeling capability of quantum computing in complex high‐dimensional feature spaces, as shown in Figure S4 (Supporting Information). In the solid solution + intermetallic prediction, mean_VEC shows a pronounced positive contribution in the low‐value interval, closely linked to the strong driving forces of lattice distortions and electron localization, which readily induce the formation of intermetallic phases [35]. In contrast, despite ΔSmix showing limited overall importance, its nonlinear trend still exerts a positive influence on the solid solution + intermetallic prediction, particularly when acting synergistically with low mean_VEC. An increase in entropy alone does not significantly influence the stability of solid solution + intermetallic. Therefore, the predicted results for the candidate alloys suggest that the printability and mechanical properties of LAEAs without altering the phase class can be optimized by adjusting the entropy.

To meet the property requirements of AM, a subsequent multidimensional compositional optimization is performed using HTC, as shown in Figure 1A. Density screening is the initial step, with a theoretical density threshold set at less than 3.1 g/cm^3^. Since elements with low melting points (e.g., Li, Mg, Zn) tend to evaporate during AM, the actual density typically decreases. Therefore, this threshold ensures a density close to that of traditional Al alloys (∼3.0 g/cm^3^ or lower) after printing. Initially, Cu and Zn contents are limited to 2–8 at.% and 2–9 at.%, respectively (Figure 1B). Under AM thermal conditions, hot cracking susceptibility is evaluated by coupling multiple variables, with particular attention to Cu due to its notable tendency for hot cracking. The calculations show that hot cracking susceptibility depends on both Cu content and grain diameter (Figure 1C). However, once Cu content exceeds 5 wt.% (3–8 at.%), the risk of solidification cracking is significantly reduced, achieving a balance between grain refinement and hot‐crack inhibition. Moreover, as shown in Figure 1D, based on the solidification of the solid‐bridge, when Cu content is in the range of 11–12 wt.% (∼5 at.%), adequate eutectic phases rapidly form from the coherent point (*f*_coh_), generating high‐strength solid‐bridges that suppress crack‐tip stress at dendrite roots and stress accumulation during the final stage of solidification, facilitating crack‐free printing [41, 42, 43]. Before evaluating the evaporation rate, the Mg/Zn ratio is adjusted to promote the precipitation of η‐MgZn_2_, which is thermodynamically stable and provides significant precipitation strengthening [44, 45]. Within the limited compositional space defined by the final evaporation rate calculations, we avoid uneven composition in the melting pool and burn‐out defects caused by high contents of low‐melting‐point elements, ultimately selecting Al_85_Cu_5_Li_4_Mg_3_Zn_3_ as the preferred alloy (Figure 1E).

### 2.2 Microstructure Characterization

First, we prepared as‐cast Al_85_Cu_5_Li_4_Mg_3_Zn_3_ LAEA by vacuum suspension melting the raw material particles three times, and Figure S5A (Supporting Information) shows the microsized coarse eutectic phases. The time‐of‐flight secondary ion mass spectrometry (TOF‐SIMS) images reveal oxidation on the powder surface, which is difficult to avoid completely due to the presence of highly oxidizable Al, Li, and Mg (Figure S6, Supporting Information). However, we control the oxygen content in powders to as low as ∼0.044 wt.% to prevent solidification defects in the melting pool caused by severe oxidation. Subsequently, the as‐printed Al_85_Cu_5_Li_4_Mg_3_Zn_3_ LAEA, fabricated with 220 W laser power, refines the microsized eutectic networks into the nanosized cellular eutectic networks and eliminates compositional bias at the microsized level (Figure S5A,B, Supporting Information). The as‐cast and as‐printed samples were analyzed by X‐ray diffraction (XRD) to reveal their phase compositions (Figure S5C, Supporting Information). It is evident that SLM increases the content of supersaturated α‐Al and the maximum solid solubility of solute elements, especially Cu. Additionally, there is a difference in phase type, with the transformation from the R phase‐Al_5_CuLi_3_ with body‐centered cubic structure in the as‐cast alloy to the quasicrystalline T2 phase‐Al_6_CuLi_3_ in the as‐printed alloy, revealing that the as‐printed alloy achieves an unprecedented microstructure and phase change in LAEAs. This phase selection behavior is explicitly supported by our CALPHAD thermodynamic calculations (Figure S7, Supporting Information), which differentiate the dominance of the stable R phase in the equilibrium state (analogous to casting) from the metastable T2 phase in the nonequilibrium rapid solidification state (analogous to SLM).

**FIGURE 2:**
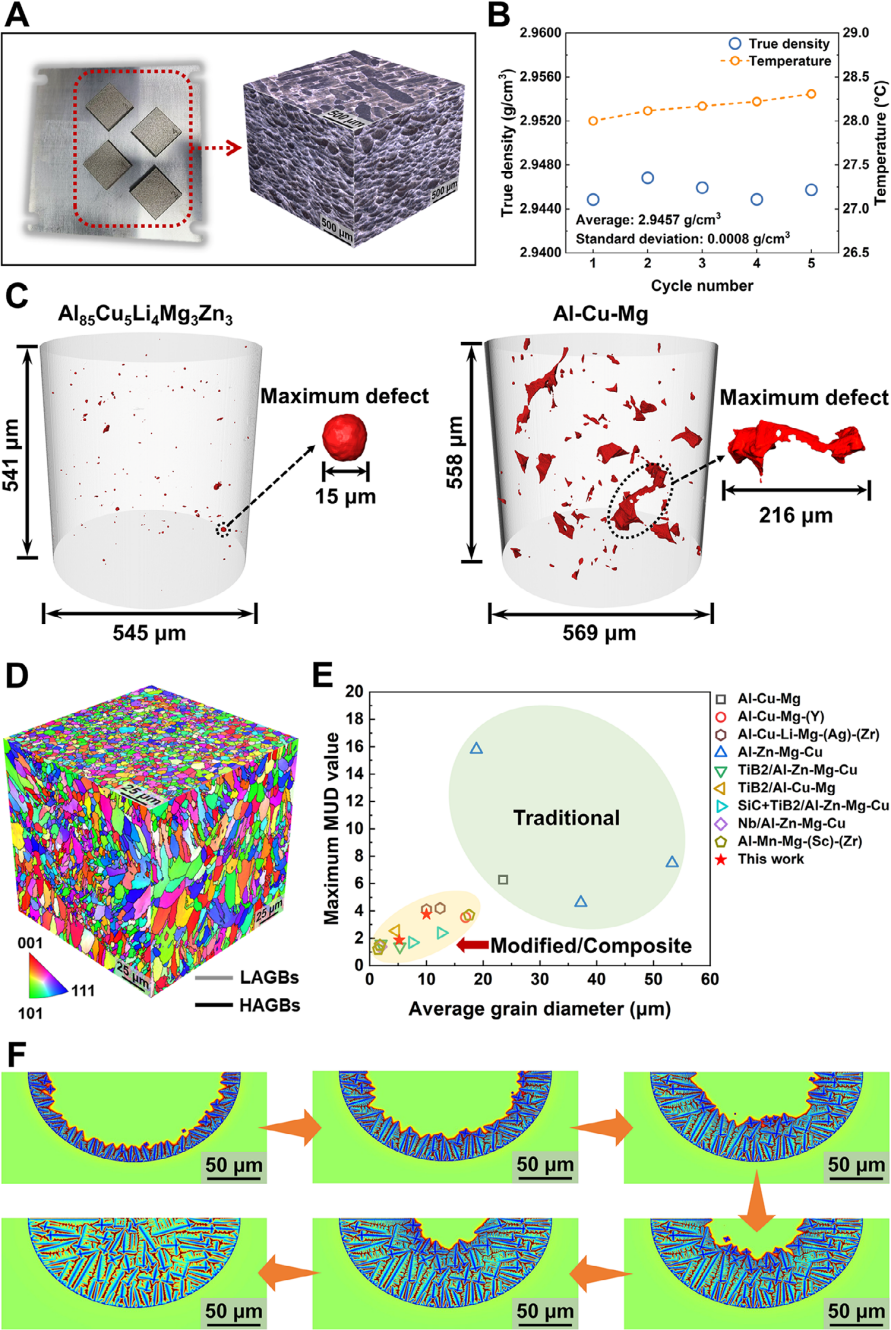
Characterization of formability and orientation of the as‐printed Al_85_Cu_5_Li_4_Mg_3_Zn_3_ lightweight Al‐based entropy alloy (LAEA). A) Macroscale and optical microscopy (OM) images of the as‐printed bulk. B) Results of the true density test. C) µ‐CT analysis showing the spatial distribution of printed defects in Al_85_Cu_5_Li_4_Mg_3_Zn_3_ LAEA and Al‐Cu‐Mg alloy, respectively. D) 3D morphology of the electron backscatter diffraction (EBSD) inverse pole figure (IPF) maps. E) Maximum multiple of uniform distribution (MUD) value and average grain diameter of the present results versus previously published traditional Al alloys [51, 52, 53, 54], and modified/composite Al alloys [51, 52, 53, 54, 55, 56, 57, 58]. F) Simulation of dendrite growth in the melting pool.

To minimize stress accumulation during 3D printing, an interlayer rotation of 67° and an island scanning strategy are used to fabricate Al_85_Cu_5_Li_4_Mg_3_Zn_3_ LAEA [46]. Optical microscopy (OM) images reveal the excellent printability of the alloy, with dense melting pools and scanning paths, as shown in Figure 2A. The as‐printed alloy achieves a true density of approximately 2.95 g/cm^3^, aligning with the initial lightweight design objective (Figure 2B). High‐resolution X‐ray microcomputed tomography (µ‐CT) measurements in Figure 2C reveal the internal defects. Quantitative volumetric analysis of the µ‐CT data (Figure S8, Supporting Information) substantiates the high structural integrity of the as‐printed alloy. The Al_85_Cu_5_Li_4_Mg_3_Zn_3_ LAEA exhibits an extremely low porosity of 0.01% (corresponding to a relative density of 99.99%). The defects are confined to a narrow size range with a maximum equivalent diameter of 15 µm and an average value of 3.65 µm. Crucially, sphericity analysis reveals that these defects are predominantly spherical (sphericity: 0.6–1.1), identifying them as inevitable gas entrapment defects characteristic of the SLM process rather than solidification cracking. In stark contrast, the traditional as‐printed Al‐Cu‐Mg control alloy displays a significantly higher porosity of 0.3% and contains numerous large irregular defects (with a maximum length of 216 µm, a maximum equivalent diameter of 70 µm, and a sphericity of 0.4), which are typical of interconnected hot cracks. These quantitative metrics rigorously confirm that the eutectic solidification strategy has effectively eliminated the hot‐cracking mechanism. In contrast, the traditional as‐printed Al‐Cu‐Mg alloy always shows numerous microcracks with the maximum defect up to 216 µm. Electron backscatter diffraction (EBSD) images show the near‐equiaxed grain morphology in as‐printed Al_85_Cu_5_Li_4_Mg_3_Zn_3_ LAEA, which is extremely rare in Al alloys without the introduction of inoculation treatment (Figure 2D). A threshold of 5 µm is used to distinguish coarse grains (CG) from fine grains (FG) within the heat‐affected zone [47, 48]. Attributed to the grain refinement (<10 µm) induced by near‐equiaxed grain growth, a weaker bimodal distribution is observed, instead of a more heterogeneous grain distribution, with up to ∼95% high‐angle grain boundaries (HAGBs), as shown in Figure S9 (Supporting Information). The uniform networks of HAGBs act as a stress buffer, providing greater mobility at high temperatures. This disperses thermal stress and provides more “dislocation pathways”, thereby reducing localized stress concentrations [43, 49, 50]. Additionally, the weak texture of the pole figures demonstrates the random grain orientation and isotropy of the as‐printed alloy, with a maximum multiple of uniform distribution (MUD) value of only 3.77 along the building direction, significantly lower than 24.26 for the as‐cast alloy (Figure S10, Supporting Information). XRD analysis of the texture in the more macroscale area showed maximum MUD values similar to those obtained by EBSD, further confirming the weak texture tendency of the as‐printed alloy (Figures S11 and S12, Supporting Information). Figure 2E highlights another advantage of the newly developed alloy here, achieving grain refinement and isotropy comparable to as‐printed modified/composite Al‐based alloys. The dendrite growth simulation in the melting pool shows that the solutes are mainly concentrated in the dendrite roots and grain boundaries (Figure 2F). Over time, the growth rate of dendrite tips stabilizes, transitioning from initially rapid columnar growth to finer near‐equiaxed growth, consistent with the EBSD results.

**FIGURE 3:**
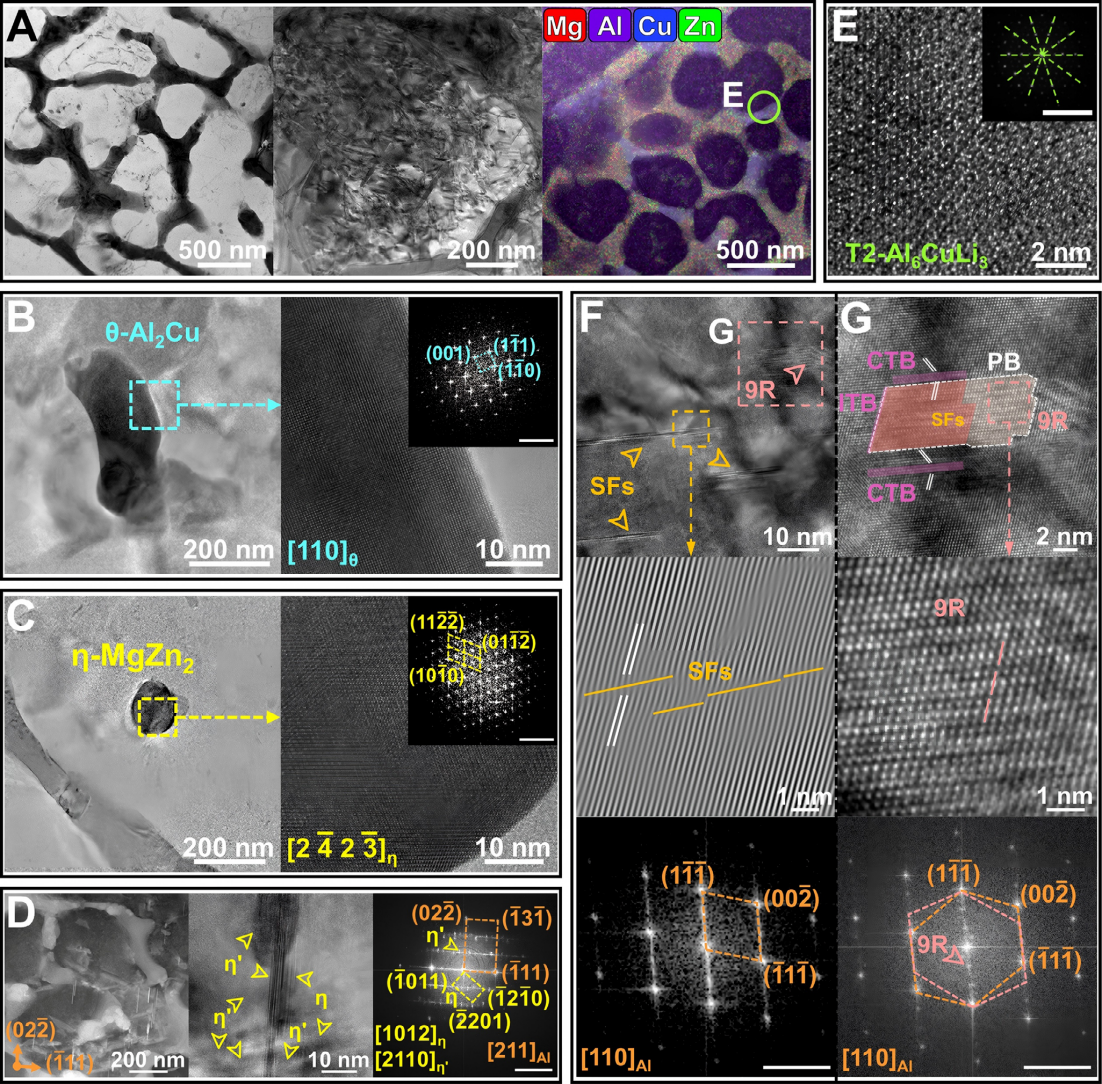
Transmission electron microscopy (TEM) characterization of the as‐printed Al_85_Cu_5_Li_4_Mg_3_Zn_3_ lightweight Al‐based entropy alloy (LAEA). A) Bright field (BF) images showing sparse dislocation lines and high‐density dislocations, respectively, along with an energy dispersive spectroscopy (EDS) map of the nanosized cellular eutectic networks. high‐resolution TEM (HRTEM) images of B) θ‐Al_2_Cu and η‐MgZn_2_ C), with enlarged areas and corresponding fast Fourier transform (FFT) patterns. The scale bar of FFT patterns is 5 nm^−1^, and the same below. D) HAADF–STEM image showing the rod‐like precipitates under the [211]_Al_ zone axis, where the HRTEM image and FFT pattern demonstrating the existence of the semi‐coherent η′ phases. E) The enlarged HRTEM image of the orange square in (A) showing the T2‐Al_6_CuLi_3_, with the corresponding FFT pattern. F) HRTEM image shows the nanosized planar defects, stacking faults (SFs), and 9R phase. Numerous stacking fault ribbons of atomic planes in the Inverse FFT (IFFT) pattern and elongated diffraction fringes along the {111} plane in the FFT pattern reveal the details of SFs. G) HRTEM image highlighting the square in (F) reveals the SF‐decorated 9R phase obtained by ITB dissociation, along with the surrounding coherent twin boundaries (CTBs). The white dashed line indicates the phase boundaries (PB). The enlarged HRTEM image demonstrates the periodic stacking structure of the 9R phase, and the FFT pattern shows the characteristic diffraction spots of the 9R phase and twinning relationship.

**FIGURE 4:**
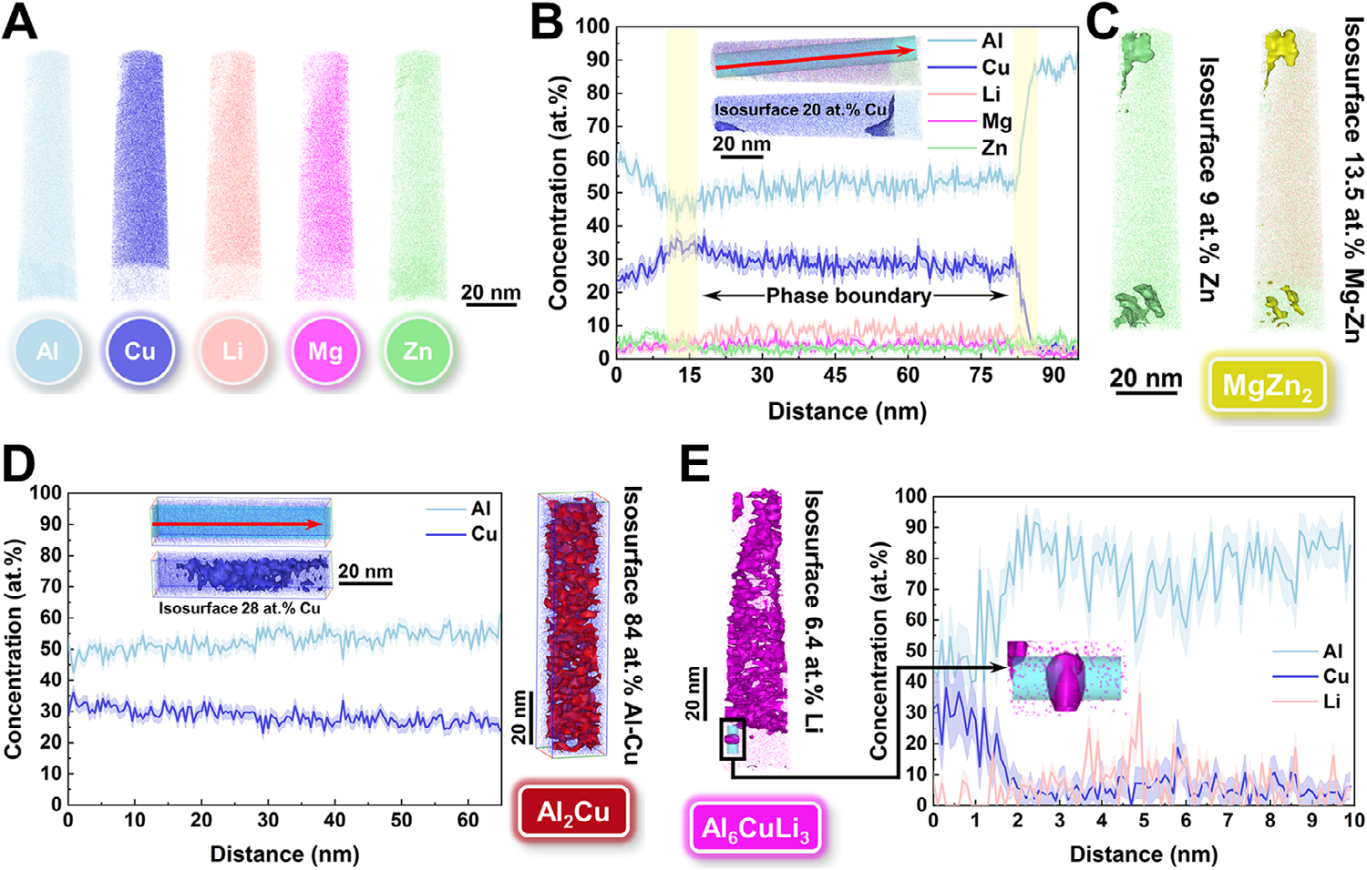
Atom probe tomography (APT) characterization of the nanosized eutectic phases and matrix. A) 3D reconstruction of atomic maps showing heterogeneous elemental distribution. B) 1D concentration profiles obtained from top to bottom along the cylinder of interest, with error intervals showing the standard error of the plotted concentrations. The significant fluctuations of Al and Cu elements reveal two PB, approximately defined by the 20 at.% Cu isosurface. C) 9 at.% Zn and 13.5 at.% Mg‐Zn isosurfaces demonstrating the eutectic phase at the top of the APT tip as MgZn_2_, which is also aligned parallel in the matrix. D) 1D concentration profiles obtained from top to bottom along the cube of the Cu‐enriched region, with error intervals showing the standard error of the plotted concentrations. The 28 at.% Cu isosurface shows the average concentration in the Cu‐enriched region, which is further confirmed as Al_2_Cu by the 84 at.% Al‐Cu isosurface. E) 1D concentration profiles obtained from top to bottom along the cylinder of a particle in the matrix, with error intervals showing the standard error of the plotted concentrations. The localized fluctuations of Li and 6.4 at.% Li isosurface collectively confirm the particle as Al_6_CuLi_3_.

Transmission electron microscopy (TEM) measurements of the studied samples are performed in Figure 3. The bright‐field image and mapping results from high‐angle annular dark field (HAADF) scanning transmission electron microscopy (STEM) show Mg/Cu/Zn‐enriched nanosized cellular eutectic phases and incoherent nanoprecipitates within the matrix (Figure 3A). Li detection is precluded by the limited sensitivity of energy dispersive spectroscopy (EDS) toward light elements. The sparse dislocation lines confirm the low internal stress and high fraction of HAGBs in the as‐printed alloy, with high‐density dislocations found only in few nanosized cellular eutectic networks. Incoherent nanoprecipitates share the same phase constitution as nanosized cellular eutectic networks, comprising θ‐Al_2_Cu (Figure 3B), η (Figure 3C), and T2 (Figure 3E) phases. To characterize finer coherent/semi‐coherent nanoprecipitates, we further conducted measurements along [100], [110], and [211] zone axes. Intriguingly, the high‐Cu (∼11 wt.%) alloy in this work deviates from the precipitation sequence of traditional Al‐Cu alloys by not forming the semicoherent *θ*' phase, instead following the precipitation sequence of Al‐Zn‐Mg‐Cu alloys to form the semi‐coherent η′ phase. Figure 3D combines HAADF–STEM with high‐resolution TEM (HRTEM) images to reveal rod‐like η′ phases nucleating on {111}_Al_ planes and elongating along {022}_Al_ planes under the [211]_Al_ zone axis, with diffraction spots located at 1/3 {022}_Al_ and 2/3 {022}_Al_. Coherent Guinier–Preston (GP) zones are almost absent due to prolonged substrate heating during the printing process. A site‐specific atom probe tomography (APT) sample was prepared by focused ion beam (FIB) milling perpendicular to the eutectic phase toward the matrix (Figure S13, Supporting Information) and reconstructed to obtain 3D elemental mappings, as shown in Figure 4. The single atom map in Figure 4A shows strong enrichment of Cu, Li, and Mg in the eutectic phase, while Zn is slightly enriched in the matrix. The 20 at.% Cu isosurface delineates η/*θ* and *θ*/matrix phase boundaries (PB), with Li‐Mg co‐segregation observed in the eutectic *θ* phase (Figure 4B–D). Additionally, the parallel‐aligned η′/η phases and granular T2 phase in the matrix are also shown in Figure 4C,E. The results from TEM and APT suggest that Cu, with a low diffusion coefficient, is primarily involved in the formation of the nanosized cellular eutectic phases, which is even lower than Zn in the matrix, allowing Zn to dominate the precipitation sequence of η phase. Local Cu fluctuations in the matrix trigger eutectic *θ* phase nucleation through composition‐driven liquid layer formation, bypassing the solute precipitation sequence.

The extreme solidification conditions in SLM, coupled with solute elements exhibiting low stacking fault energy (SFE, Mg: ∼34 mJ/m^2^, Cu: ∼45 mJ/m^2^), overcome the energy barrier of the high‐SFE Al matrix (∼120–144 mJ/m^2^), leading to unexpected formation of nanosized planar defects, including high‐density SFs, nanotwin boundaries, and 9R phases [59, 60]. The FFT and IFFT patterns of HRTEM image (Figure 3F) reveal the detailed features of the SFs in terms of atomic stacking defects with elongated diffraction fringes along {111} planes. The 9R phase, containing three Shockley partials (one edge and two mixed partials) on adjacent {111} planes, is clearly visible in Figure 3G, resulting from the dissociation of the incoherent twin boundaries (ITBs) into tilt boundaries (PBs) and repeated migration [61]. Coherent twin boundaries (CTBs) near the 9R phase are also detected. Chemical fluctuations of low‐SFE solutes and complex stress fields synergistically stabilize the 9R phase with ITBs and associated SFs, creating enhanced dislocation barriers and potential strain‐hardening sources [62, 63].

### 2.3 Mechanical Properties

**FIGURE 5:**
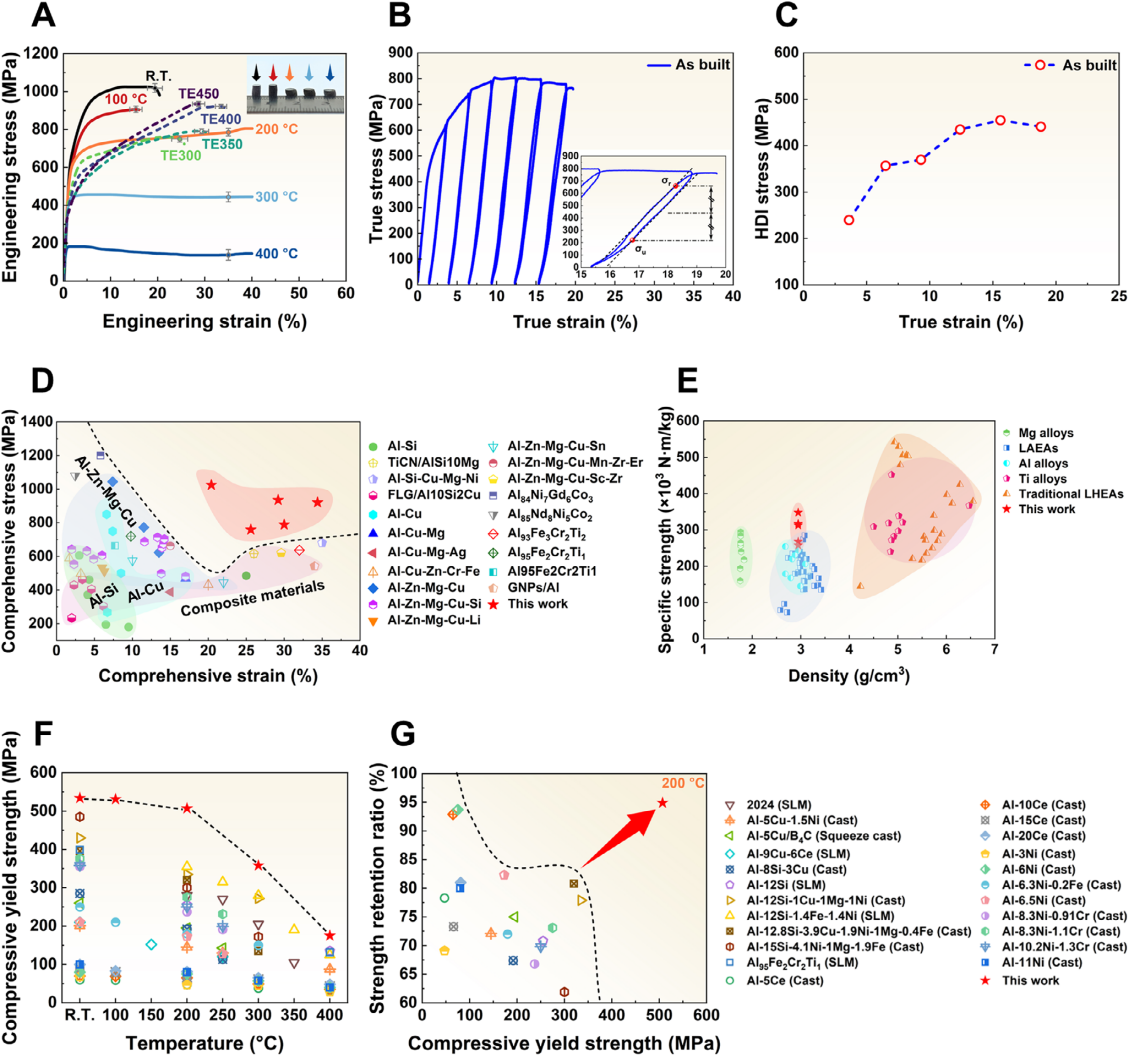
Macroscale mechanical properties of as‐printed and thermal‐exposed Al_85_Cu_5_Li_4_Mg_3_Zn_3_ lightweight Al‐based entropy alloys (LAEAs) under compression tests. A) Compressive engineering stress–strain curves of the as‐printed Al_85_Cu_5_Li_4_Mg_3_Zn_3_ LAEA at room temperature and high temperatures (100°C, 200°C, 300°C, and 400°C), as well as the curves of thermal‐exposed (300°C, 350°C, 400°C, and 450°C) alloys at room temperature. B) Loading‐unloading‐reloading curves of the as‐printed Al_85_Cu_5_Li_4_Mg_3_Zn_3_ LAEA, with the inset showing the mechanical hysteresis loop after unloading‐reloading cycle with an unloading strain of 18.5%. The sample was first cycled at 3.5% strain and subjected to cyclic compressive loading at 3% strain until failure. C) Evolution of heterogeneous deformation‐induced (HDI) stress corresponding to different true strains in (B). D) Comparison of the compressive properties between the present results and previously published Al‐Si series [65, 66, 67], Al‐Cu series [65, 68], Al‐Zn‐Mg‐Cu series [20, 69, 70, 71, 72], Al‐based composite materials [73, 74, 75], and other Al‐based alloys [21, 51, 76, 77, 78, 79, 80]. E) Specific strength and density of present results versus previously published Mg alloys [81, 82, 83, 84], Al alloys [21, 51, 67, 71, 76, 77], LAEAs [18, 20, 21, 25, 85, 86], Ti alloys [87, 88, 89, 90, 91], and traditional LHEAs [18, 27, 92]. F) and G) Comparison of the temperature dependence of compressive yield strength, and comparison of compressive yield strength retention ratio at 200°C between present results and previously published Al alloys [76, 93, 94, 95, 96, 97, 98, 99, 100, 101, 102, 103, 104].

Figure 5A and Figure S14 (Supporting Information) show the compressive engineering stress–strain curves of as‐printed and as‐cast Al_85_Cu_5_Li_4_Mg_3_Zn_3_ LAEAs. The as‐cast alloy exhibits catastrophic failure at ∼6.5% compressive strain, showing a compressive strength of 600 MPa. In contrast, the nanoreinforced phases and planar defects introduced by SLM synergistically enhance the strength and plasticity. This combination of GPa‐grade strength and significant plasticity represents a definitive breakthrough, directly resolving the severe compressive strength‐ductility trade‐off that has historically limited cast LAEAs. The ultrastrong compressive strength of the as‐printed alloy exceeds 1000 MPa, with yield strength reaching ∼534 MPa and compressive strain elevated to ∼20%. To further validate the robustness of the HTC framework, we have designed and fabricated three novel candidate alloys (Al_87_Cu_5_Li_3_Mg_2_Zn_3_, Al_80_Cu_5_Li_7_Mg_4_Zn_4_, and Al_88_Cu_2_Li_4_Mg_3_Zn_3_), including positive validation and negative controls (Figure S15, Supporting Information). The Al_87_Cu_5_Li_3_Mg_2_Zn_3_ alloy was selected as the neighboring composition within the optimal window, exhibiting a similar dense microstructure and excellent mechanical property, thereby confirming the robustness of the design. In addition, the Al_80_Cu_5_Li_7_Mg_4_Zn_4_ and Al_88_Cu_2_Li_4_Mg_3_Zn_3_ alloys were designed as negative controls to test the boundaries of our framework. The HTC framework has marked them as evaporative risk and high cracking sensitivity, respectively. Experimental results confirm the presence of extensive and unavoidable pores in the Al_80_Cu_5_Li_7_Mg_4_Zn_4_ alloy, and severe microcracks in the Al_88_Cu_2_Li_4_Mg_3_Zn_3_ alloy, making it difficult for either alloy to achieve 10% compressive strain. These results form a complete evidence loop, demonstrating that our integrated framework not only precisely locates preferred regions with high statistical confidence but also accurately delineates physical processing boundaries. Generally, the main strengthening methods for Al alloys by AM include grain boundary strengthening induced by inoculation and precipitation strengthening promoted by aging, while heterogeneous strengthening, relying on bimodal grain structures or cellular eutectic networks, often suffers from either insufficient strength or limited eutectic ductility [20, 57]. The true stress–strain curve for reloading and unloading in Figure 5B shows clear hysteresis loops, and the specific HDI stress (*σ*_HDI_) can be calculated using the following equation: [64]

(1) $$\sigma_{\mathrm{HDI}}=\frac{\sigma_{u}+\sigma_{r}}{2}$$

where *σ*_u_ is the unloading yield stress, and *σ*_r_ is the reloading yield stress. Figure 5C illustrates the progressive HDI stress accumulation, particularly during the nonuniform plastic deformation stage, where sustained HDI stress (>400 MPa) contributes to significant work hardening. To provide a comprehensive evaluation, tensile properties are characterized (Figure S16, Supporting Information). The as‐printed alloy delivers a high tensile yield strength of ∼501 MPa and an ultimate tensile strength of ∼589 MPa. The tensile elongation is ∼2.3%, revealing a notable tension‐compression asymmetry compared to the ∼20% compressive plasticity. It is crucial to note that this limited tensile ductility is not attributed to printing defects, as evidenced by the near‐zero porosity (0.01%) confirmed via µ‐CT. Instead, it reflects the intrinsic nature of intermetallic‐reinforced LAEAs. Traditional LAEAs (represented by the as‐cast state here) typically suffer from catastrophic brittle failure due to coarse intermetallics. Although our strategy refines these phases into nanosized cellular eutectic phases to unlock breakthrough compressive plasticity, these continuous rigid networks still govern the tensile fracture by serving as stress concentration sites. This behavior reflects a characteristic trade‐off within this alloy system, where the interconnected hard skeleton, while critical for superior heat resistance and printability, restricts tensile ductility. To further explore the mechanical properties of the as‐printed Al_85_Cu_5_Li_4_Mg_3_Zn_3_ LAEA after thermal exposure and the effect of the controllable phase transformation mechanism found, the compression tests of the alloy thermal exposed at 300°C (TE300), 350°C (TE350), 400°C (TE400), and 450°C (TE450) for 100 h are carried out, as shown in Figure 5A. It is worth noting that thermal exposure fully activates superior compressive plasticity, especially for TE400 alloy achieving ∼34%. In addition, starting from the TE400 alloy, the compressive strength shows a jump growth, with the TE450 alloy achieving a maximum compressive strength of ∼935 MPa. Regarding the above phenomenon, we will discuss it in detail later in connection with the controllable phase transformation mechanism. Figure 5D compares the compressive properties of the as‐printed and thermal‐exposed Al_85_Cu_5_Li_4_Mg_3_Zn_3_ LAEA with those of reported representative Al alloys and Al‐based composite materials. Heterogeneous nucleation or composite strategies are used to effectively address the strength‐plasticity trade‐off in Al alloys up to 600 MPa. Our optimally constructed as‐printed Al_85_Cu_5_Li_4_Mg_3_Zn_3_ LAEA, fabricated via SLM, introduces nanoreinforced phases and planar defects that surpass this threshold, achieving bulk deformability with GPa‐grade strength. Figure 5E highlights the superior specific strength (∼350 × 10^3^ N m/kg) of this alloy over existing Al alloys, even rivaling some titanium alloys and traditional lightweight high‐entropy alloys (LHEAs). Here, we have also evaluated the high‐temperature compression properties of the as‐printed alloy (Figure 5A), specifically noting that no fracture occurs starting from 200°C (compression strain terminated at 40%) and that it still shows a compressive strength >800 MPa at 200°C. Meanwhile, we have compared the high‐temperature compression yield strength and its retention ratio with Al‐based alloys prepared by various methods previously reported, which clearly reveal that the as‐printed Al_85_Cu_5_Li_4_Mg_3_Zn_3_ LAEA exhibits superior heat resistance (Figure 5F,G). The yield strength of the as‐printed Al_85_Cu_5_Li_4_Mg_3_Zn_3_ LAEA at 200°C is 507 MPa, with a yield strength retention ratio of 95%, significantly surpassing existing Al‐based alloys.

## 3 Discussion

### 3.1 Excellent Printability and Eutectic Strategy

The cracking susceptibility of SLM‐processed high‐strength Al alloys arises from a large solidification range and unstable undercooling affected by the high thermal gradient and high solidification rate ratio (*G*/*R*) [6, 105]. First, according to Rappaz's criterion, the expanded mushy zone increases the possibility of thermal stress and solidification shrinkage creating weaknesses in the liquid channels of inter‐dendritic regions, which can easily lead to the formation of cavities and hot cracks when the critical stress threshold is exceeded [4, 106]. Crack‐susceptible alloys are often simply attributed to having a wide solidification range, but the critical factor is the terminal solidification stage and whether the solidification curves turn sharply at high solid fractions [4, 107]. Previous HTC considers the influence of Cu, the key element for dendritic solidification in the solid‐bridge. The alloy is designed with an Al‐Cu binary near‐eutectic composition, ensuring sufficient terminal eutectic during the coherent stage of solidification (*f*_coh_ < *f*_s_ < *f*_brid_, here we set *f*_coh_ = 0.75 and *f*_brid_ = 0.94 in calculations), which allows for a near‐zero solidification range and maximum control over thermal shrinkage and stress [41]. Similarly, the subsequent bridging stage of solidification (*f*_brid_ < *f*_s_ < 1) reduces stress accumulation and provides sufficiently high strength to resist critical stress for hot cracking. The solidification curves (Figure S17A, Supporting Information) calculated via JmatPro software based on the Scheil‐Gulliver model show that, compared with Al‐Cu‐Mg and Al‐Zn‐Mg‐Cu alloys (both considered to be crack‐susceptible alloys), the Al_80_Cu_5_Li_4_Mg_3_Zn_3_ LAEA's curve undergoes a sharp turnover before *f*_coh_ at a lower solid fraction (before this temperature, there is sufficient liquid supply to refill hot cracks), thereby avoiding severe thermal shrinkage during the terminal solidification.

In addition, under the predominant solidification conditions of SLM (high *G*/*R* ratio), Al‐Cu‐Mg and Al‐Zn‐Mg‐Cu alloys tend to form coarse columnar grains, which increases the risk of crack initiation and propagation during the terminal solidification [54, 108]. In contrast, equiaxed grains are more likely to raise the critical stress required for hot crack initiation by increasing grain boundary density and offering more tortuous paths for crack propagation [109]. Therefore, to promote the columnar to equiaxed transition (CET) as a means of mitigating cracking risk, heterogeneous nucleation and growth‐restriction effects are typically considered [110, 111]. Heterogeneous particles and solutes with a high growth‐restriction factor (*Q* value) enhance constitutional undercooling ahead of the solid/liquid interface and promote rapid solidification, leading to a reduced *G*/*R* ratio and thereby facilitating the CET theory [105, 112]. Quested et al. [113] proposed that the optimal method to calculate the *Q* value is through the initial constitutional undercooling efficiency derived from the thermodynamic model, which can be calculated using the following equation:

(2) $$Q={\left(\frac{\partial \left(\Delta T_{\mathrm{CS}}\right)}{\partial f_{s}}\right)}_{f_{s}\to 0}$$

where Δ*T*_CS_ is the constitutional undercooling, *f*_s_ is the solid fraction, both obtained from the Scheil curves. As shown in Figure S17B (Supporting Information), the *Q* value of Al_85_Cu_5_Li_4_Mg_3_Zn_3_ LAEA is calculated to be 58.5 K, which is significantly higher than that of Al‐Cu‐Mg (*Q* = 18.9 K) and Al‐Zn‐Mg‐Cu alloys (*Q* = 15.7 K). Sing et al. [114] summarized the growth‐restriction factors of different elements in Al alloys, showing that Mg (103.3 K), Cu (93.7 K), and Zn (48.0 K) have high growth‐restriction effects. Du et al. [115] and Chen et al. [116] also pointed out that Cu, as a high‐*Q*‐value element, can enhance constitutional undercooling and promote grain refinement when added. In this work, although no additional heterogeneous nucleation particles with strong growth‐restriction effects are introduced, the high Cu content (∼11 wt.%) still activates the CET theory to a certain extent, enabling the transformation of primary α‐Al from a dendritic structure to a cellular structure and promoting near‐equiaxed grain growth during the incoherent solute stage, as shown in Figures 2D,F and 3A.

Subsequently, relying on the brief concentration of nucleation and growth of numerous eutectic phases at terminal solidification (Figure S17C, Supporting Information), Al_85_Cu_5_Li_4_Mg_3_Zn_3_ LAEA exhibits almost no thermal shrinkage during the coherent and bridging stages of solidification, whereas the crack‐susceptible alloys are easily affected by significant thermal shrinkage due to insufficient liquid refilling, thereby generating hot cracks. Specifically, the Scheil‐Gulliver simulation (Figure S7B, Supporting Information) reveals that under rapid cooling, the solidification path favors the formation of the metastable T2 phase alongside Al_2_Cu and MgZn_2_, rather than the equilibrium R phase. Crucially, compared to the equilibrium R phase, the T2 phase with a higher phase transformation temperature participates in the eutectic reaction at terminal solidification. This reaction shortens the temperature range and thereby suppresses the tendency for hot cracking, validating the eutectic strategy for printability. Kou et al. [117] proposed a hot cracking criterion by improving the model of Rappaz et al. to describe the dynamic balance between the strain rate of the solid and the feeding rate of the liquid during the terminal solidification:

(3) $$\begin{array}{c} {\left\{\frac{d\varepsilon_{\mathrm{local}}}{\mathrm{dt}}\;>\;\sqrt{1\;-\;\beta }\frac{d\sqrt{f_{s}}}{\mathrm{dT}}\;\frac{\mathrm{dT}}{\mathrm{dt}}+\frac{d}{\mathrm{dz}}\;\left[\left(1\;-\;\sqrt{1\;-\;\beta }\sqrt{f_{s}}\right)v_{z}\right]\right\}}_{\sqrt{f_{s}}\to 1} \end{array}$$

where ε_local_ is the local tensile strain, *t* is time, *β* is the solidification shrinkage rate, *T* is temperature, *z* is the temperature axis of the columnar dendrite, and *v*_z_ is the liquid filling rate along the grain boundary. When the separation rate between grains exceeds the sum of the growth rate and the liquid filling rate, cracking tends to occur. Based on this equation, Kou et al. [118] proposed the crack susceptibility index (CSI), which is calculated by taking the maximum absolute value of the derivative of temperature with respect to the square root of the solid fraction (*f*_s_^1/2^) during the terminal solidification:

(4) $$\begin{array}{c} \mathrm{CSI}=\max{\left|\frac{\mathrm{dT}}{d\sqrt{f_{s}}}\right|}_{\sqrt{f_{s}}\to 1} \end{array}$$

The *T*‐*f*_s_^1/2^ curves calculated according to the Scheil curves are summarized in Figure S17D (Supporting Information). The curves terminate at *f*_s_^1/2^ = 0.99 because the correlation between crack susceptibility and |d*T*/d(*f*_s_)^1/2^| only extends to this point [119]. The change in |d*T*/d(*f*_s_)^1/2^| from the coherent stage to the bridging stage is shown in Figure S17E (Supporting Information), as well as the marked CSI values, where low CSI values are typically considered to indicate low cracking tendency. The CSI value of Al_85_Cu_5_Li_4_Mg_3_Zn_3_ LAEA is only 1181°C, and it is briefly concentrated in the initial coherent stage. However, the |d*T*/d(*f*_s_)^1/2^| values for crack‐susceptible alloys Al‐Cu‐Mg and Al‐Zn‐Mg‐Cu increase sharply during the solid‐bridging stage, where liquid feeding is insufficient, resulting in high CSI values of 5500°C and 7500°C, respectively. Further, grain refinement is considered to correlate with the thinning of the liquid film. The critical stress for hot cracking initiation (*σ*_c_) corresponds to the capillary pressure at the interface between adjacent grains, which can be calculated using the following equation: [120]

(5) $$\begin{array}{c} \sigma_{c}=\frac{8\gamma }{\sqrt{3}d}\;\left(\frac{f_{s}^{M}}{1-f_{s}^{M}}\right) \end{array}$$

where γ is the surface tension of the liquid, which can be calculated by the JmatPro software to determine how *γ* changes with temperature during the terminal solidification (*f*_s_ from 0.9 to 0.99). *d* is the grain size, where columnar grains refer to grain width and equiaxed grains refer to average grain size. *M* is the microstructural parameter, with columnar grains and equiaxed grains being 1/3 and 1/2, respectively. Compared with crack‐susceptible alloys, Al_85_Cu_5_Li_4_Mg_3_Zn_3_ LAEA exhibits a higher critical stress during the terminal solidification, especially when *f*_s_ ≥ 0.96, where the critical stress increases exponentially, indicating that sufficient solid bridge strength is present to suppress hot crack initiation (Figure S17F, Supporting Information). In summary, the as‐printed Al_85_Cu_5_Li_4_Mg_3_Zn_3_ LAEA fabricated in this work demonstrates the advantages of a simple, effective, and stable eutectic solidification strategy.

### 3.2 Deformation Mechanisms at Room Temperature and High Temperatures

Furthermore, micropillar compression tests are conducted in the equiaxed grain zone (EGZ) within the melting pool and overlapping heat‐affected zone (HAZ) (Figure S18A, Supporting Information). The state of the pillars under various displacements and the engineering stress–strain curves, as depicted in Figure S18B,C (Supporting Information). Both pillars exhibit yield strengths exceeding 700 MPa, indicating that, due to the highly homogenous microstructure, the strength contribution mechanisms (solid solution strengthening, precipitation strengthening, grain boundary strengthening, and HDI strengthening) in the two zones are not significantly different. The weak bimodal feature means that the difference in grain diameter (∼8 µm) has negligible effects on grain boundary strengthening in different zones, but does affect the post‐yield plastic deformation behavior. Dislocation accumulation and bypassing activate the wrinkled slip bands layer by layer, avoiding continuous large deformation at the local weak interfaces and promoting homogeneous slip [121]. Interestingly, neither pillar shows obvious shear features prematurely, until reaching 25% (Pillar 1) and 50% (Pillar 2), with fracture occurring shortly thereafter. The denser destabilization of GBs leads to earlier fracture, so that pillar 1 in the HAZ breaks after compression to ∼30%, yet still maintains a compressive strength of ∼1200 MPa. In contrast, pillar 2 in the EGZ shows superior plastic strain of ∼60% and a bulging feature, with ultimate compressive strength even reaching ∼1700 MPa. In summary, the ultrastrong strength and homogeneous slip in the microzone create the conditions necessary for macroscale ultrastrong strength and plastic deformation.

**FIGURE 6:**
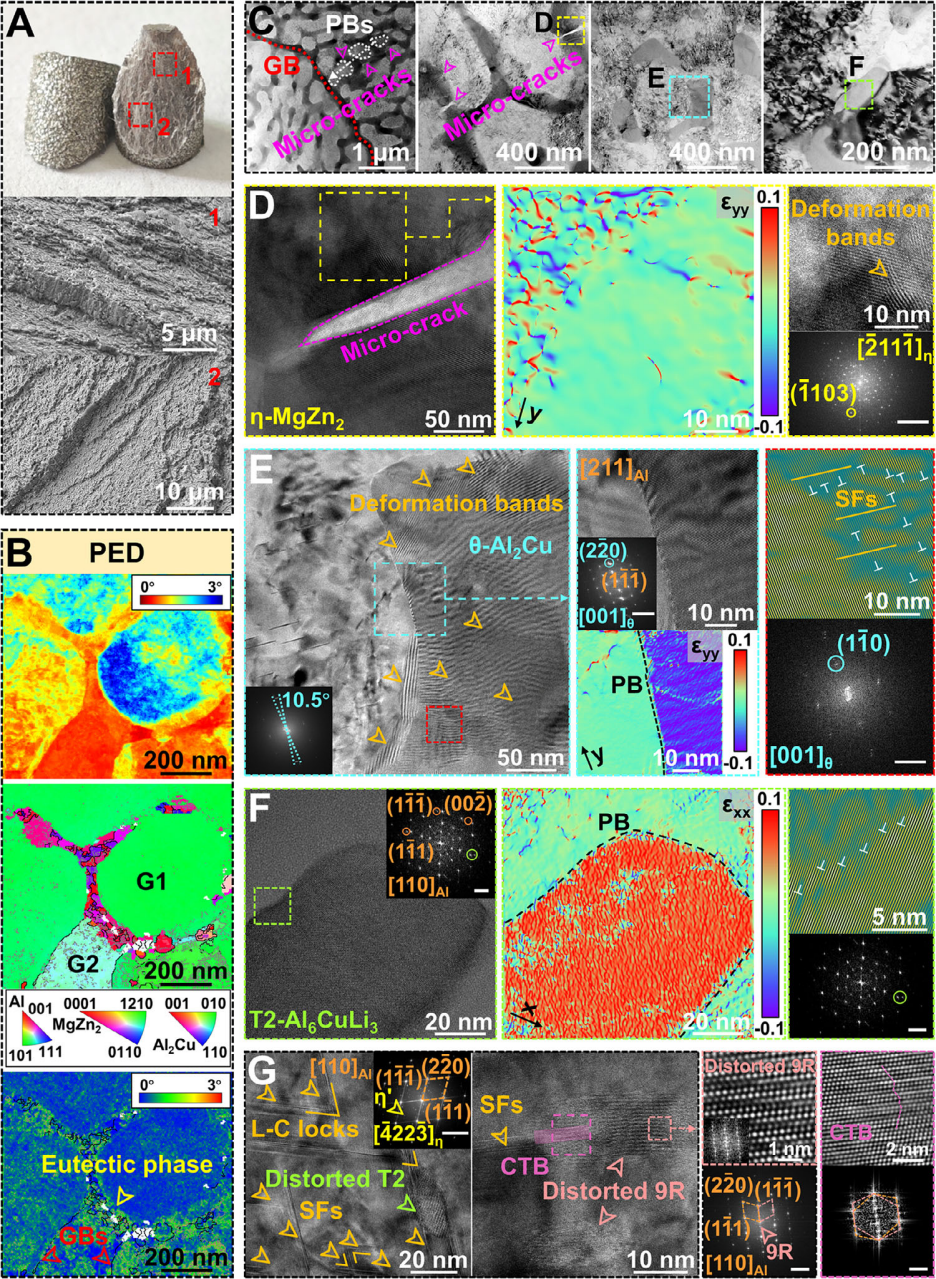
Multiscale characterization of compressive deformation behavior of the as‐printed Al_85_Cu_5_Li_4_Mg_3_Zn_3_ lightweight Al‐based entropy alloy (LAEA) at room temperature. A) Macroscale fracture morphology and SEM micrographs of two magnified regions. B) Precession electron diffraction (PED) analysis of the nanosized eutectic networks, with an inserted band contrast (BC) map, inverse pole figure (IPF) map, and KAM map. C) Bright field (BF) images showing transmission electron microscopy (TEM) deformation characteristics, including intact GB and successively cracked PBs toward GB. The enlarged images highlight the microcracks in the nanosized eutectic networks and the hard phases with abundant dislocations at the interfaces. D) The enlarged high‐resolution TEM (HRTEM) image of the yellow square in (C) showing the cracked eutectic *η* phase. Geometric phase analysis (GPA) map (strain component *ε*_yy_, *y*//[−1103]_η_ shown in the fast Fourier transform (FFT) pattern) reveals the strain gradient of the yellow square in (D), with a uniform region of high strain near the crack, and the corresponding HRTEM map reveals marked deformation bands in this region. The scale bar of FFT pattern is 5 nm^−1^. E) The enlarged HRTEM image of the blue square in (C) showing the deformed *θ* phase with abundant deformation bands and SFs, and the inserted FFT pattern showing the crystal rotation. The enlarged HRTEM image of the blue square in (E) shows the interface of the *θ*/matrix, and the corresponding GPA map reveals the strain component (strain component *ε*_yy_, *y*//[2 − 20]*_θ_*//[1 − 1 − 1]_Al_ shown in the FFT pattern). The scale bar of FFT pattern is 5 nm^−1^. The FFT and colored IFFT patterns demonstrate the detailed internal SFs and dislocations of the *θ* phase. The scale bar of FFT pattern is 2 nm^−1^. F) The enlarged HRTEM image of the green square in (C). GPA map (strain component *ε*_xx_) of the green square in (F) showing the strain gradient of the quasicrystalline T2 phase. The FFT and IFFT patterns reveal detailed deformation bands. The scale bar of FFT pattern is 2 nm^−1^. G) HRTEM images and FFT patterns showing deformation in the matrix, where SFs, L–C locks, distorted T2, and distorted 9R phases are observed. The scale bar of FFT patterns is 5 nm^−1^.

**FIGURE 7:**
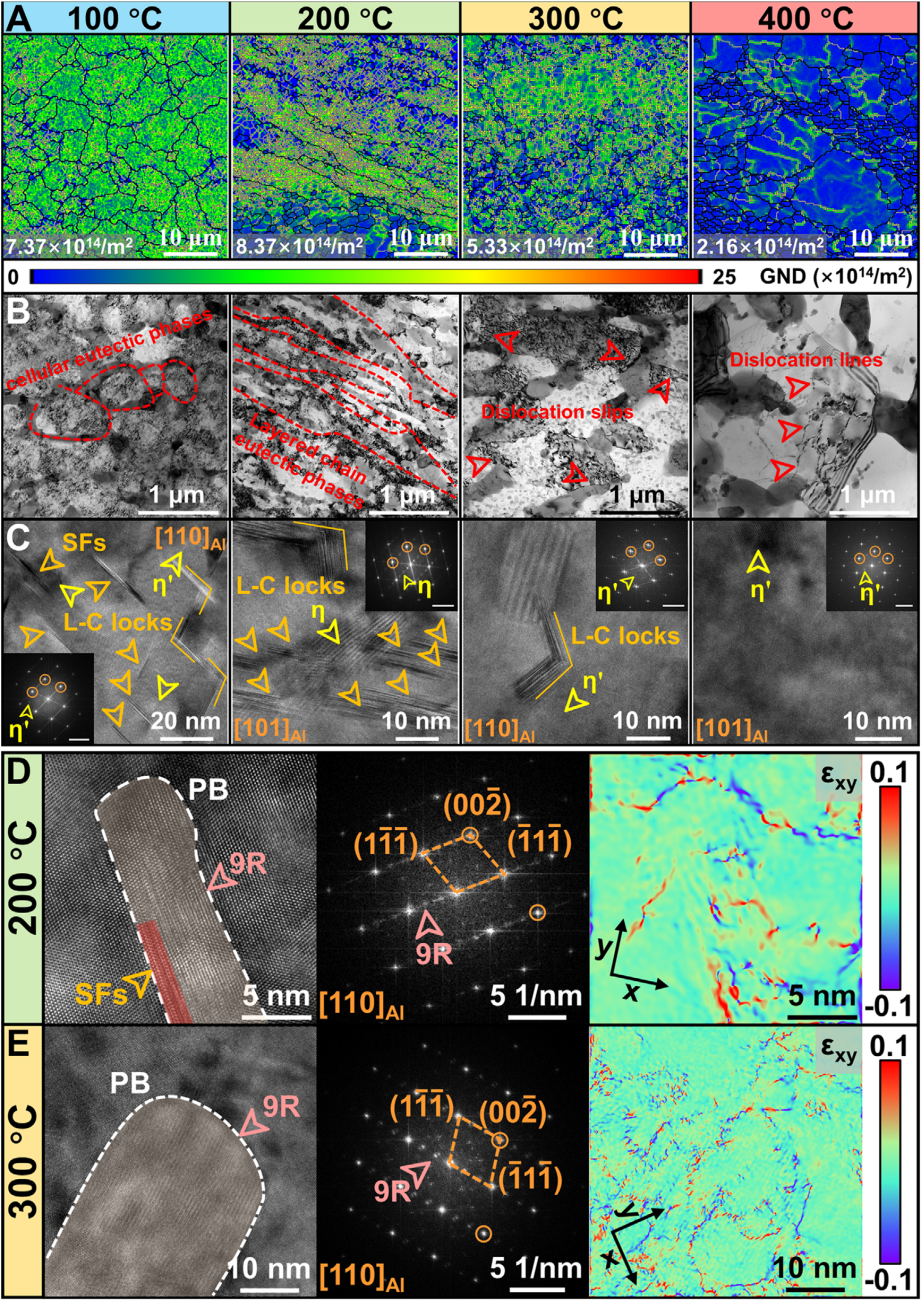
Electron backscatter diffraction (EBSD) and transmission electron microscopy (TEM) morphologies of the as‐printed Al_85_Cu_5_Li_4_Mg_3_Zn_3_ lightweight Al‐based entropy alloy (LAEA) after high‐temperature compression. A) Geometrically necessary dislocations (GND) maps after compression at different temperatures. B) Bright field (BF) images of eutectic phases and dislocation morphologies after compression at different temperatures. C) High‐resolution TEM (HRTEM) images of the α‐Al matrix after compression at different temperatures. The insets display the fast Fourier transform (FFT) patterns with a scale bar of 5 nm^−1^. D) and E) HRTEM images and FFT patterns of 9R phases after compression at 200°C and 300°C, respectively. The geometric phase analysis (GPA) maps show the strain gradient in 9R phases.

The macroscale fracture morphology at room temperature is characterized at multiple scales to provide a detailed understanding of the origin of near 20% plasticity at ultrastrong strength. Crucially, the deformation behavior provides direct evidence for how our strategy overcomes the alloy's intrinsic brittleness. Unlike brittle cleavage planes, Figure 6A shows the stepwise cracking, along with the details of the nanosized eutectic phases, such as twisted deformation features. As previously deduced, deformation proceeds through homogeneous slip propagation with layer‐by‐layer strain coordination. Notably, microcracks propagating along PBs between cellular eutectic networks and the matrix are observed perpendicular to intact GBs in Figure 6C. Microcrack nucleation facilitates local stress relaxation and creates additional dislocation pathways, enhancing intragranular strain homogeneity and delaying GB‐dominated fracture [122]. Additionally, the noncoherent precipitates scattered throughout the matrix reveal high‐density interfacial dislocations, with no signs of interface debonding or cracking. Figure 6D–F shows HRTEM images corresponding to the yellow, blue, and green boxes in Figure 6C, illustrating varying degrees of deformation ability. GPA at eutectic microcracks (Figure 6D) demonstrates HDI stress‐induced localized strain gradients, compensating for the limited deformation compatibility of polycrystalline eutectic networks revealed by APT (Figure 4). Surprisingly, interfacial dislocations and internal heterogeneous strain distribution are observed in supposedly brittle quasicrystalline phases (Figure 6F). In contrast, the isolated *θ*‐nanoprecipitate exhibits extensive deformation banding and crystal rotation (≈10.5°), transcending the traditional brittle intermetallic deformation limits (Figure 7E). Elevated HDI stress (>400 MPa) activates abundant SFs both at interface of *θ*/matrix and within precipitates. To disentangle the complex mechanical contributions within the hierarchical nanostructures, we identify that the yield strength is primarily governed by the combined effect of multilevel strengthening mechanisms. The continuous nanosized cellular eutectic networks serve as the primary load‐bearing skeleton. This dominant mechanism has been robustly validated in micropillar compression tests, where the emergence of shear bands exhibits significant hysteresis. This indicates that the quasicrystal‐reinforced networks effectively distribute stresses imposed on the matrix during initial deformation, requiring higher stresses to initiate dislocation slip. As deformation proceeds within this constrained matrix, Frank–Read (F–R) dislocation sources emit geometrically necessary dislocations (GNDs), which first create long‐range back stress opposite to the applied shear stress at the soft‐hard interfaces, activating SFs, hindering dislocation movement to coordinate heterogeneous deformation, and strengthening the matrix to accommodate strain incompatibility [123, 124]. Subsequently, the rapidly proliferating high back stress generates positive stress in the nanohard phases, promoting the activation of intrinsic plastic deformation, such as SFs and deformation bands. Precession electron diffraction (PED) mapping in Figure 7B quantifies the accumulation of GNDs at interfaces, circumventing TEM diffraction contrast artifacts from stored dislocations. Upon yielding, the core controlling factor of work hardening is attributed to the planar slip induced by high‐entropy solute effects. The high solute concentration thermodynamically inhibits dislocation cross‐slip, preventing the relaxation of back stresses. As observed in micropillar compression, deformation concentrates in a few prominent shear bands without explosive proliferation, confirming strictly confined planar slip. To coordinate strain under such high stress, the matrix must activate complex defects. Therefore, alongside the rising HDI strengthening, high‐density Lomer–Cottrell (L–C) locks formed by SF interactions across different {111} planes provide additional strengthening (Figure 7G). The L–C lock networks act as potent dislocation barriers, effectively suppressing dynamic recovery and annihilation, thereby enhancing strain hardening [125, 126]. Concurrently, the narrow spacing of the 9R phase restricts dislocation movement under high stress [127]. The 9R phase interacts with surrounding defects (e.g., dislocations and SFs), resulting in distortion and also demonstrating its ability to accommodate plastic deformation. Since the SFE is insufficient to induce deformation twinning, we still only observe discontinuous CTBs near the distorted 9R phase.

Based on the comparative EBSD and TEM analysis of the as‐printed Al_85_Cu_5_Li_4_Mg_3_Zn_3_ LAEA after high‐temperature compression (Figure 7), we reveal a distinct transition in deformation mechanisms with increasing temperature. At 100°C and 200°C, deformation is governed primarily by dislocation slip and interactions with SFs and L–C locks. Figure 7A,B shows a substantial accumulation of GNDs within grains and pronounced dislocation pile‐ups around the continuous cellular eutectic phases, indicating that these hard eutectic structures provide sustained work hardening by effectively impeding dislocation motion. A key distinction lies in the efficacy of thermal activation. At 100°C, limited thermal activation restricts dislocation cross‐slip and climb, leading to massive pile‐ups at the eutectic interfaces. Since the eutectic phases possess limited ductility, these dislocations cannot be effectively relieved. Therefore, the continuously high work hardening rate promotes storage of strain energy until a critical level is reached, triggering premature crack initiation and propagation at the eutectic/matrix interface. At 200°C, significantly enhanced thermal activation facilitates partial dislocation cross‐slip and climb, initiating dynamic recovery (DRV) [128, 129]. This process shears the continuous eutectic skeleton into fragmented layered chains. These chains bear a major portion of the strain gradient, inducing HDI stress at their interfaces with the matrix and effectively refining the mean free path of the matrix. This results in a metal matrix composite‐like hardening behavior (soft matrix reinforced with hard particles/fibers) [130, 131]. Dislocations are forced to bow out with a smaller radius (Orowan mechanism), continuously accumulating high densities of GNDs. Simultaneously, the chain‐like eutectic structure provides broader channels for dislocation glide. To maintain strain compatibility, dislocations are compelled to repeatedly bow, cross, and tangle within these confined channels, leading to a steadily increasing lattice rotation gradient [132, 133]. As the temperature remains insufficient to activate large‐scale dislocation climb or grain boundary migration, these rotations are frozen within the grains, manifesting as numerous low‐angle grain boundaries (LAGBs) inside the elongated layered grains. This enables a homogeneous distribution of the strain field, ultimately yielding macroscale high plasticity without fracture. Notably, the alloy maintains exceptional strength at this temperature (yield strength of 510 MPa, compressive strength of 800 MPa), far exceeding traditional Al alloys. Our analysis reveals that this property stems from a synergistic decoupling mechanism at high temperatures. The excellent strength is primarily attributed to the intrinsic thermal stability of the phases, as the coarsening‐resistant eutectic chains and quasicrystals maintain the structural integrity. Crucially, this is reinforced by a successful transition from “nanosized planar defect” strengthening to a “nanosized planar defect + substructure” strengthening. Specifically, the initial SFs and 9R phases serve as nucleation sites for dislocation rearrangement, promoting the formation of thermally stabilized substructures that act as fresh effective barriers. Conversely, the capacity for large deformation arises from the aforementioned dynamic recovery of the matrix, effectively releasing local stress concentrations. This allows the soft matrix to undergo extensive plastic flow around the rigid skeleton, while the newly stabilized substructures and HDI stress provided by the layered eutectic chains synergistically sustain the high strength (Figure 7C,D). A fundamental shift in the deformation mechanism occurs at 300°C, with the initiation of discontinuous dynamic recrystallization (DDRX). At this stage, thermal activation energy is sufficient to trigger recrystallization nucleation in localized regions of high stored energy [134]. However, the absence of typical dislocation‐free recrystallized grains indicates that the DDRX process is significantly suppressed (Figure 7A,B). The strength remains on a high steady‐state plateau for two reasons: (1) Intragranular, granular eutectic phases strongly pin grain boundaries, impeding the growth of new grains and boundary migration. This causes an incomplete recrystallization process, where nonrecrystallized regions are still dominated by DRV. (2) Residual SFs and coarsened 9R phases within the matrix continue to act as barriers to dislocation motion (Figure 7C,E). Deformation is thus accommodated by a combination of dislocation slip and DRV, with thermal stability conferred by the inhibition and persistent pinning of DDRX and dislocation motion by the thermally stable eutectic phases and 9R phases. Upon further increasing the temperature to 400°C, the dominant deformation mechanism transitions to continuous dynamic recrystallization (CDRX). At this temperature, the 9R phases have completely dissolved, while the eutectic phases show no significant coarsening compared to 300°C. Enhanced atomic diffusion facilitates extensive dislocation cross‐slip and climb. The stored deformation energy is now primarily released through dynamic dislocation rearrangement and the rotation/merging of subgrain boundaries, rather than via discontinuous nucleation and growth [135]. This process results in the formation of fine equiaxed grains. Herein, the eutectic phases primarily exert a Zener pinning effect, inhibiting excessive coarsening of the recrystallized grains [136].

**FIGURE 8:**
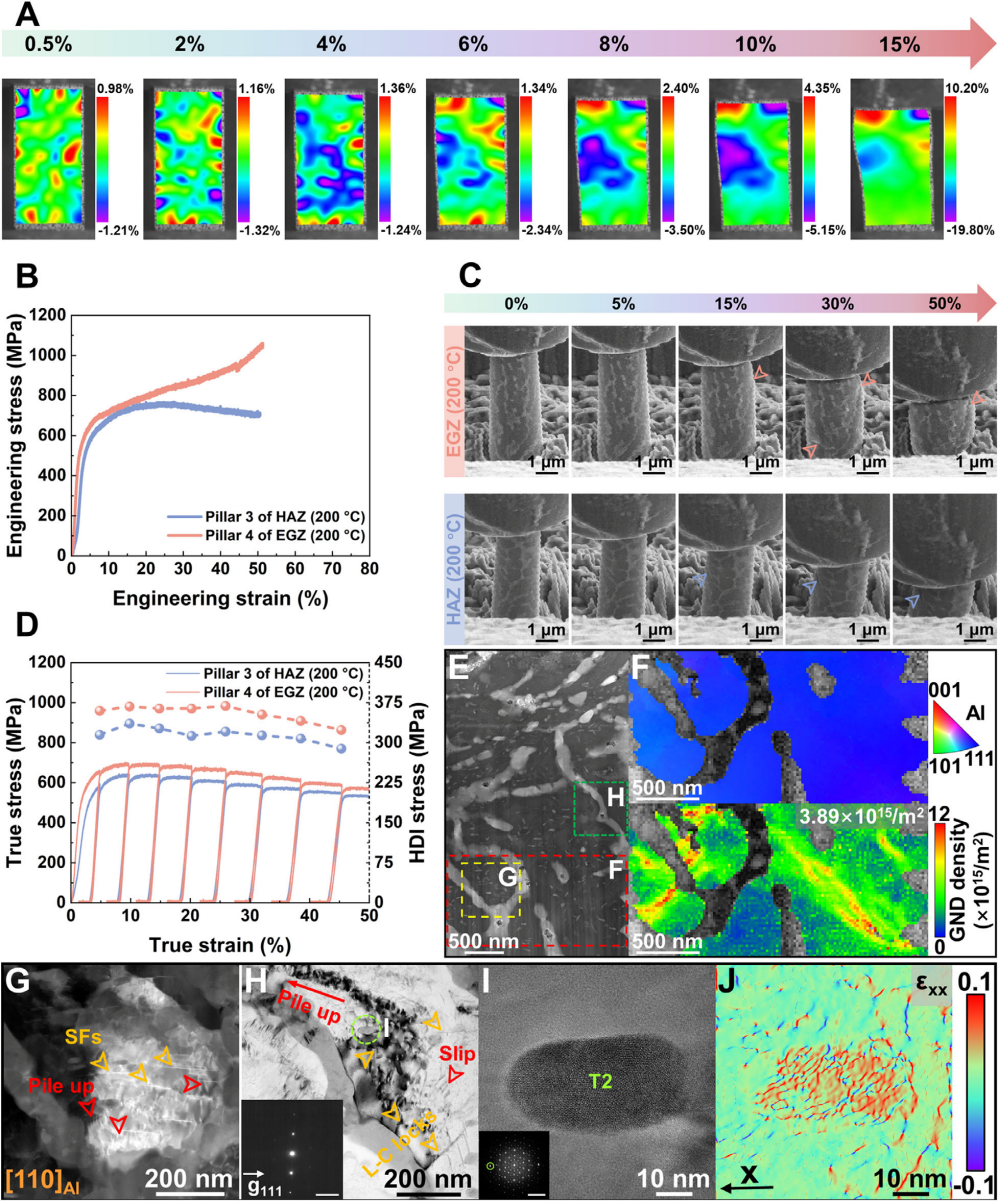
Multiscale in situ mechanical behavior of the as‐printed Al_85_Cu_5_Li_4_Mg_3_Zn_3_ lightweight Al‐based entropy alloy (LAEA) under high‐temperature compression at 200°C. A) 2D strain distribution plot showing the variation of the shear strain component (*ε*_xy_) in the measured section under compression. B) Compressive engineering stress–strain curves of the micropillars in equiaxed grain zone (EGZ) and heat‐affected zone (HAZ), respectively. C) Morphological evolution of micropillars under compression, obtained from live video screenshots. The arrows indicate the shear bands. D) Loading–unloading–reloading curves and evolution of heterogeneous deformation‐induced (HDI) stress corresponding to different true strains of the micropillars in EGZ and HAZ, respectively. E) HADDF–STEM image of post‐deformation micropillars in EGZ. F) TKD analysis of the red square in (E), with an inserted phase map and GND map. G) DF‐STEM image of the yellow square in (E) under the [110]_Al_ zone axis. H) Weak‐beam BF image of the green square in (E) under the [110]_Al_ zone axis. The insert in (H) shows the diffraction spots after construction of weak beam condition, *g* = 111, with the scale bar of 5 nm^−1^. I) and J) high‐resolution TEM (HRTEM) image and geometric phase analysis (GPA) map of the green circle in (H) showing the strain distribution in T2 phase and at its interface with the matrix. The scale bar of fast Fourier transform (FFT) pattern is 5 nm^−1^.

In situ high‐temperature macroscale compression combined with digital image correlation (DIC) and micropillar compression tests provides direct evidence for the origins of the strength and plasticity in the as‐printed Al_85_Cu_5_Li_4_Mg_3_Zn_3_ LAEA at 200°C (Figure 8). DIC results reveal that the strain field during initial deformation is not homogeneous but instead forms distinct localized bands (Figure 8A). With increasing compressive strain, these strain localized bands intensify and broaden, yet they do not evolve into macroscale shear bands that would cause premature fracture. This strain distribution behavior is fully consistent with the composite‐like deformation of soft matrix (α‐Al) reinforced with hard phases (eutectic chains). Notably, even at 15% engineering strain, the DIC shows no intense strain concentration, confirming the effectiveness of dynamic recovery (DRV). The cross‐slip and climb of dislocations alleviate strain concentrations, allowing for continuous redistribution of strain within grains via evolving substructures. This mechanism suppresses early microcrack nucleation, ultimately enabling the alloy to achieve an exceptional compressive strain of 40% without fracture. In situ high‐temperature micropillar compression, performed on pillars extracted from the EGZ and HAZ (Figure S18A, Supporting Information), further elucidates this composite‐like deformation process. A significant discrepancy in mechanical response is observed (Figure 8B): the pillar of EGZ exhibits sustained work hardening (peak strength: 1061 MPa), whereas the pillar of HAZ shows flow softening after 25% strain (peak strength: 768 MPa). This contrast highlights the extreme sensitivity of the deformation mechanism to the initial microstructure and deformation scale. The continuous hardening observed in both macroscale compression and micropillar test of EGZ aligns with the findings in Figure 7, collectively confirming that the nonequilibrium nanosized planar defects, such as SFs and 9R phases, unique to the as‐printed Al_85_Cu_5_Li_4_Mg_3_Zn_3_ LAEA are crucial for suppressing dynamic recrystallization, maintaining high work hardening, and delivering superior high‐temperature strength [137]. In contrast, the HAZ, having undergone thermal cycling and partial recovery, exhibits diminished work hardening and reaches the critical condition for localized softening more readily [138]. The post‐peak softening in the micro pillar of HAZ is attributed to highly localized, intense recovery confined within the microscale volume, a process difficult to capture in macroscale samples. This comparison underscores the high‐temperature advantage of the narrow HAZ (Figure 2D) in the as‐printed Al_85_Cu_5_Li_4_Mg_3_Zn_3_ LAEA. Furthermore, Figure 8C and Movie S1,S2 (Supporting Information) reveal that even at the nanoscale volume, pronounced strain localization (shear band) does not initiate until approximately 15% engineering strain, with few additional shear bands forming thereafter. This indicates that plastic deformation is initially accommodated cooperatively by dislocation glide and DRV throughout the volume, where constrained dislocation motion by the hard phases effectively delays the onset of strain localization [139]. The evolution of HDI stress with true strain for both micro pillars of EGZ (maximum ∼369 MPa) and HAZ (maximum ∼336 MPa), resulting from the local strain incompatibility between soft‐hard phases, demonstrates substantial and sustained HDI strengthening (Figure 8D). The origin of this mechanical behavior is clearly illustrated in Figure 8E–J: dislocations gliding in the matrix pile up significantly at the interfaces with hard phases (eutectic and quasicrystalline phases), creating strong local strain gradients. TKD analysis in Figure 8F directly visualizes the emission and accumulation of GNDs at these PB. Concurrently, the widespread presence of SFs and L–C locks within the matrix (Figure 8G,H) acts as potent short‐range barriers, further impeding dislocation motion and enhancing dislocation accumulation efficiency. Additionally, Figure 8I,J depicts the lattice strain field at the quasicrystal/matrix interface. A critical distinction from the room‐temperature behavior (Figure 6F), where strain is highly concentrated within the quasicrystal, is observed. At 200°C, GPA analysis reveals a continuous transfer of strain from the matrix into the quasicrystal interior. This key difference suggests that thermal activation at elevated temperature unfreezes the characteristic phason fluctuations of the quasicrystal, enabling it to respond to and accommodate strain transmitted from the matrix through coordinated local atomic rearrangements [140]. This remarkable quasi‐plastic accommodation ability transforms the quasicrystal from a strain concentrator at room temperature into an efficient strain transfer channel at high temperature.

### 3.3 Controllable Quasicrystal‐to‐Crystal Phase Transformation During Thermal Exposure and Its Activation of SFs

**FIGURE 9:**
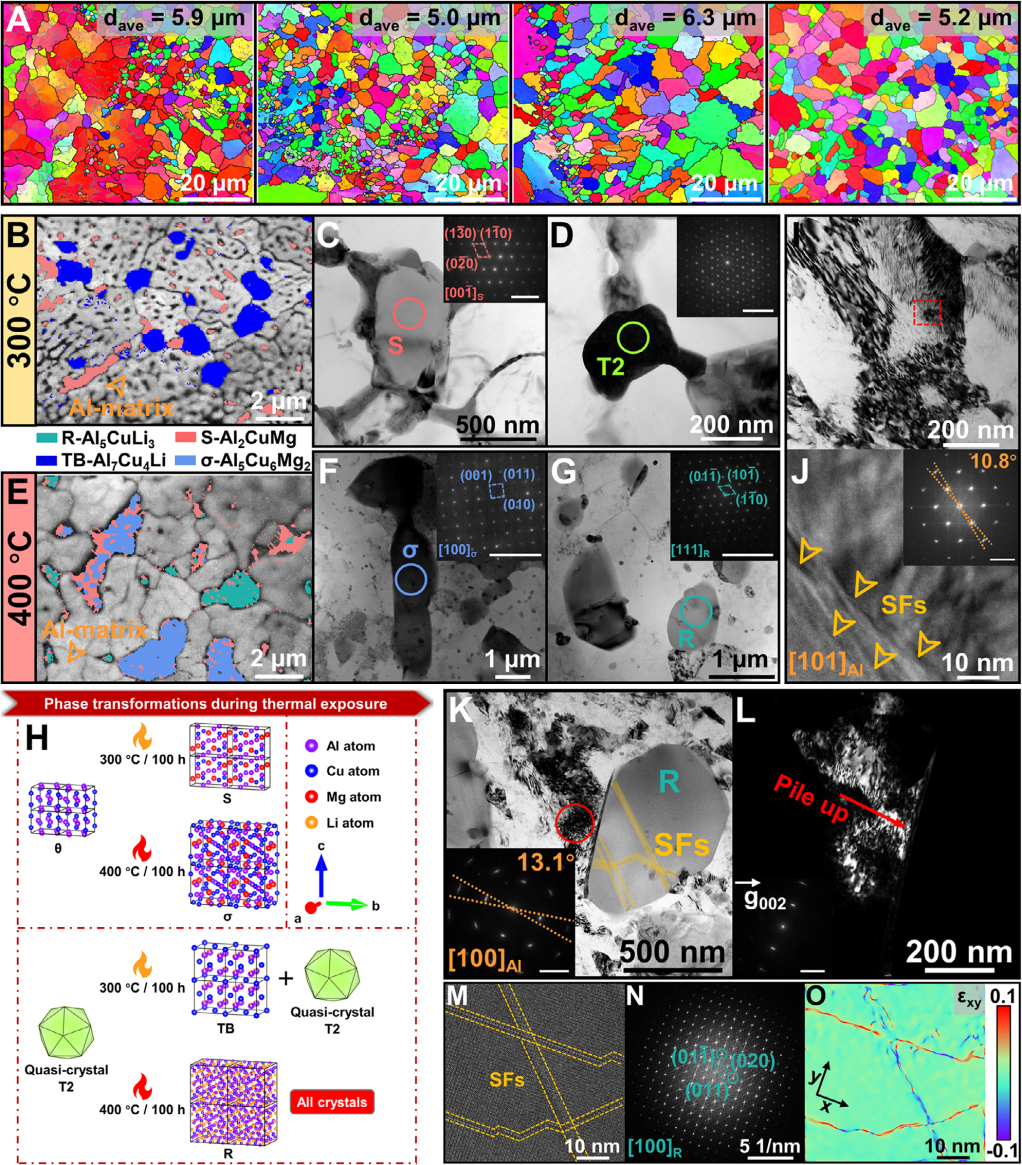
Microstructure and phase transformation of the as‐printed Al_85_Cu_5_Li_4_Mg_3_Zn_3_ lightweight Al‐based entropy alloy (LAEA) after thermal exposure. A) Inverse pole figure (IPF) maps after thermal exposure at 100°C, 200°C, 300°C, and 400°C for 100 h, respectively. B) TKD analysis showing the S phase and TB phase after thermal exposure at 300°C for 100 h, with an inserted BC+phase map. C) and D) BF images of the S phase and T2 phase. The insets display the fast Fourier transform (FFT) patterns with a scale bar of 5 nm^−1^. E) TKD analysis showing the S phase, *σ* phase, and R phase after thermal exposure at 400°C for 100 h, with an inserted BC + phase map. F) and G) Bright field (BF) images of the *σ* phase and R phase. The insets display the FFT patterns with a scale bar of 5 nm^−1^. H) Schematic diagram of phase transformations during thermal exposure at 300°C and 400°C. I) and J) BF and high‐resolution transmission electron microscopy (HRTEM) images showing SFs and lattice rotation in the matrix after room‐temperature compressive deformation after thermal exposure at 400°C. K) BF image of the R phase after room‐temperature compressive deformation after thermal exposure at 400°C showing the activated inner stacking faults (SFs). The insets display the FFT pattern and more intense lattice rotation with a scale bar of 5 nm^−1^. L) Weak‐beam DF image of the red circle in (K) under the [100]_Al_ zone axis. The insert in (L) shows the diffraction spots after construction of weak beam condition, *g* = 002, with the scale bar of 5 nm^−1^. M) to O) HRTEM image and geometric phase analysis (GPA) map of the R phase in (K) showing the crossed and stepped SFs and strain distribution in R phase. The inset displays the FFT pattern of R phase.

To probe the high‐temperature microstructural stability and identify routes for property enhancement of the alloy, we have subjected it to systematic thermal exposure treatments. As shown in Figure 9A, the average effective grain diameter (*d*_a__v__e_) remains stable compared to the as‐printed state after 100 h at temperatures up to 400°C, although the fine‐grained zones progressively diminish. Exposures at 100°C and 200°C induce only the precipitation and coarsening of intragranular η′/η phases (Figures S19A–C and S20, Supporting Information), without triggering bulk elemental diffusion or other phase transformations. The nanosized cellular eutectic networks, while stable at 300°C with only minor refinement, undergo complete decomposition into isolated particles at 400°C. Our analysis further reveals the temperature‐dependent phase transformations in the *θ* phase. EDS maps indicate the enrichment of Mg and Zn at 300°C and 400°C, respectively (Figure S19D,E, Supporting Information). Consistent with this, TKD and TEM analyses in Figure 9B–G identify the transformation products: the *θ* phase converts to S phase‐Al_2_CuMg at 300°C and to *σ* phase‐Al_5_Cu_6_Mg_2_ at 400°C, governed by the extent of Mg diffusion and eutectic network breakdown. However, Zn plays no active role in these transformations. A remarkable concomitant increase in room‐temperature strength and ductility emerges after the thermal exposure at 400°C (Figure 5A). We attribute this anomalous synergy to a complete and controlled phase transformation in which the metastable quasicrystalline T2 phase is fully converted into the stable body‐centered cubic R phase. According to the calculated equilibrium phase diagram (Figure S7A, Supporting Information), the T2 phase is thermodynamically unstable below 450°C, where the R phase prevails as the stable phase. Therefore, thermal exposure at 400°C provides the necessary activation energy to overcome the kinetic barrier, driving the metastable T2 quasicrystals (frozen by SLM) to return to their thermodynamic equilibrium state (crystalline R phase). This transformation fundamentally redefines the alloy's strengthening and plasticizing mechanisms. After thermal exposure at 300°C, partial transformation of the T2 phase to the face‐centered cubic TB phase‐Al_7_Cu_4_Li occurs. The TB phase/matrix coherence enhances plasticity but contributes negligibly to strength, which remains primarily dependent on the residual T2 phase. In stark contrast, the thermal exposure at 400°C yields a distinct microstructure. While the matrix retains its ability for strain accommodation through prolific SFs formation and lattice rotations (Figure 9I,J) [141], the T2 phase is now entirely replaced by the R phase. Crucially, this phase transformation unlocks a unique intra‐phase deformation mode within the hierarchical nanostructures. We identify two cooperative mechanisms enabling the superior strength‐ductility match. First, the R‐phase generates a potent HDI strengthening effect, evidenced by pronounced dislocation pile‐ups at its boundaries (Figure 9K,L). Second, and more critically, unlike traditional brittle intermetallics, the formed R‐phase itself possesses intrinsic deformability. Under compression, it activates a high density of crossed or stepped SFs within its own lattice (Figure 9M–O). These internal SFs serve a dual purpose: they act as nanoscale strengthening barriers while also operating as an intrinsic strain‐accommodation system via their nucleation and limited glide [142]. This additional plastic reserve effectively coordinates the strain incompatibility between the matrix and the skeleton, delaying localized shear instability. Consequently, by strategically employing thermal exposure to drive the complete T2‐to‐R transformation, we harness the low SFE to activate carriers of plastic deformation not only in the matrix but also within the strengthening phase itself. This creates a percolating, synergistic network for strain tolerance, achieving a previously inaccessible balance of properties.

## 4 Conclusion

In summary, this work establishes a novel data‐driven design paradigm for high‐performance LAEAs tailored for additive manufacturing. By leveraging the synergy of QML and high‐throughput thermodynamic calculations, we have successfully navigated the vast compositional space to identify the Al_85_Cu_5_Li_4_Mg_3_Zn_3_ LAEA, effectively resolving the conflict between high solute content and printability. The central design principle of this work is the eutectic‐stabilized entropy strategy, which strategically employs high‐entropy solute additions to induce the formation of extensive nanosized cellular eutectic networks at terminal solidification. This mechanism not only accommodates thermal shrinkage to eliminate hot cracking but also establishes a rigid architectural skeleton. Crucially, the nonequilibrium nature of additive manufacturing further transforms these eutectic structures into deformable hierarchical nanostructures containing unique nanosized planar defects. These features activate synergistic deformation mechanisms that not only counteract the intrinsic compressive brittleness of this alloy class, leading to an unprecedented combination of GPa‐grade strength and plasticity, but are also responsible for its exceptional heat resistance. Furthermore, a controllable quasicrystal‐to‐crystal phase transformation unlocks a novel pathway to tune strength and ductility by activating a new internal deformation mode. Beyond this specific alloy, this framework serves as a generalized blueprint applicable to other complex material systems, implying that traditionally unprintable high‐alloy compositions can be rendered processable through such precise eutectic engineering. However, the same interconnected hard skeleton that ensures printability and thermal stability currently imposes a constraint on tensile ductility compared to the extensive compressive plasticity. Consequently, the future trajectory of this work lies in the generalization of this data‐driven framework to broader LAEAs. By applying the established screening protocols to unexplored compositional systems, future work aims to discover novel candidates which retain eutectic‐stabilized printability but feature intrinsically more ductile matrices. This holistic understanding linking computational design, advanced manufacturing, and a multifaceted mechanical response establishes a powerful paradigm for creating next‐generation high‐performance lightweight alloys.

## Author Contributions

Conceptualization: E.M.W., H.B.W., S.L.S., and J.Z.J. Methodology: E.M.W., C.D., H.B.W. Investigation: E.M.W., C.D., D.Y.Z., and C.J.X. Visualization: E.M.W., C.D., and D.Y.Z. Funding acquisition: H.B.W., J.Z.J. Supervision: H.B.W., S.L.S., and J.Z.J. Writing – original draft: E.M.W. Writing – review and editing: H.B.W., S.L.S., and J.Z.J.

## Conflicts of Interest

The authors declare no conflicts of interest.

## Supporting information




**Supporting file 1**: advs74004‐sup‐0001‐SuppMat.docx.

**Supporting file 2**: advs74004‐sup‐0002‐MovieS1.mp4.

**Supporting file 3**: advs74004‐sup‐0003‐MovieS2.mp4.

**Supporting file 4**: advs74004‐sup‐0004‐DataSet.zip.

## Data Availability

The data that support the findings of this study are available in the supplementary material of this article.
