# Supplementary material for: Data‐Driven Design and Fabrication of Heat‐Resistant, Ultrastrong, Lightweight Aluminum‐Based Entropy Alloy by Additive Manufacturing
Source: Adv Sci (Weinh). 2026 Jan 21;13(19):e22817. doi: 10.1002/advs.202522817 (PMC13045303; doi:10.1002/advs.202522817)
Supplement: Supplementary file 1 — Supporting file 1: advs74004‐sup‐0001‐SuppMat.docx. [file ADVS-13-e22817-s003.docx]

**Supporting information**

**Data-Driven Design and Fabrication of Heat-Resistant, Ultrastrong, Lightweight Aluminum-Based Entropy Alloy by Additive Manufacturing**

Enmao Wang^a,b^, Chao Ding^a^, Danyang Zhou^a^, Chenjin Xu^a^, Swee Leong Sing^b,^*, Jianzhong Jiang^c,^*, and Huibin Wu^a,^*

E. M. Wang, C. Ding, D. Y. Zhou, C. J. Xu, H. B. Wu

^a^ Collaborative Innovation Center of Steel Technology, University of Science and Technology Beijing, Beijing 100083, China

E-mail: [whbustb@163.com](mailto:whbustb@163.com)

E. M. Wang, S. L. Sing

^b^ Department of Mechanical Engineering, National University of Singapore, Singapore 117575, Singapore

E-mail: [sweeleong.sing@nus.edu.sg](mailto:sweeleong.sing@nus.edu.sg)

J. Z. Jiang

^c^ Key Laboratory of Silicon-based Materials, The Ministry of Education, Key Laboratory of Automotive Glass of Fujian, Smart Automotive Glass Engineering Research Center of Fujian, and School of Materials Science and Engineering, Fuyao University of Science and Technology, Fuzhou, Fujian, 350109, China

E-mail: [jiangjz@fyust.edu.cn](mailto:jiangjz@fyust.edu.cn)

1. Experimental Section

1.1. QML and HTC

The open-source HEAs dataset was curated and preprocessed, categorizing samples into three phase classes: solid solution, solid solution + intermetallic, and amorphous. Sixteen key features (electronegativity, atomic radius, mixing entropy, mixing enthalpy, etc.) were selected and standardized to ensure dimensional consistency. Synthetic Minority Oversampling Technique (SMOTE) was applied to balance phase class distributions, followed by partitioning the dataset into a 70% training set, 10% validation set, and 20% test set. After preprocessing, a traditional XGBoost model was implemented in PyCharm with hyperparameter optimization via cross-validation and the Optuna framework, achieving peak performance on the validation set. This XGBoost model served as the classical benchmark for quantifying the contributions of quantum enhancement.

A quantum-XGBoost hybrid model was developed to capture non-linear high-order feature interactions. The real superconducting quantum computer (Xiaohong) of the Quantum Computing Cloud Platform of the Chinese Academy of Sciences was accessed from a local computer via PyCharm to execute quantum computations. The quantum circuit contains 16 topologically connected qubits. This architecture minimizes encoding complexity, avoids feature compression and the overhead of multiple uploads, while preserving the complex interactions and entanglement between features. The one-to-one feature-to-qubit mapping strategy enhances circuit fidelity and interpretability by maintaining direct feature correspondence throughout the quantum operations.^[1]^ Qubits were initialized to |0› ground states, with feature encoding achieved through Bloch sphere rotation as shown in Figure S21. The quantum circuit integrated with RZ (Rotation about z-axis), RY (Rotation about y-axis), √X (Half-not gate), √X^†^ (Conjugated transpose half-not gate), H (Hadamard gate), Control-Z, and measurement gates, each serving specific roles in feature encoding, quantum state transformation, quantum entanglement, quantum superposition, and quantum measurement of classical data, thereby enhancing the ability to capture non-linear feature interactions.^[2,3]^ Specifically, two primary rotation gates, RY and RZ, were employed to encode feature values into quantum states. Mathematically, quantum state encoding is expressed by the following equation:^[3,4]^

$$\begin{aligned} \text{|}\text{ψ}_{\text{i}}\text{› = }\text{R}_{\text{Z}}\text{(}\text{ϕ}_{\text{i}}\text{)}\text{R}_{\text{Y}}\text{(}\text{θ}_{\text{i}}\text{)|0›}\#\text{(}\text{1}\text{)} \end{aligned}$$

where *ϕ*_i_ and *θ*_i_ represent the rotation angles mapped from the feature value, constrained within the range of [-Π, Π]. The RZ gate modified the phase angle of the quantum state on the Bloch sphere, while the RY gate adjusted the rotation along the y-axis, primarily affecting the amplitude. The √X and √X^┼^ gates were square-root variants of the Pauli-X gate, rotating the quantum state from |0› to |1›, which was not a complete flip, rather it produced an intermediate state. The √X gate smoothly rotated the quantum state from its initial state into a superposition, while the √X^┼^ gate performed the inverse operation. This symmetry made the circuit more flexible in adjusting the evolution path of the quantum state. Additionally, after the CZ gate was applied, the quantum state transitioned into a multi-body entangled state. The subsequent H gate redistributed the entangled states into a uniform superposition, enhancing complexity and reducing the concentration of information in specific qubits. Finally, the measurement gate projected the quantum state onto classical bits, reading the state of each qubit. Each measurement produced a 16-bit binary string (composed of 0/1), which was mapped back to the classical feature space to serve as input for the XGBoost model after statistical analysis and probability distribution processing. The hybrid model was trained to minimize the loss function, with key XGBoost hyperparameters optimized and fed back in real-time via variational quantum circuits (Figure S22) for final training and validation.

HTC was performed using PyCharm, and the Solidification Cracking Model, an open-source software for MATLAB developed by Lv et al,^[5]^ was utilized. The theoretical density, thermal cracking sensitivity, solidification of the solid bridge, and evaporation rate were sequentially calculated for 1815 alloys. The theoretical density was obtained by the weighted average of the atomic density and fraction of each element using the following equation:^[6]^

$$\begin{aligned} \text{ρ}\text{ = }\frac{\sum_{\text{i = 1}}^{\text{n}} \text{C}_{\text{i}}\text{ρ}_{\text{i}}}{\sum_{\text{i = 1}}^{\text{n}} \text{C}_{\text{i}}}\#\text{(}\text{2}\text{)} \end{aligned}$$

where *C*_i_ is the atomic fraction of element, and *ρ*_i_ is the density of element (g/cm^3^). The calculations for hot cracking susceptibility and solidification of the solid bridge were performed using the Solidification Cracking Model software, which is based on the competition between thermal stress accumulation and the development of solid bridge strength. By incorporating relevant thermodynamic and mechanical parameters, the software simulates the hot cracking tendency of binary alloys under high cooling rates and complex thermal gradients in AM, as well as the formation of a solid bridge during solidification. The required key parameters are listed in Table S1. The evaporation rates were calculated based on the vapor pressures of Li, Mg, and Zn, which are prone to evaporation, and the melting pool temperature. The vapor pressure of each element was calculated using the following Antoine equation:^[7]^

$$\begin{aligned} \text{ln(}\text{P}_{\text{i}}\text{) = A - }\frac{\text{B}}{\text{T}}\text{ + Clog}\text{T}\text{ +D}\text{T}\text{ ×}\text{ }\text{10}^{\text{-3}}\#\text{(}\text{3}\text{)} \end{aligned}$$

where *P*_i_ is the vapor pressure (Pa), A, B, C, and D are specific constants for the elements, and *T* is the temperature (K). Then, the Knudsen-Langmuir equation was used to calculate the evaporation rate for each element:^[8,9]^

$$\begin{aligned} \text{V}_{\text{i}}\text{ = }\frac{\text{α}\text{P}_{\text{i}}}{\sqrt{\text{2Π}\text{M}\text{R}\text{T}}}\#\text{(}\text{4}\text{)} \end{aligned}$$

where *V*_i_ is the evaporation rate (kg/(m^2^·s)), *α* is the evaporation coefficient (approximated as the atomic fraction), *M* is the molar mass (kg/mol), and R is the ideal gas constant (8.314 J/(mol·K)). The average evaporation rates of each element were calculated over the temperature range of 1773 K to 2273 K to simulate evaporation behavior at different temperatures during the SLM process. Finally, these average values were summed to obtain the total theoretical evaporation rate of the alloy.

1.2. Computational simulation

To simulate the solidification process, an idealized model was adopted. The initial radius of the melting pool was set, with the assumption that its shape remains unchanged throughout the process. Inside the melting pool, a liquid alloy was present, while the surrounding area was solid. Cellular Automata (CA) model: To better align with the actual nucleation process, the quasi-continuous nucleation model proposed by RAPPAZ et al.^[10]^ and THEVOZ et al.^[11]^ was adopted. The model uses a Gaussian distribution function to describe the relationship between nucleation density and undercooling:^[12]^

$$\begin{aligned} \text{n}\text{(Δ}\text{T}\text{) = }\int_{\text{0}}^{\text{Δ}\text{T}} \frac{\text{d}\text{n}}{\text{d(Δ}\text{T}\text{)}}\text{d(Δ}\text{T}\text{) = }\frac{\text{n}_{\text{max}}}{\text{Δ}\text{T}_{\text{σ}}\sqrt{\text{2Π}}}\text{ }\int_{\text{0}}^{\text{Δ}\text{T}} \text{exp}\left[ \text{-}\frac{{\text{(Δ}\text{T}\text{-Δ}\text{T}_{\text{n}}\text{)}}^{\text{2}}}{\text{2}{\text{(Δ}\text{T}_{\text{σ}}\text{)}}^{\text{2}}} \right]\text{d(Δ}\text{T}\text{)}\#\text{(5)} \end{aligned}$$

where *n* is the nucleation density (mm^-3^), Δ*T* is the undercooling (K), Δ*T*_n_ is the nucleation undercooling (K), and Δ*T*_σ_ is the standard deviation of nucleation distribution. In this model, undercooling plays a critical role in determining the nucleation potential of each cell. The nucleation process at each time step is determined based on the local undercooling and nucleation density values. When the undercooling exceeded the critical nucleation condition, nucleation occured.

A diffusion interface model for dendritic growth was employed to simulate solute concentration distribution, where solute concentration gradients drove the solidification process. The improved CA method, combined with the finite difference (FD) method, was used to solve the concentration and temperature fields. Specifically, the finite difference method was used to solve the heat transfer and solute diffusion equations, which govern the temperature^[13]^ and concentration distribution^[14]^ across the simulation area:

$$\begin{aligned} \text{ρ}\text{c}_{\text{p}}\frac{\text{∂}\text{T}}{\text{∂}\text{t}}\text{ = }\text{∇}\text{ · }\left( \text{λ}\text{∇}\text{T} \right)\text{ + }\text{Q}\#\text{(6)} \end{aligned}$$

where *ρ* is the density (g/cm^3^), *c*_p_ is the specific heat capacity (J/(kg·K)), *λ* is the thermal conductivity (W/(m·K)), and *Q* is the volumetric heat input from laser beam (W).

$$\begin{aligned} \text{D}\text{(}\text{T}\text{) = }\text{D}_{\text{0}}\text{ exp}\left( \text{-}\frac{\text{Q}_{\text{g}}}{\text{R}\text{T}} \right)\#\text{(7)} \end{aligned}$$

where *D* is the diffusion coefficient (m^2^/s), *D*_0_ is the pre-exponential coefficient (m^2^/s), and *Q*_g_ is the activation energy (KJ/mol). This provided a more accurate representation of the thermal and solute behavior during the solidification process. The simulation area was divided into 200×110 mm rectangular grid units, and the physical parameters used in the model are provided in Table S2. The model was implemented in MATLAB, and the growth morphology of dendrites, including equiaxed and columnar crystals, was simulated and compared with experimental data to validate the model's accuracy.

1.3. Materials processing

Ingots and powders were prepared using the actual composition (wt.%) shown in Table S3. Al, Li, Mg, Cu, and Zn (with purities >99.9%) were selected to melt the Al_85_Cu_5_Li_4_Mg_3_Zn_3_ alloy. The arc-melting technique was employed for casting in an argon atmosphere, with the materials being remelted three times after being flipped to ensure compositional homogeneity. The gas-atomized pre-alloyed powders were supplied by Liaoning Guanda New Material Technology Co., Ltd., with a particle size distribution of ~15–53 µm. The as-printed alloys were fabricated using the SLM machine LiM-X260A, equipped with an IPG 500 W fiber laser source. The printing process was conducted under a protective argon atmosphere. The optimized processing parameters were as follows: laser power ~220 W, scanning speed ~600 mm/s, scanning spacing ~100 µm, layer thickness ~30 µm, and preheating temperature ~150 °C. An island scanning strategy was adopted, with an island size of 5×5 mm and an interlayer rotation of 67°.

1.4. Microstructural characterization

The composition of the powders and samples was measured using an inductively coupled plasma optical emission spectrometer (ICP-OES). After vacuum drying, the powders were used for TOF-SIMS test to obtain the chemical composition distribution on the particle surface, with particular focus on the light element Li. An ION-TOF GmbH instrument was used, featuring a 30 kV pulsed Bi_3_^+^ primary ion beam, a grating size of 150×150 μm, and a mass detection range of 2–1000 μ, under high mass resolution mode. The bulk samples were ground, mechanically polished, and then etched with Keller's reagent. The microstructure was examined using a Leica DM4M OM and a Zeiss Gemini 500 field emission SEM with EDS analysis. The samples electropolished with a 10 vol.% nitric acid alcohol solution were used for phase analysis (CuKα, 2θ range of 10°–90°, step size of 0.02°) and macrotexture analysis (CuKα, tilt angle range of 15°–90° with a 360° rotation, step size of 5°) on a Bruker D8 ADVANCE X-ray diffractometer. The samples milled by an argon ion beam were used for EBSD characterization, with an EBSD detector integrated into the Zeiss Gemini 500, and a step size of 0.2 μm. The EBSD data were processed using AztecCrystal software. The nano-sized heterostructure and deformation features were characterized using an FEI Talos F200X TEM equipped with a HAADF detector and a Super-X EDS spectrometer. TEM samples were prepared via ion thinning, and data post-processing was performed using DigitalMicrograph software. PED is a TEM-based technique, with sample preparation identical to that for TEM. Using the rapid precession electron beam of a TEM equipped with a NanoMEGAS ASTAR platform (precession angle of 0.3°, step size of 10 nm), the acquired PED patterns were rapidly matched with standard diffraction spectra by ASTAR MapViewer. APT tips were prepared using an FEI Scios 2 FIB/SEM. The transmission Kikuchi diffraction (TKD) integrated into the SEM was used to analyze the phases in the alloy after thermal exposure. APT data were collected in laser mode at 50 K using a CAMECA LEAP 5000XR instrument. A pulse fraction of 20%, a pulse rate of 200 kHz, and a laser energy of 60 pJ were applied to ensure complete field ion evaporation. The AP Suite 6.3 software was used for 3D data reconstruction and analysis.

1.5. Density and mechanical testing

The as-printed bulk, with a volume of 1 cm³, was used to measure the true density. The measurement was carried out using helium displacement in an Ultra PYC 1200e fully automatic true density analyzer, with 5 cyclic tests. μ-CT analysis of a cylinder with dimensions of Φ1×1 mm was performed using a Zeiss XRADIA 620 VERSA high-resolution 3D X-ray microscope. The nominal resolution was set to a voxel size of 500 nm, with a voltage of 80 kV, power of 10 W, and exposure time of 0.25 s.

Compression tests were conducted on a SHIMADZU AGS-X 100KN electronic universal testing machine. The strain was measured using an RVX-112B video extensometer, with a strain rate of 5×10^-^⁴ s^-^¹. The compression samples were prepared with dimensions of Φ3×5 mm, while the DIC sample was machined into a cuboid with dimensions of 4×4×8 mm. All mechanical tests were repeated three times to ensure accuracy. The micropillar samples, with dimensions of Φ2×4 μm (taper <2°), were prepared using FIB. In situ micropillar compression tests were performed at room temperature and high temperature within an SEM using the Hysitron PI85 nano-mechanical testing system, operating in displacement control mode at a rate of 5 nm/s.

2. Figures and Tables


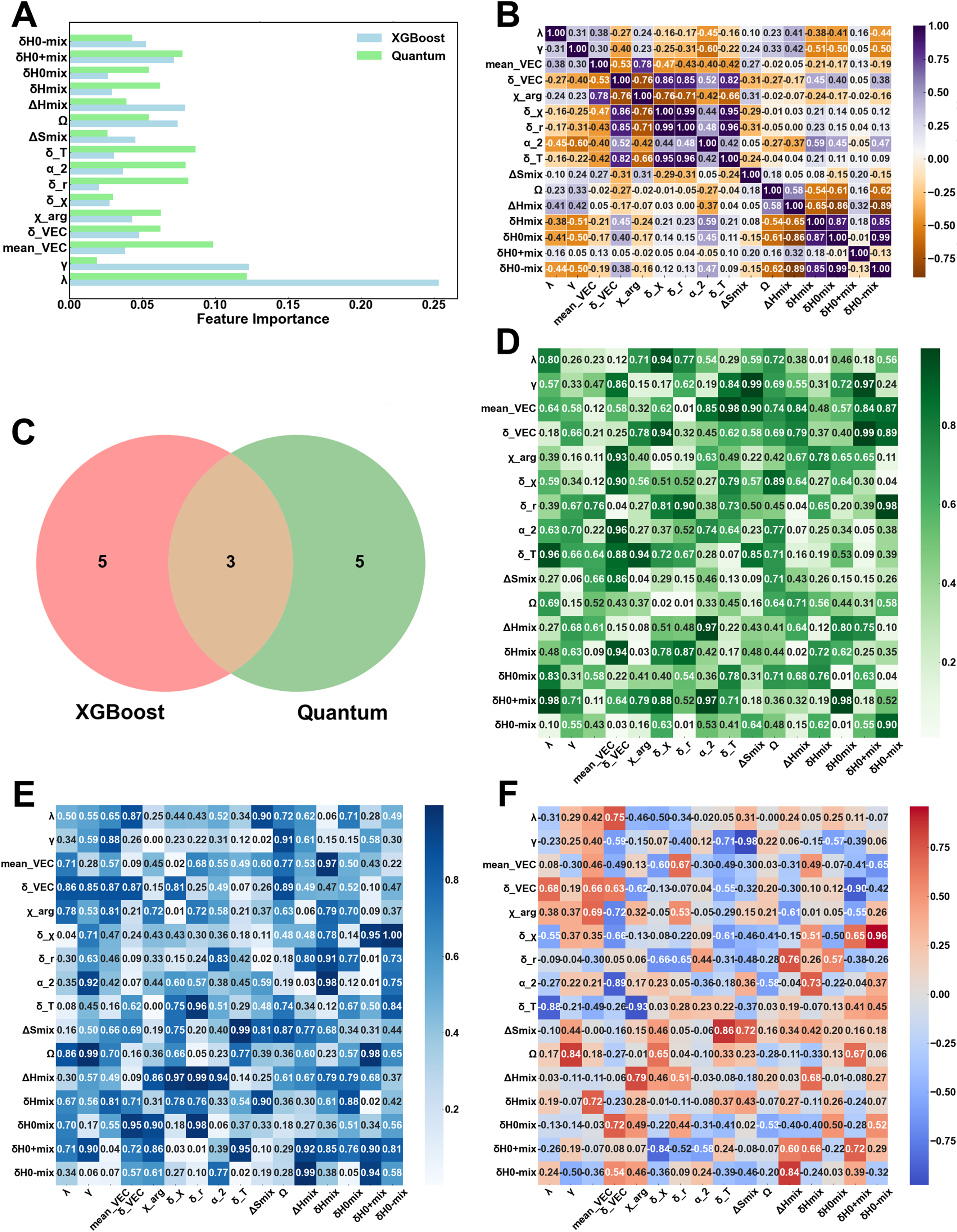


**Figure S1.** Comparison of XGBoost and quantum computation for feature recognition. A) Importance ranking of 16 features by XGBoost and quantum computation, respectively. B) Pearson correlation matrix of the features. C) Comparison of XGBoost and quantum computation for selecting the top eight most important feature subsets. D) Feature interaction matrix of XGBoost. E) Feature interaction matrix of quantum computation. F) Differences between quantum computation and XGBoost in feature interaction. The matrix demonstrates the superior recognition of non-linear higher-order interactions among different features by quantum computation.


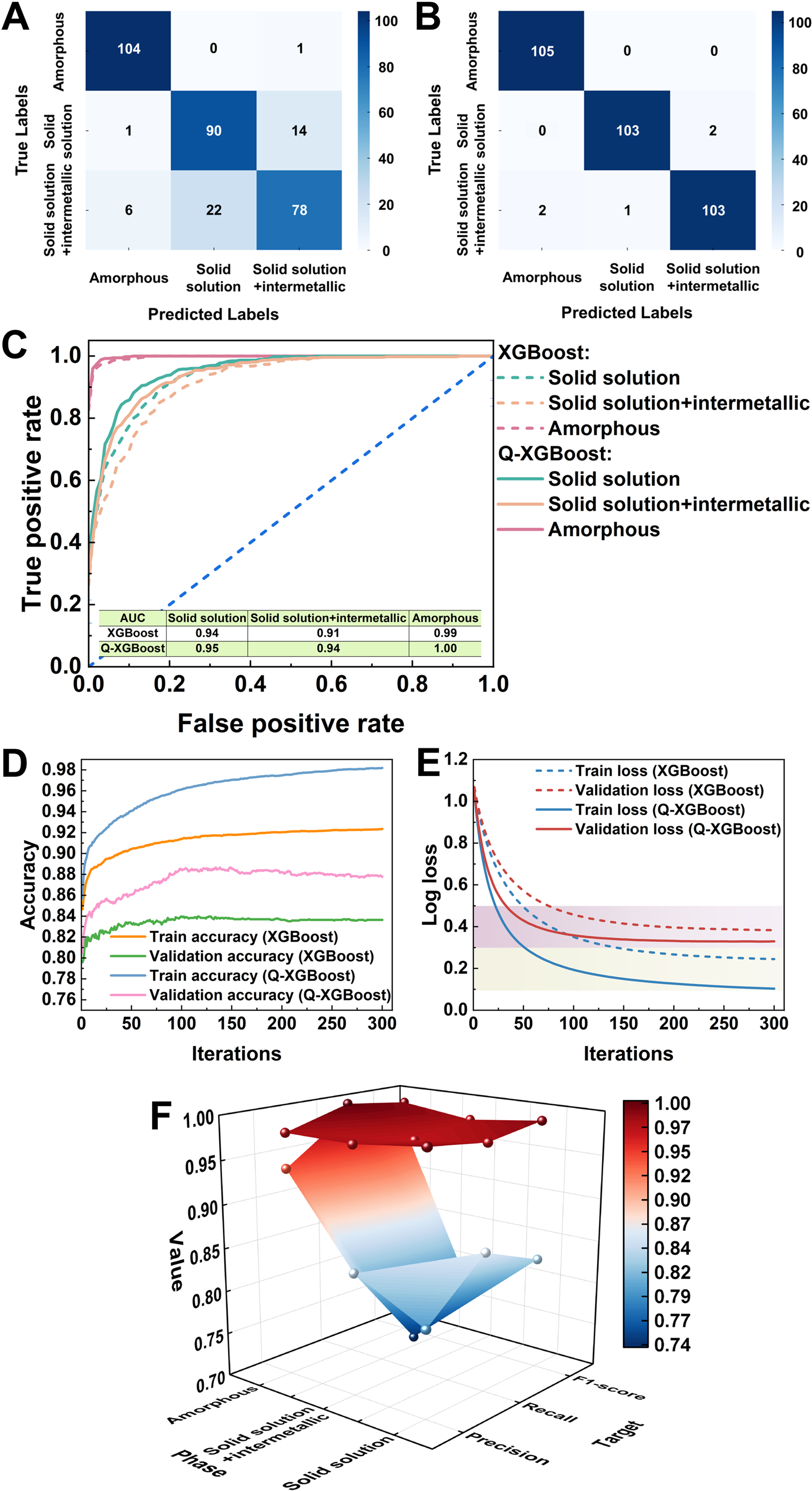


**Figure S2.** Comparison of training results and prediction accuracy of XGBoost and Q-XGBoost models. A) and B) Confusion matrix showing the prediction accuracy of XGBoost and Q-XGBoost for each class. C) ROC curves for each class across 10-folds of XGBoost (dashed lines) and Q-XGBoost (solid lines), with the inserted table showing AUC values. D) Average accuracy curves across 10-folds for the respective training and validation sets of XGBoost and Q-XGBoost. E) Average log loss curves across 10-folds for the respective training and validation sets of XGBoost (dashed lines) and Q-XGBoost (solid lines). F) 3D visualization showing the prediction performance (precision, Recall, and F1-score) of XGBoost and Q-XGBoost for each class.


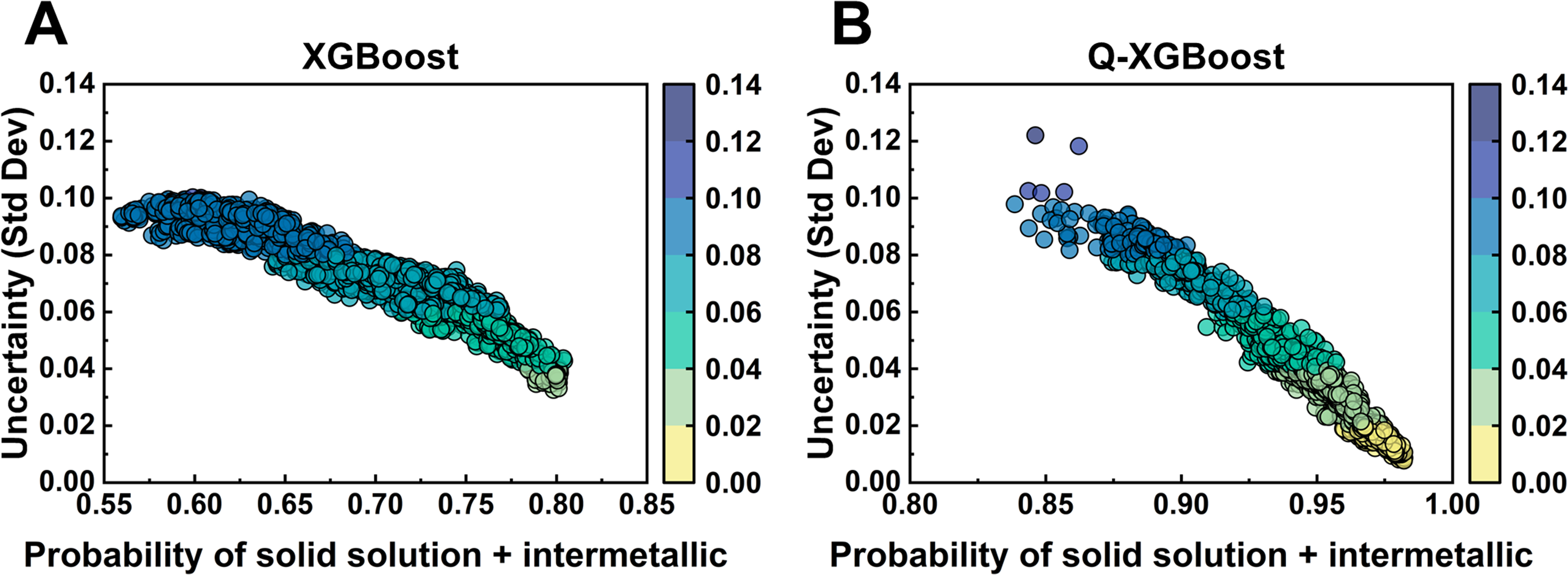


**Figure S3.** Uncertainty analysis of probability predictions of solid solution + intermetallic using A) XGBoost and B) Q-XGBoost Models.


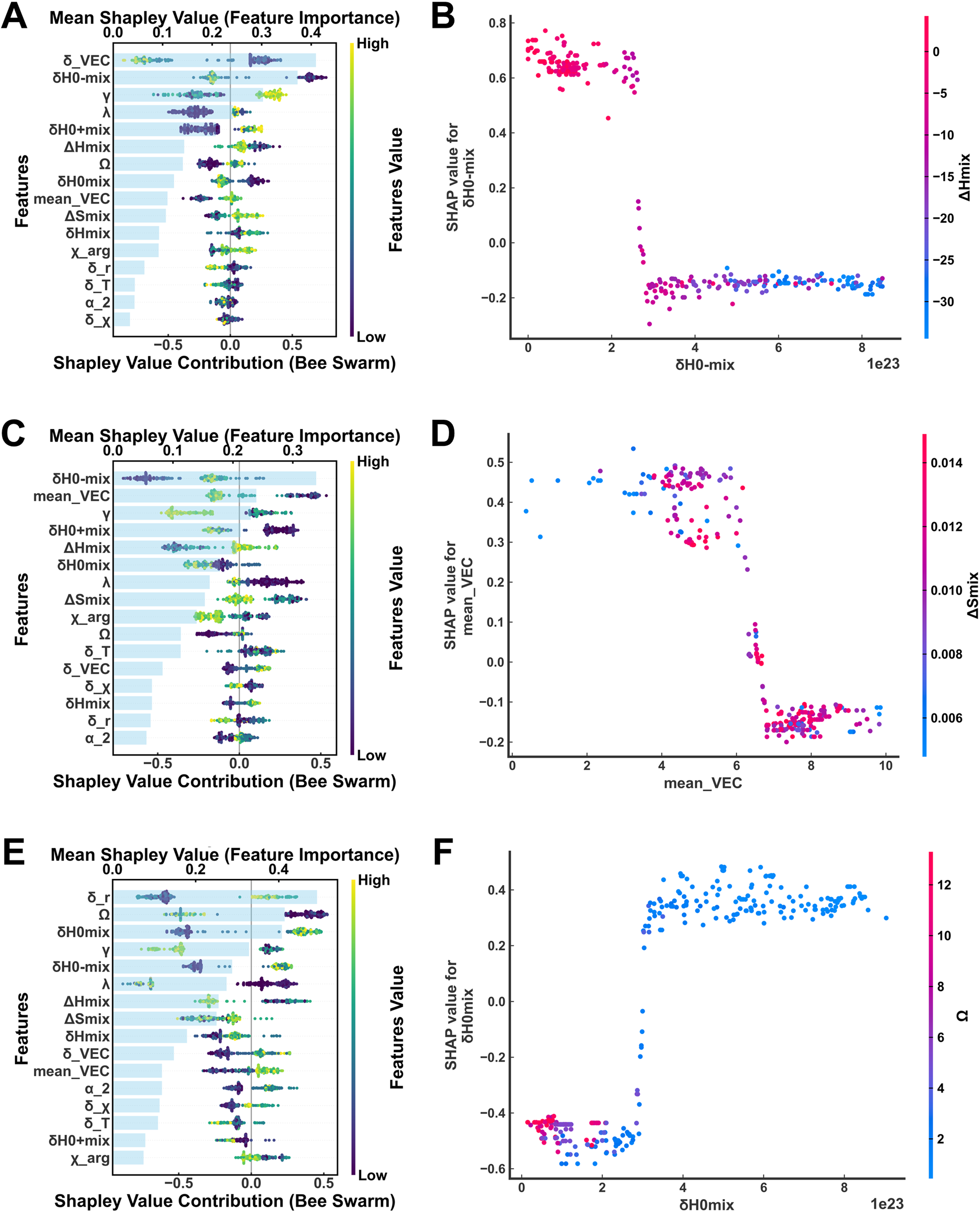


**Figure S4.** SHAP analysis to explain the optimized Q-XGBoost model. SHAP summary plots for class A) solid solution, C) solid solution + intermetallic, and E) amorphous, showing the feature importance ranking calculated based on the mean Shapley value and the bee swarm plot based on the Shapley value contribution. SHAP dependence plots for class B) solid solution, D) solid solution + intermetallic, and F) amorphous, showing the correlation between two positive important features (Shapley value contribution >0).


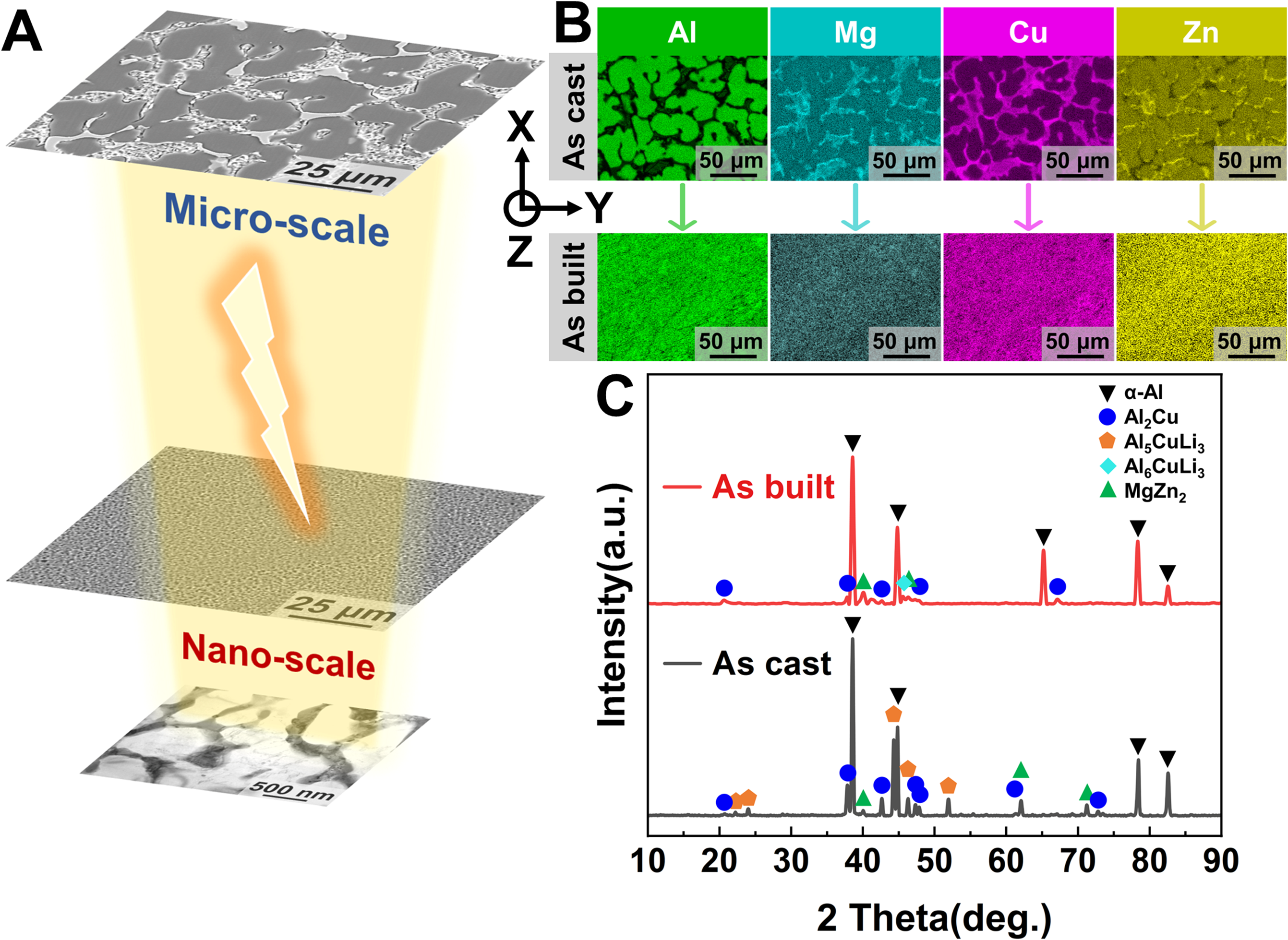


**Figure S5.** Comparison of microstructures between as-cast and as-printed Al_85_Cu_5_Li_4_Mg_3_Zn_3_ LAEAs. A) Schematic structural evolution of the as-cast and as-printed alloys from micro-scale to nano-scale. B) Energy dispersive spectroscopy (EDS) composition maps of various elements in the as-cast and as-printed alloys. C) X-ray diffraction (XRD) patterns of the as-cast and as-printed alloys.


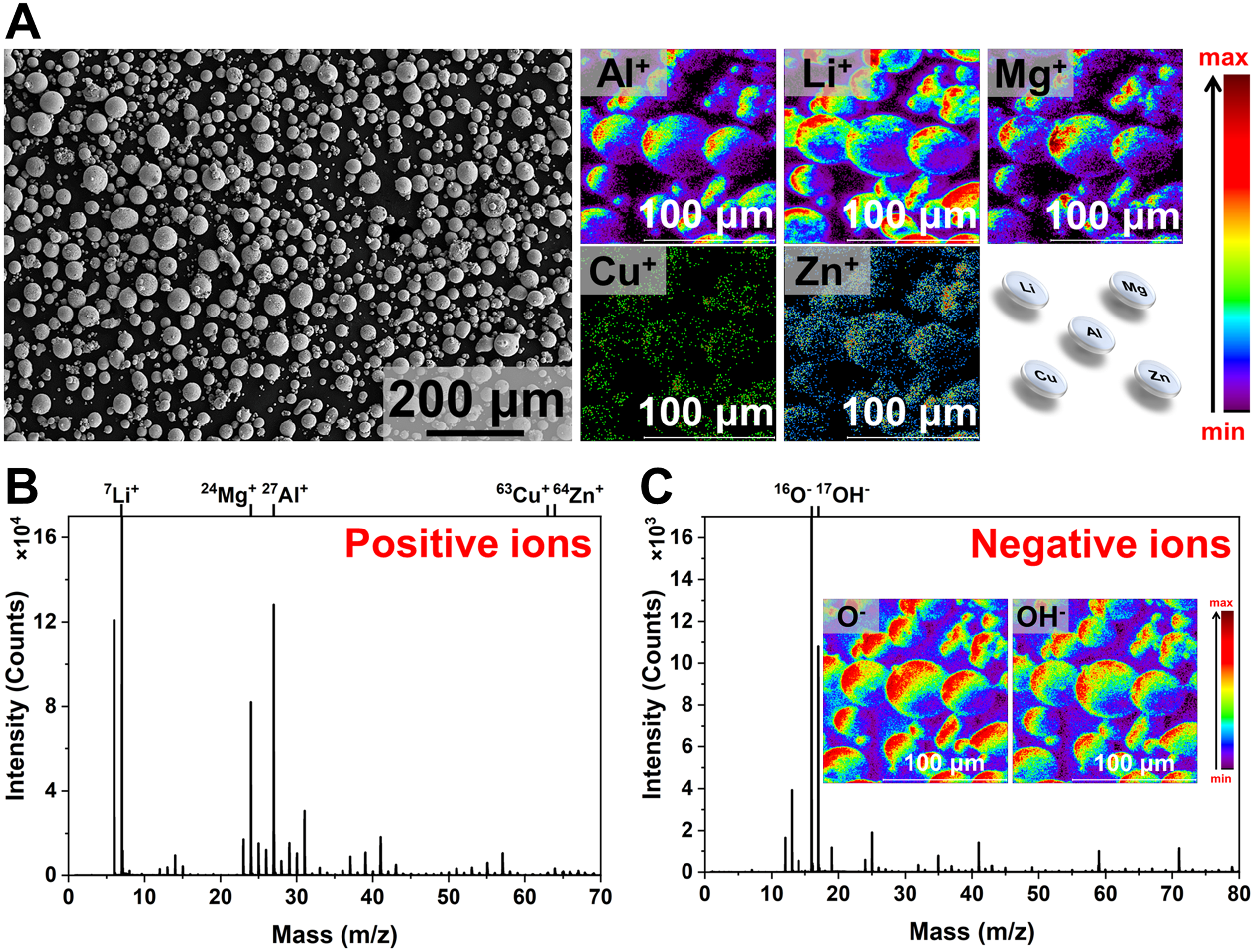


**Figure S6.** Powder morphology and TOF-SIMS ions distribution. A) SEM image of the powders, and TOF-SIMS positive ions distribution. TOF-SIMS mass spectra of B) positive ions and C) negative ions. The inserted images show the distribution of O^-^ and OH^-^.


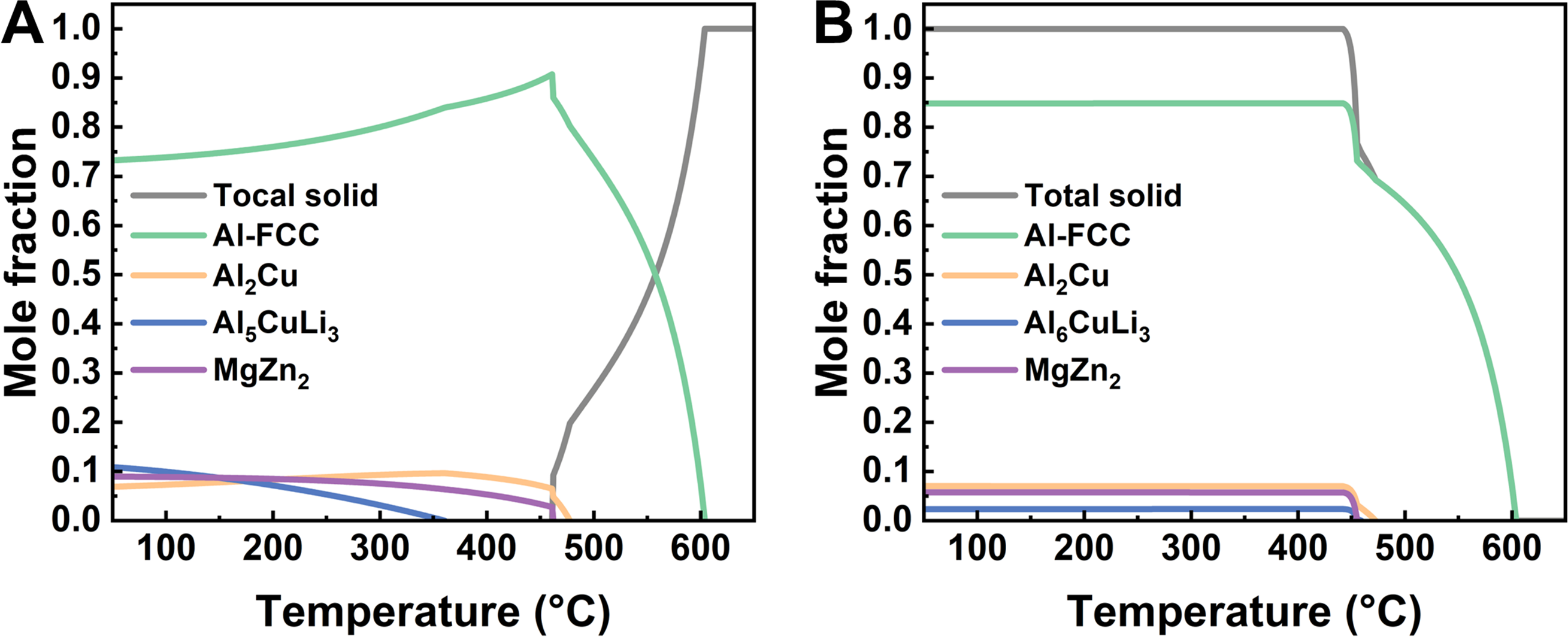


**Figure S7.** A) Equilibrium phase diagram of the Al_85_Cu_5_Li_4_Mg_3_Zn_3_ LAEA. B) Non-equilibrium phase diagram of the Al_85_Cu_5_Li_4_Mg_3_Zn_3_ LAEA during Scheil solidification.


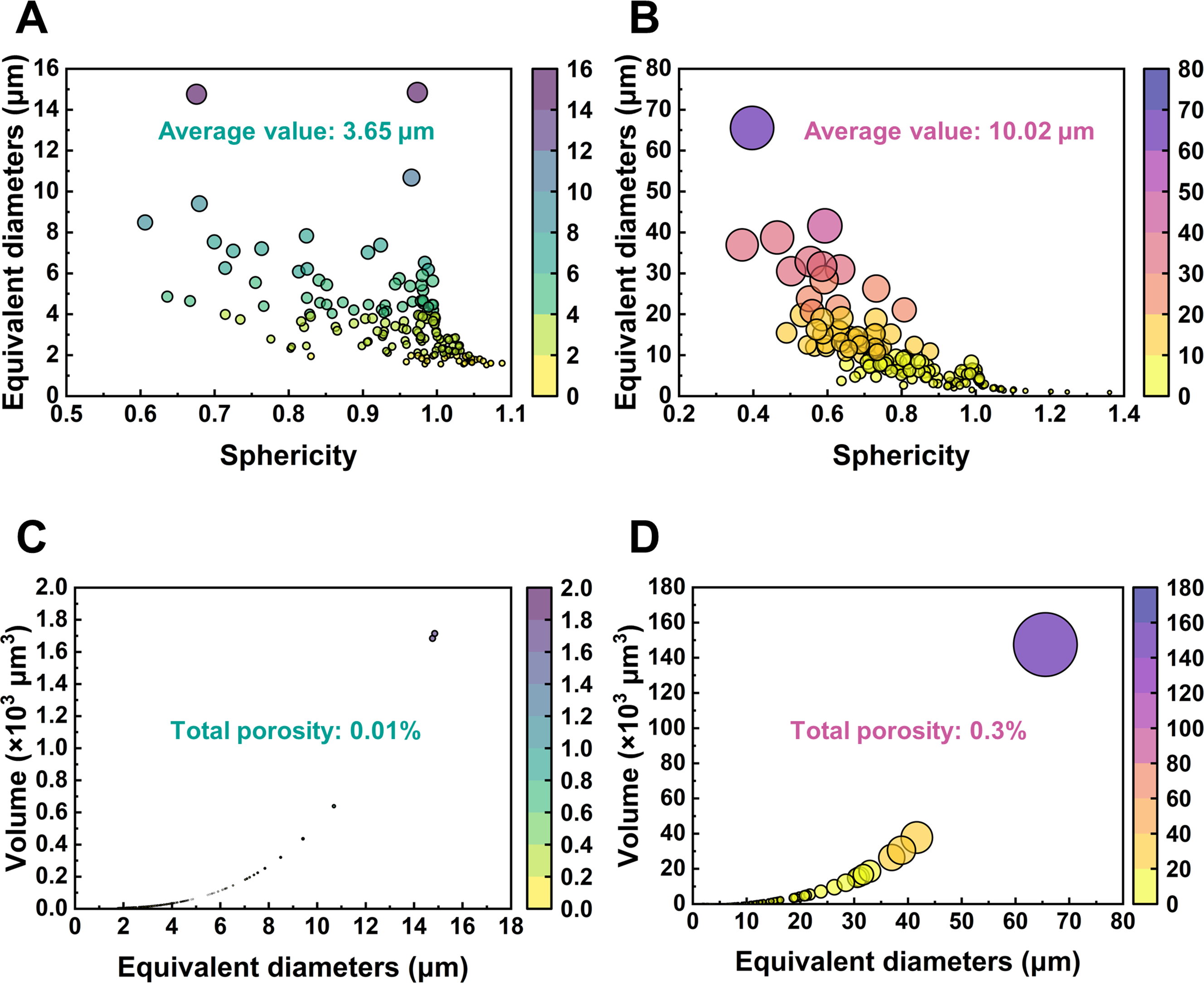


**Figure S8.** Quantitative volumetric analysis of the μ-CT data. A) and C) As-printed Al_85_Cu_5_Li_4_Mg_3_Zn_3_ LAEA. B) and D) As-printed Al-Cu-Mg alloy.


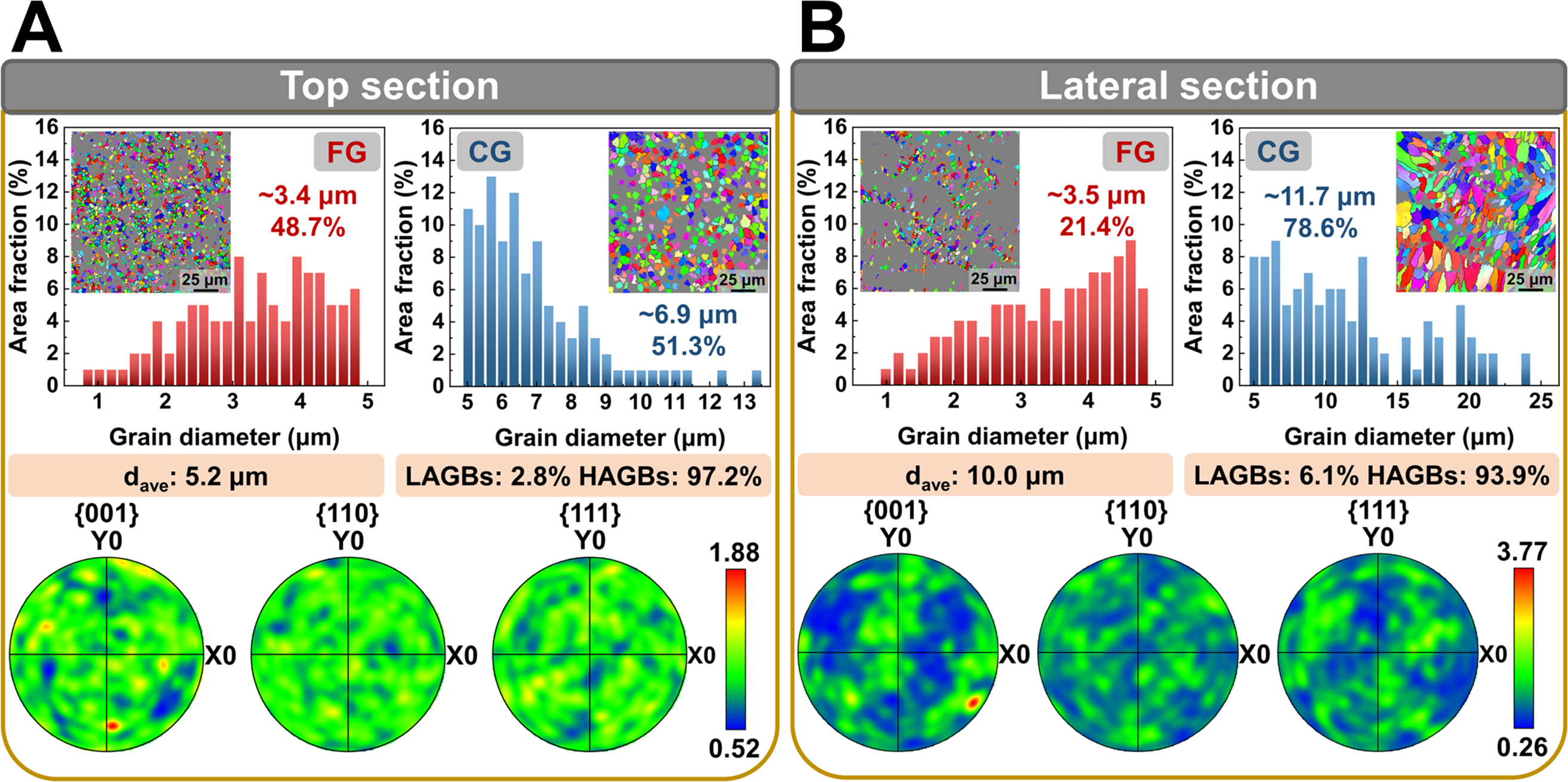


**Figure S9.** EBSD data for the A) top section and B) lateral section, including grain diameter distribution, grain boundary proportion, and polar figures.


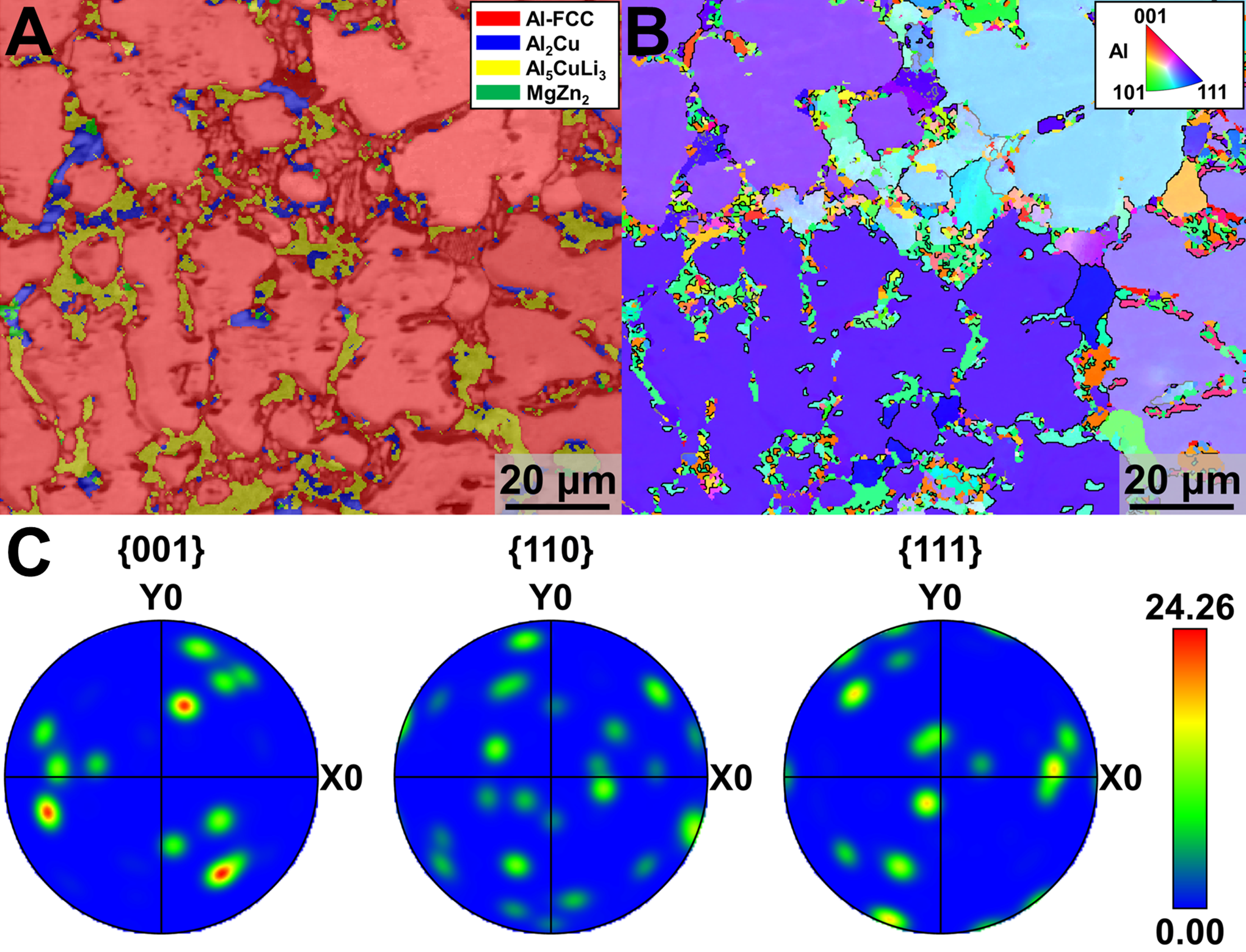


**Figure S10.** EBSD characterization of the as-cast Al_85_Cu_5_Li_4_Mg_3_Zn_3_ LAEA. A) Phase map. B) IPF map. C) Polar figures. The maximum MUD value of 24.26 reveals significant anisotropy.


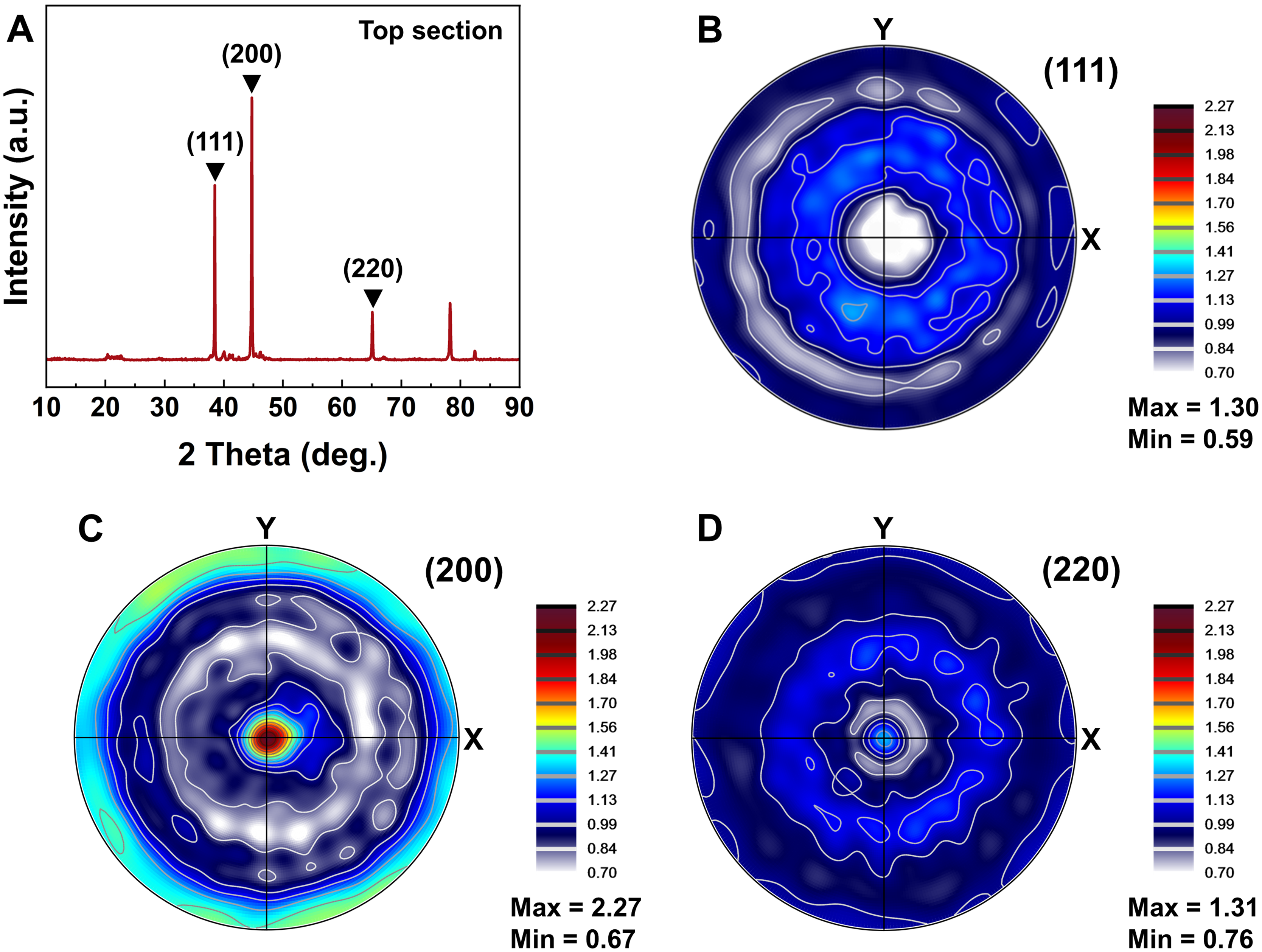


**Figure S11.** A) XRD pattern and pole figures of B) (111), C) (200), and D) (220) crystal planes on the top section of the as-printed alloy.


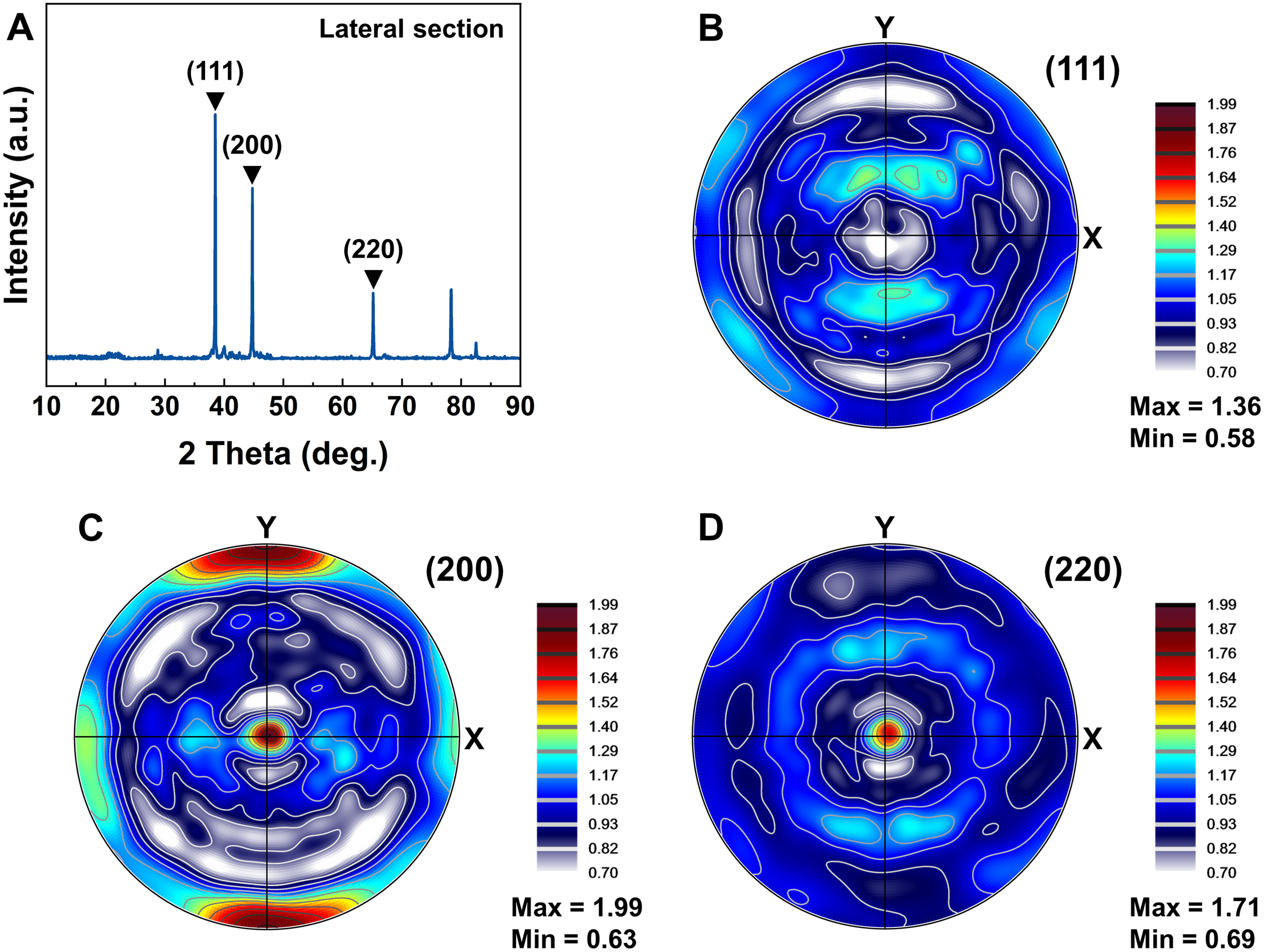


**Figure S12.** A) XRD pattern and pole figures of B) (111), C) (200), and D) (220) crystal planes on the lateral section of the as-printed alloy.


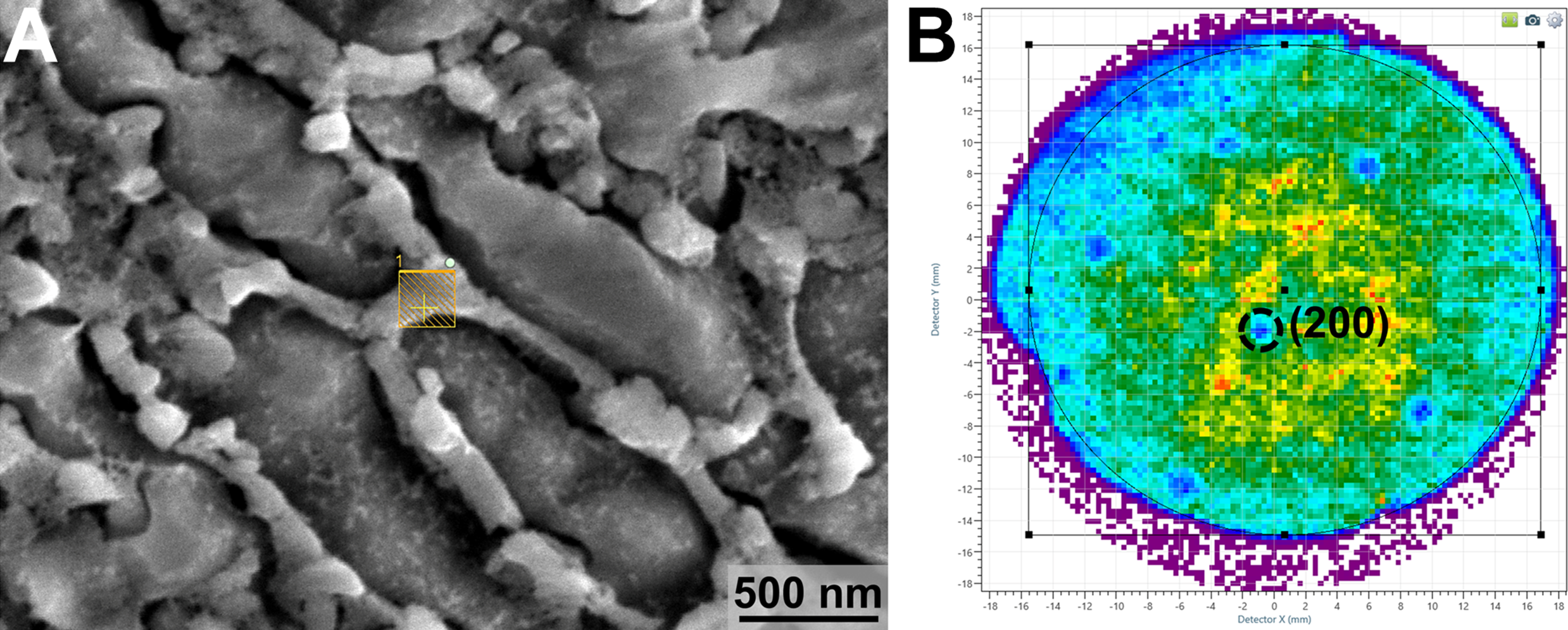


**Figure S13.** Cutting position and reconstruction of the APT tip. A) SEM image of the center showing the specific nano-sized eutectic network of the APT tip, extracted by FIB. B) 2D detector hit map with a (200) crystallographic pole labeled at the center.


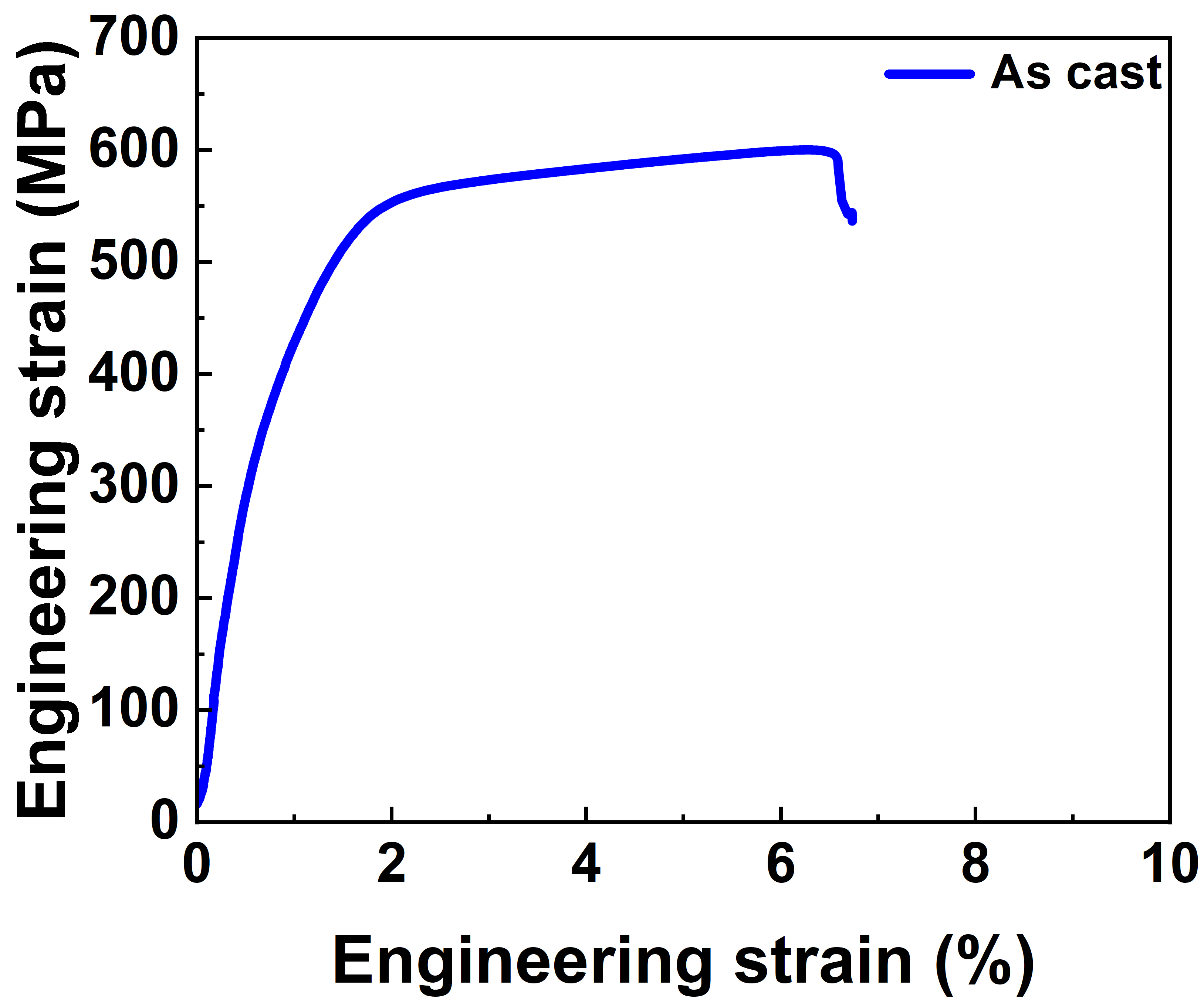


**Figure S14.** Compressive engineering stress-strain curve of as-cast Al_85_Cu_5_Li_4_Mg_3_Zn_3_ LAEA.


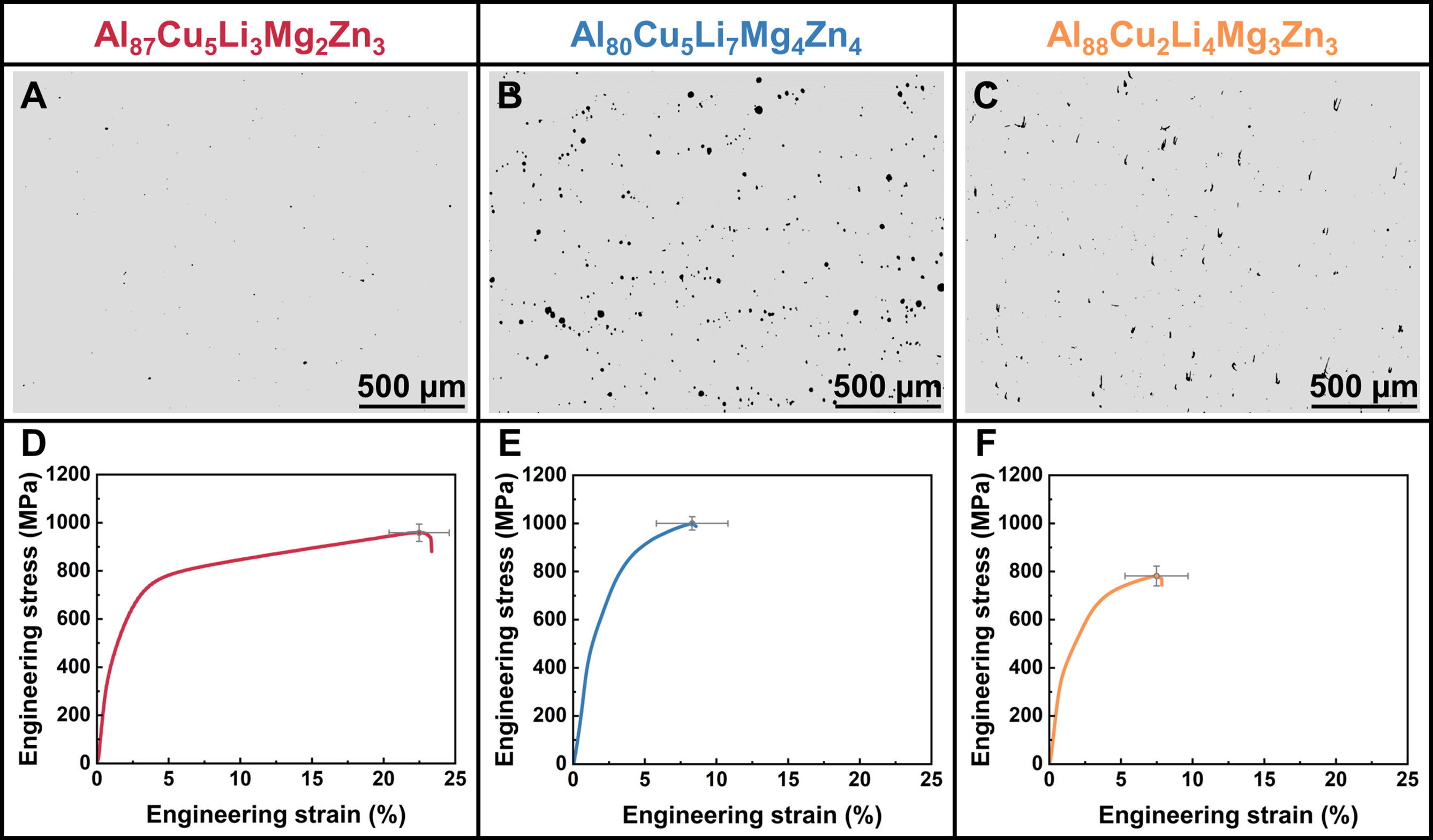


**Figure S15.** OM analysis of defects and compressive engineering stress-strain curves of candidate alloys, including positive verification and negative controls. A) and D) Al_87_Cu_5_Li_3_Mg_2_Zn_3_ LAEA. B) and E) Al_80_Cu_5_Li_7_Mg_4_Zn_4_ LAEA. C) and F) Al_88_Cu_2_Li_4_Mg_3_Zn_3_ LAEA.


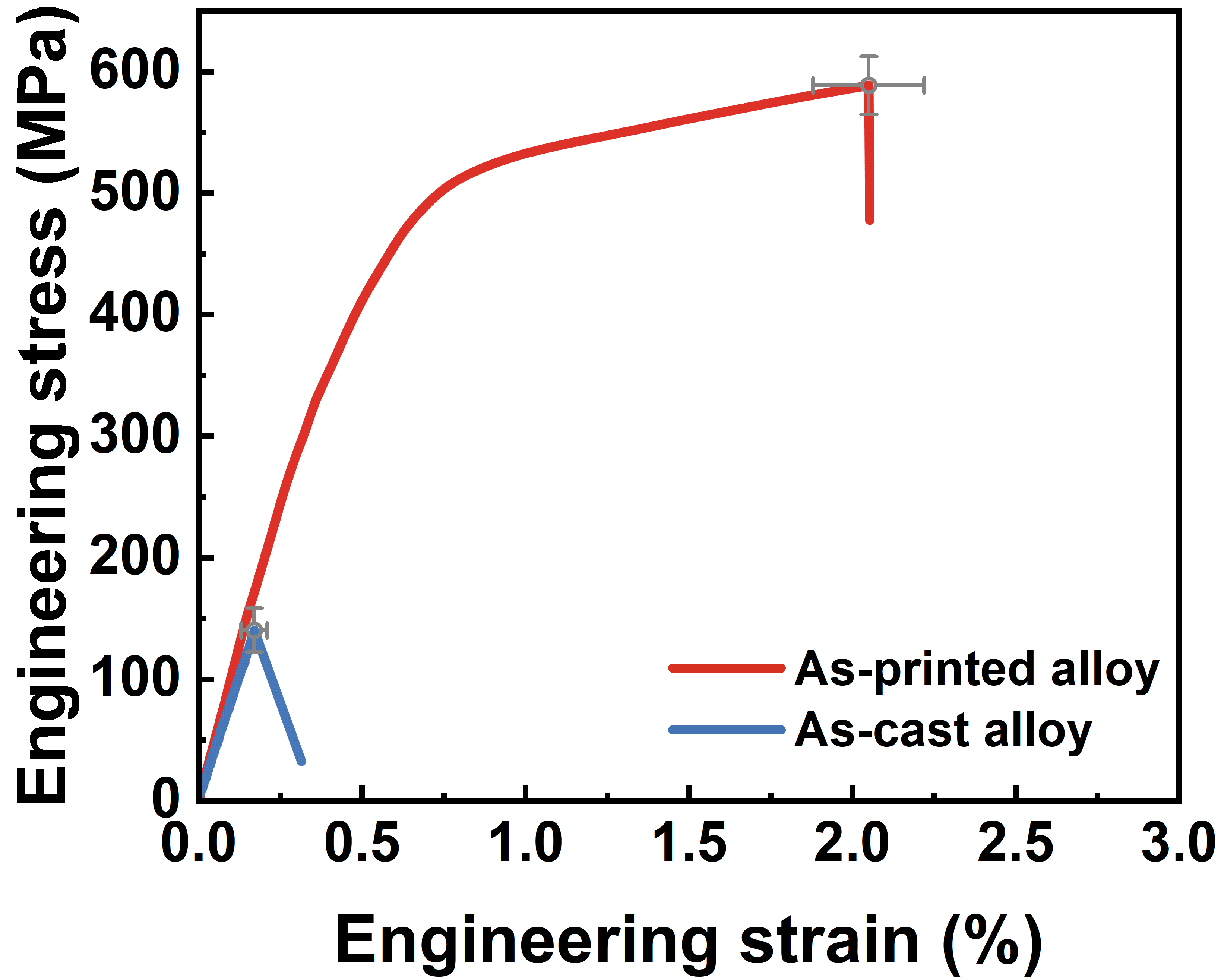


**Figure S16.** Tensile engineering stress-strain curves of as-cast and as-printed Al_85_Cu_5_Li_4_Mg_3_Zn_3_ LAEAs.


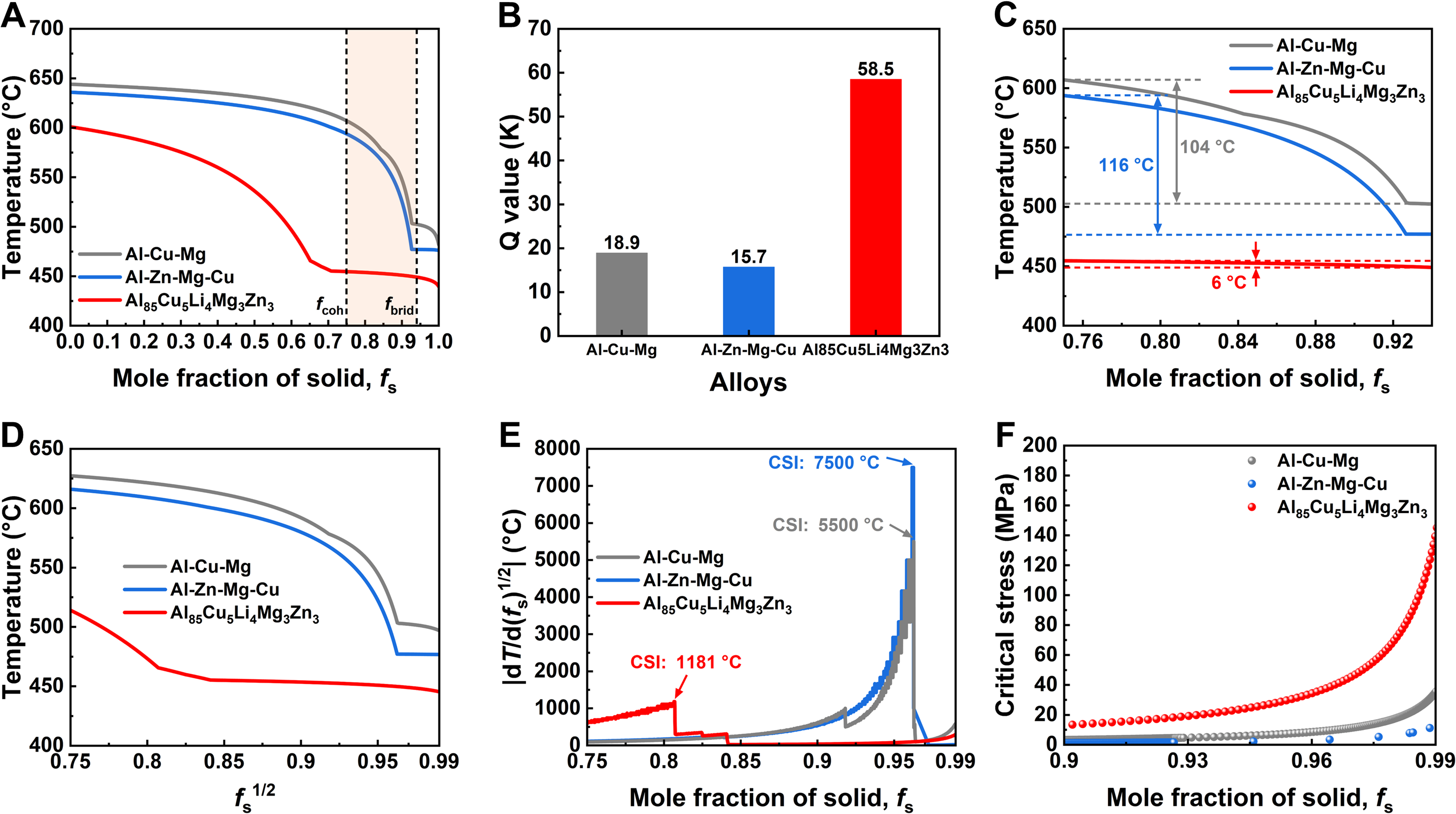


**Figure S17.** Calculation results of solidification based on the Scheil-Gulliver model for Al-Cu-Mg alloy,^[15]^ Al-Zn-Mg-Cu alloy,^[16]^ and Al_85_Cu_5_Li_4_Mg_3_Zn_3_ LAEA. A) Solidification paths. B) Q values. C) The enlarged solidification paths from *f*_coh_ to *f*_brid_ in (A). D) T-*f*^1/2^ curves. E) |d*T*/d(*f*_s_)^1/2^|-*f*^1/2^ curves showing CSI values. F) Critical stress versus *f*_s_ at the end of solidification.


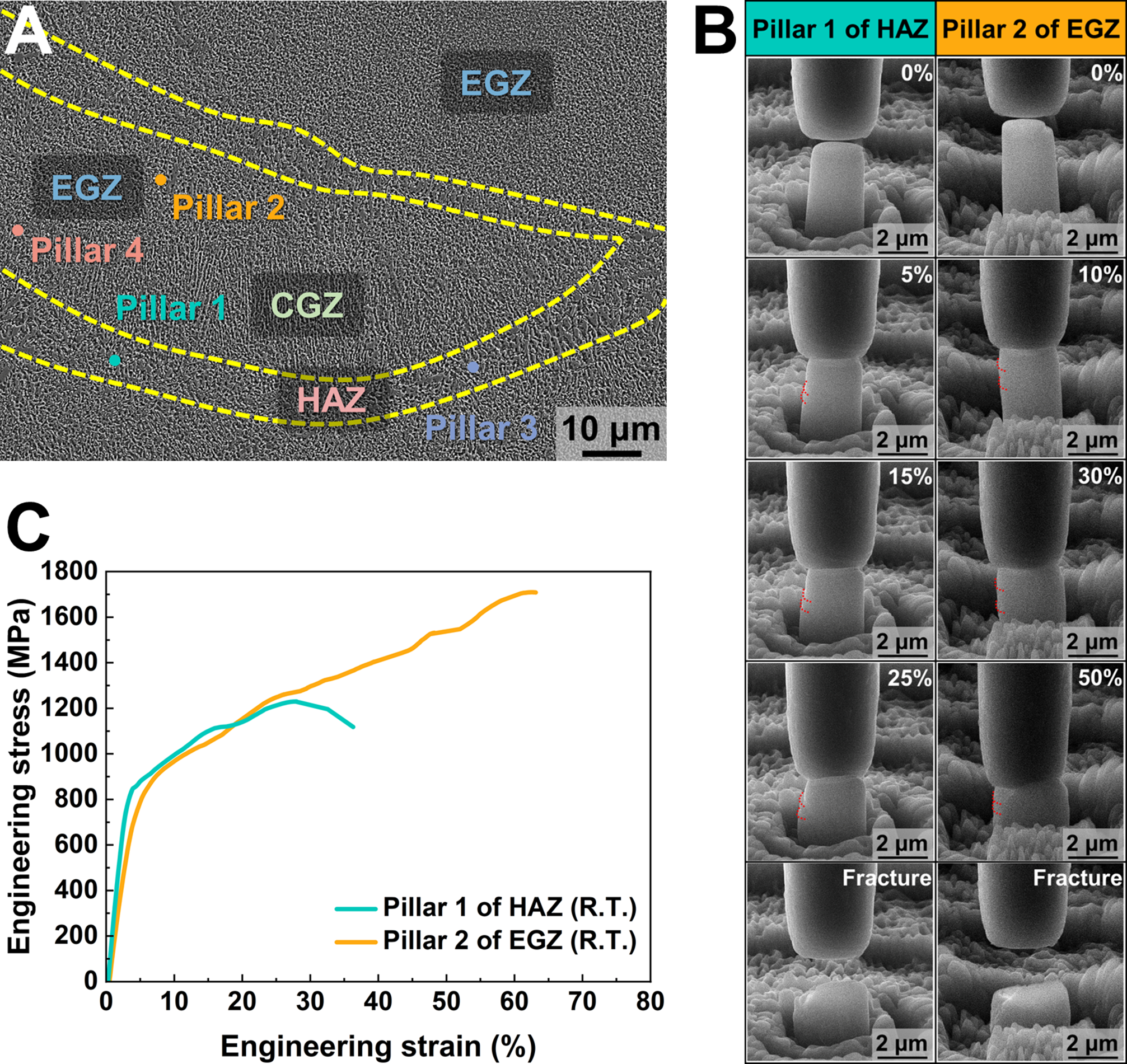


**Figure S18.** Micro-scale mechanical properties of as-printed Al_85_Cu_5_Li_4_Mg_3_Zn_3_ LAEA at room temperature. A) SEM image of the melting pool, containing EGZ, columnar grain zone (CGZ), and HAZ. The positions of micropillars are labeled. B) Morphological evolution of micropillars under compression, obtained from live video screenshots. The red dotted lines indicate the shear bands. C) Compressive engineering stress-strain curves of the micropillars, all with ultimate compressive strength exceeding 1 GPa and compressive strain exceeding 30%.


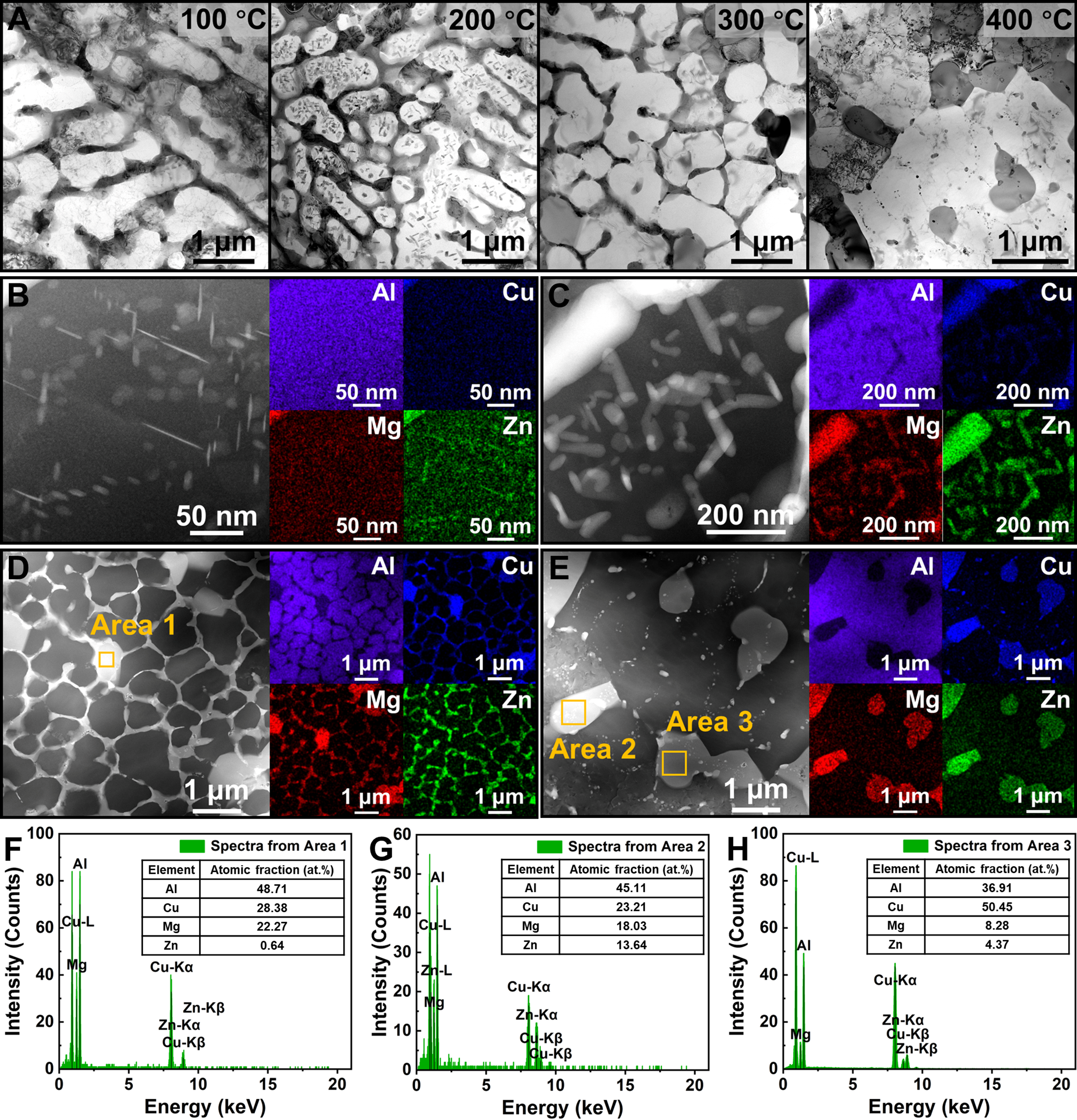


**Figure S19.** TEM morphologies and elemental diffusion of the as-printed Al_85_Cu_5_Li_4_Mg_3_Zn_3_ LAEA after thermal exposure. A) BF images of nano-sized cellular eutectic networks after thermal exposure at 100 ℃, 200 ℃, 300 ℃, and 400 ℃ for 100 h, respectively. B) HADDF-STEM image and EDS maps of precipitates in the matrix after thermal exposure at 100 ℃ for 100 h. C) HADDF-STEM image and EDS maps of precipitates in the matrix after thermal exposure at 200 ℃ for 100 h. D) HADDF-STEM image and EDS maps of eutectic phases after thermal exposure at 300 ℃ for 100 h. E) HADDF-STEM image and EDS maps of eutectic phases after thermal exposure at 400 ℃ for 100 h. F) to H) EDS-TEM spectrum of Areas in (D) and (E).


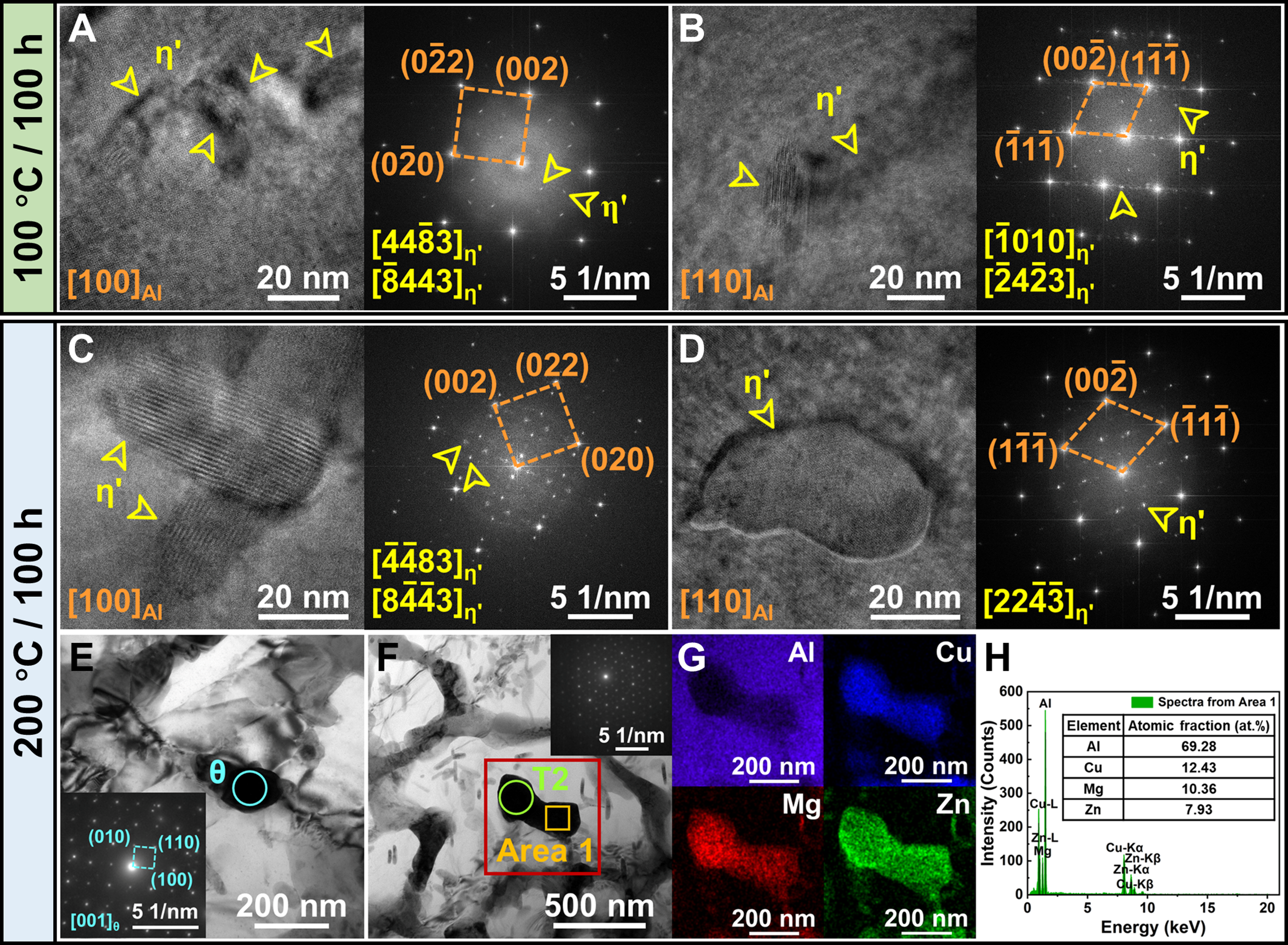


**Figure S20.** TEM morphologies of phases in the as-printed Al_85_Cu_5_Li_4_Mg_3_Zn_3_ LAEA after thermal exposure at 100 ℃ and 200 ℃. A) and B) HRTEM images and FFT patterns of η' phase after thermal exposure at 100 ℃ for 100 h. C) and D) HRTEM images and FFT patterns of η' phase after thermal exposure at 200 ℃ for 100 h. E) BF image of θ phase after thermal exposure at 200 ℃ for 100 h. The inset displays the FFT pattern. F) BF image of T2 phase after thermal exposure at 200 ℃ for 100 h. The inset displays the FFT pattern. G) EDS maps of red square in (F). H) EDS-TEM spectra of Area 1 in (F).


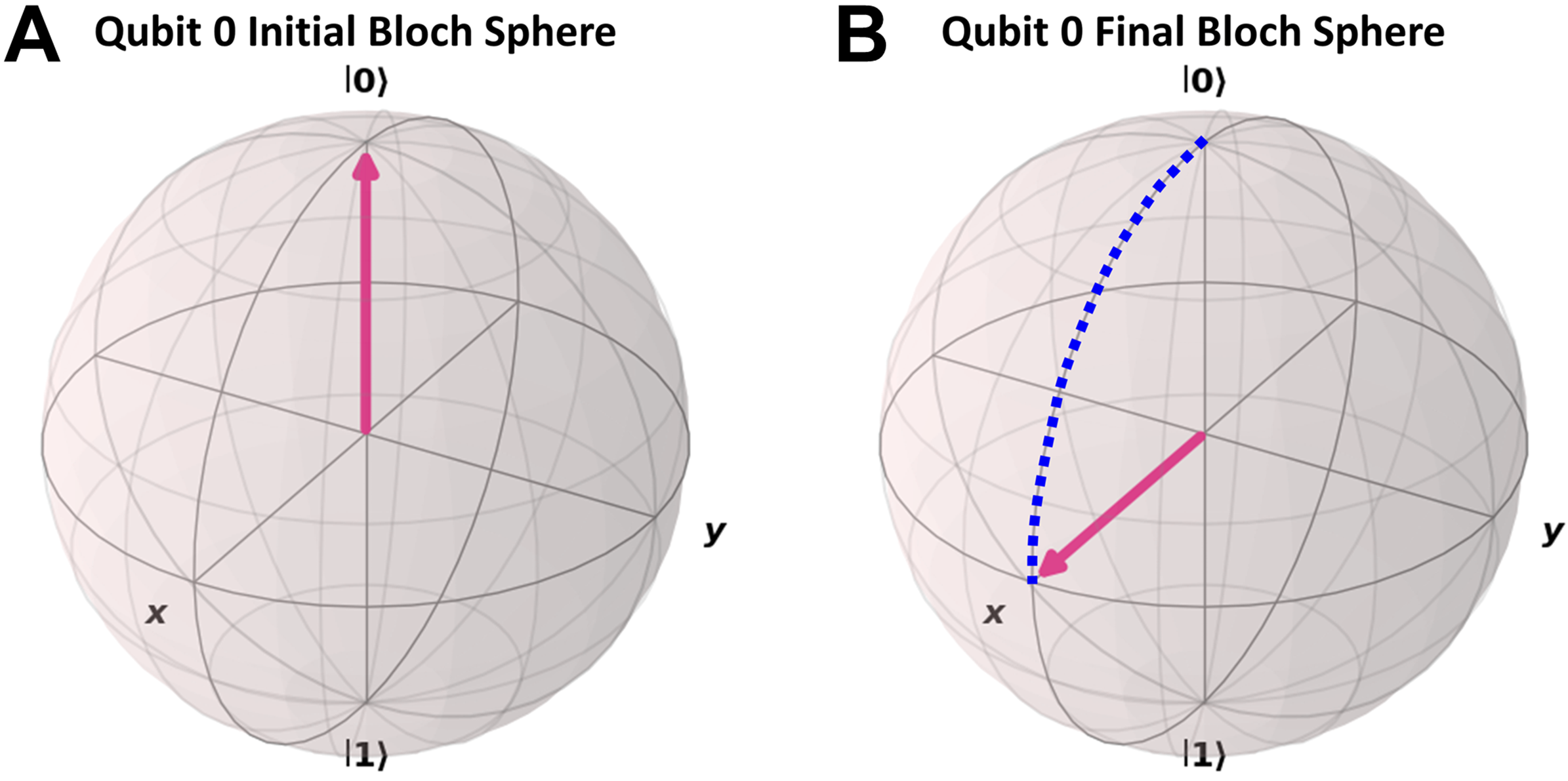


**Figure S21.** Example visualization of the rotation in the Bloch sphere of qubit 0. A) Initial state |0› of rotation indicated by red vector. B) Final state of rotation, indicated by the red vector, with the blue dashed line indicating the rotation angle between the final state and initial state.


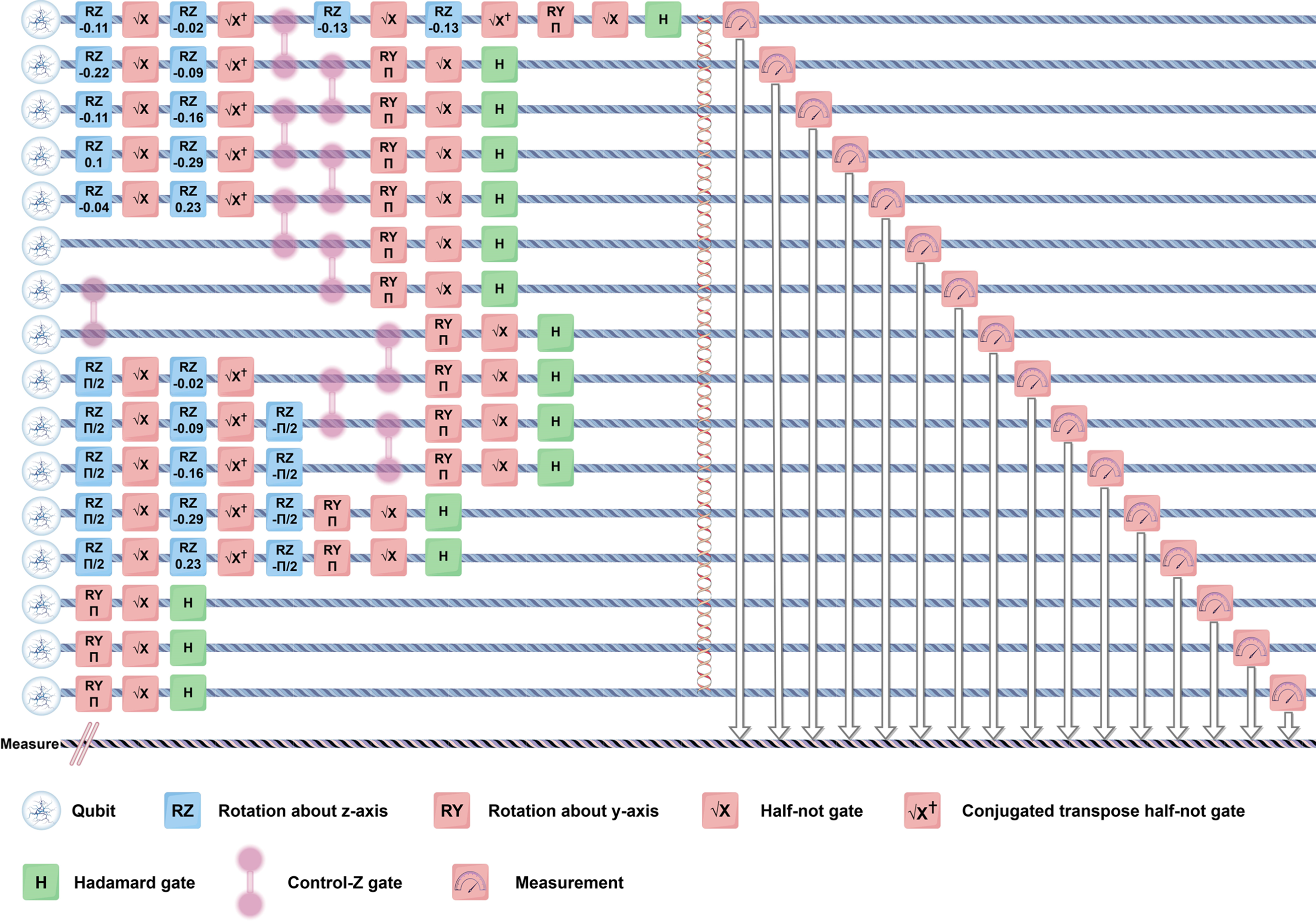


**Figure S22.** Quantum circuit diagram for feature selection and parameter optimization in Q-XGBoost model. The 16 qubits satisfy topological structure, and the circuit includes RZ, RY, √X, √X^┼^, H, Control-Z, and measurement gates.


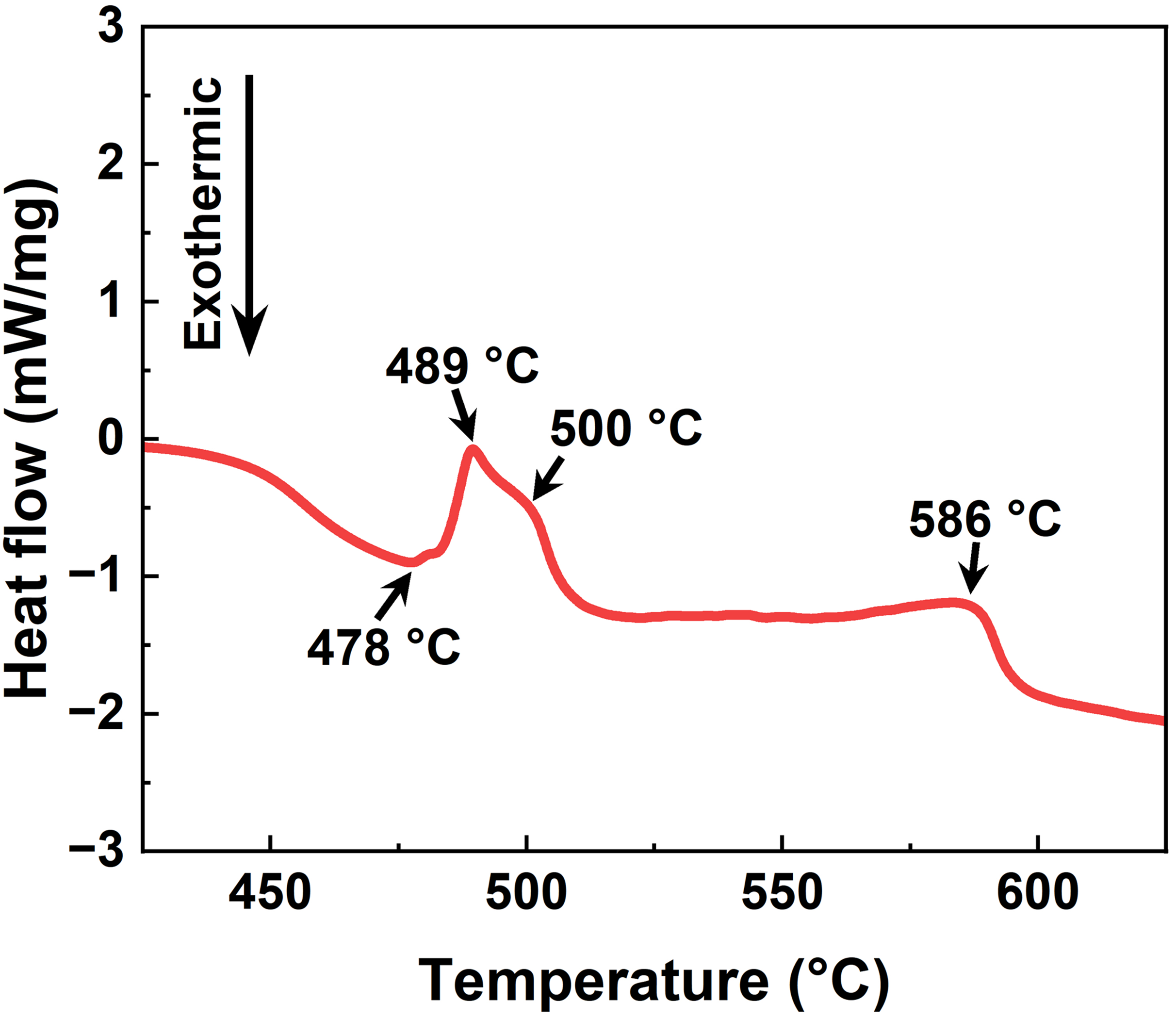


**Figure S23.** DSC curve of the as-printed Al_85_Cu_5_Li_4_Mg_3_Zn_3_ LAEA.

**Table S1.** Thermodynamic and mechanical parameters for simulation using the Solidification Cracking Model software.

| **Parameter** | **Symbol** | **Value** | **Unit** |
| --- | --- | --- | --- |
| Dimensionless back-diffusion parameter^[5]^ | *α* | 0.075 | - |
| Melting temperature^a)^ | *T*_m_ | 859 | K |
| Eutetic temperature^a)^ | *T*_E_ | 773 | K |
| Maximum solid solubility^[17]^ | *C*_SM_ | 5.65 | wt.% |
| Eutectic composition^[17]^ | *C*_E_ | 33.2 | wt.% |
| Coherent solid fraction^[5]^ | *f*_coh_ | 0.75 | - |
| Bridging solid fraction^[5]^ | *f*_brid_ | 0.94 | - |
| Critical stress^[5]^ | $\text{σ}_{\text{0}}^{\text{c}}$ | 10 | MPa |
| Temperature gradient^[18]^ | *G* | 10^6^ | K/m |
| Solidification velocity^[5,18]^ | *V* | 0.5 | m/s |

^a)^The value obtained from DSC curve (Figure S23).

**Table S2.** Thermo-physical parameters and material properties for simulation.

| **Parameter** | **Symbol** | **Value** | **Unit** |
| --- | --- | --- | --- |
| Liquidus temperature^a)^ | *T*_L_ | 859 | K |
| Solidus temperature^a)^ | *T*_S_ | 751 | K |
| Eutectic temperature^a)^ | *T*_E_ | 762, 773 | K |
| Density | *ρ* | 2.95 | g/cm^3^ |
| Pre-exponential coefficient (Li)^[19]^ | *D*_0_ | 1.49×10^-8^ | m^2^/s |
| Activation energy (Li)^[19]^ | *Q*_g_ | 95.3 | KJ/mol |
| Pre-exponential coefficient (Mg)^[20]^ | *D*_0_ | 9.9×10^-5^ | m^2^/s |
| Activation energy (Mg)^[20]^ | *Q*_g_ | 71.6 | KJ/mol |
| Pre-exponential coefficient (Cu)^[20]^ | *D*_0_ | 1.06×10^-7^ | m^2^/s |
| Activation energy (Cu)^[20]^ | *Q*_g_ | 24.0 | KJ/mol |
| Pre-exponential coefficient (Zn)^[20]^ | *D*_0_ | 5.12×10^-8^ | m^2^/s |
| Activation energy (Zn)^[20]^ | *Q*_g_ | 22.2 | KJ/mol |
| L/S interface energy^[21]^ | *σ*_p_ | 0.24 | J/m^2^ |
| Grain boundary energy^[14,21]^ | *σ*_g0_ | 0.24 | J/m^2^ |
| Anisotropy strength of surface energy^[22]^ | *ε*_4_ | 0.02 | - |
| L/S interface width^[23]^ | *l*_p_ | 4Δx (Δx = 0.01) | μm |
| Grain boundary width^[14,23]^ | *l*_g_ | 4Δx (Δx = 0.01) | μm |
| Bulk nucleation density^[24]^ | *n*_b_ | 10^5^ | mm^-3^ |
| Mean bulk nucleation undercooling^[24]^ | Δ*T*^b^_n_ | 5 | K |
| Standard deviation of bulk nucleation distribution^[24]^ | Δ*T*^b^_σ_ | 1 | - |
| Fusion boundary nucleation density^[24]^ | *n*_fb_ | 10^10^ | mm^-3^ |
| Mean fusion boundary nucleation undercooling^[24]^ | Δ*T*^fb^_n_ | 2 | K |
| Standard deviation of bulk nucleation distribution^[24]^ | Δ*T*^fb^_σ_ | 0.5 | - |
| Heat source power | *Q* | 220 | W |
| Scanning speed | *V* | 600 | mm/s |
| Scanning spacing | *s* | 100 | μm |
| Thermal conductivity^b)^ | *λ* | 120 | W/(m·K) |
| Specific heat capacity^b)^ | *c*_p_ | 837 | J/(kg·K) |
| Latent heat of fusion^b)^ | *L*_m_ | 383 | KJ/kg |

^a)^The value obtained from DSC curve (Figure S23).

^b)^The value calculated by JmatPro software.

**Table S3.** Actual composition of pre-alloyed powders and Al_85_Cu_5_Li_4_Mg_3_Zn_3_ LAEAs.

| **Element (wt.%)** | **Al** | **Cu** | **Li** | **Mg** | **Zn** |
| --- | --- | --- | --- | --- | --- |
| As-cast alloy | 78.53 | 11.05 | 0.98 | 2.63 | 6.81 |
| Powders | 78.22 | 10.85 | 1.04 | 2.81 | 7.28 |
| As-printed alloy | 78.76 | 10.97 | 0.95 | 2.58 | 6.74 |

**Movie S1.**

In situ SEM video of micropillar compression test of pillar in the equiaxed grain zone at 200 ℃.

**Movie S2.**

In situ SEM video of micropillar compression test of pillar in the heat-affected zone at 200 ℃.

**Data S1.**

The dataset of features of high entropy alloys used for training machine learning models.

**Data S2.**

The dataset of features of Al-Li-Mg-Zn-Cu series lightweight aluminum-based entropy alloys used for high-throughput computational screening.

**References**

[1] R. Xia, S. Kais, “Quantum Machine Learning for Electronic Structure Calculations,” Nature Communications 9 (2018): 4195.

[2] I. D. Lins, L. M. M. Araújo, C. B. S. Maior, P. M. da S. Ramos, M. J. das C. Moura, A. J. Ferreira-Martins, R. Chaves, A. Canabarro, “Quantum Machine Learning for Drowsiness Detection with EEG Signals,” Process Safety and Environmental Protection 186 (2024): 1197–1213.

[3] P. Brown, H. Zhuang, “Quantum Machine-Learning Phase Prediction of High-Entropy Alloys,” Materials Today 63 (2023): 18–31.

[4] A. Pérez-Salinas, A. Cervera-Lierta, E. Gil-Fuster, J. I. Latorre, “Data Re-Uploading for a Universal Quantum Classifier,” Quantum 4 (2020): 226.

[5] W. Liu, G. Li, J. Lu, “Modeling Solidification Cracking: A New Perspective on Solid Bridge Fracture,” Journal of the Mechanics and Physics of Solids 188 (2024): 105651.

[6] M. J. Chae, A. Sharma, M. C. Oh, B. Ahn, “Lightweight AlCuFeMnMgTi High Entropy Alloy with High Strength-to-Density Ratio Processed by Powder Metallurgy,” Metals and Materials International 27 (2021): 629–638.

[7] C. B. Alcock, V. P. Itkin, M. K. Horrigan, “Vapour Pressure Equations for the Metallic Elements: 298-2500k,” Canadian Metallurgical Quarterly 23 (1984): 309–313.

[8] E. Y. Shafirovich, U. I. Goldshleger, “The Superheat Phenomenon in the Combustion of Magnesium Particles,” Combustion and Flame 88 (1992): 425–432.

[9] R. Hołyst, M. Litniewski, D. Jakubczyk, “A Molecular Dynamics Test of the Hertz-Knudsen Equation for Evaporating Liquids,” Soft Matter 11 (2015): 7201–7206.

[10] M. Rappaz, P. H. Thévoz, “Solute Diffusion Model for Equiaxed Dendritic Growth: Analytical Solution,” Acta Metallurgica 35 (1987): 2929–2933.

[11] P. Thévoz, J. L. Desbiolles, M. Rappaz, “Modeling of Equiaxed Microstructure Formation in Casting,” Metallurgical Transactions A 20 (1989): 311–322.

[12] A. Zinoviev, O. Zinovieva, V. Ploshikhin, V. Romanova, R. Balokhonov, “Evolution of Grain Structure during Laser Additive Manufacturing. Simulation by a Cellular Automata Method,” Materials and Design 106 (2016): 321–329.

[13] O. Zinovieva, A. Zinoviev, V. Ploshikhin, “Three-Dimensional Modeling of the Microstructure Evolution during Metal Additive Manufacturing,” Computational Materials Science 141 (2018): 207–220.

[14] M. Yang, L. Wang, W. Yan, “Phase-Field Modeling of Grain Evolution in Additive Manufacturing with Addition of Reinforcing Particles,” Additive Manufacturing 47 (2021): 102286.

[15] Q. Tan, J. Zhang, Q. Sun, Z. Fan, G. Li, Y. Yin, Y. Liu, M. X. Zhang, “Inoculation Treatment of an Additively Manufactured 2024 Aluminium Alloy with Titanium Nanoparticles,” Acta Materialia 196 (2020): 1–16.

[16] G. Li, B. Tunca, S. Senol, M. Casata, Y. Wu, Z. Chen, K. Vanmeensel, “Revealing the Precipitation Behavior of Crack-Free TiB2/Al-Zn-Mg-Cu Composites Manufactured by Laser Powder Bed Fusion,” Additive Manufacturing 66 (2023): 103460.

[17] J. Liu, S. Kou, “Crack Susceptibility of Binary Aluminum Alloys during Solidification,” Acta Materialia 110 (2016): 84–94.

[18] M. Okugawa, Y. Ohigashi, Y. Furishiro, Y. Koizumi, T. Nakano, “Equiaxed Grain Formation by Intrinsic Heterogeneous Nucleation via Rapid Heating and Cooling in Additive Manufacturing of Aluminum-Silicon Hypoeutectic Alloy,” Journal of Alloys and Compounds 919 (2022): 165812.

[19] A. RAHO, M. KADI-HANIFI, “Determination du coefficient de diffusion du lithium dans un alliage Al-7,4%at.Li; Determination of the diffusion coefficient of lithium in an Al-7.4at.%Li alloy,” Annales de chimie (Paris. 1914) 29 (2004): 10–45.

[20] Y. Du, Y. A. Chang, B. Huang, W. Gong, Z. Jin, H. Xu, Z. Yuan, Y. Liu, Y. He, F. Y. Xie, “Diffusion Coefficients of Some Solutes in Fcc and Liquid Al: Critical Evaluation and Correlation,” Materials Science and Engineering: A 363 (2003): 140–151.

[21] C. Han, P. Jiang, S. Geng, S. Gao, G. Mi, C. Wang, “Multiphase-Field Simulation of Grain Coalescence Behavior and Its Effects on Solidification Cracking Susceptibility during Welding of Al-Cu Alloys,” Materials and Design 211 (2021): 110146.

[22] C. Han, P. Jiang, S. Geng, L. Guo, K. Liu, “Inhomogeneous Microstructure Distribution and Its Formation Mechanism in Deep Penetration Laser Welding of Medium-Thick Aluminum-Lithium Alloy Plates,” Optics and Laser Technology 167 (2023): 109783.

[23] P. I. O’Toole, M. J. Patel, C. Tang, D. Gunasegaram, A. B. Murphy, I. S. Cole, “Multiscale Simulation of Rapid Solidification of an Aluminium–Silicon Alloy under Additive Manufacturing Conditions,” Additive Manufacturing 48 (2021): 102353.

[24] M. S. Mohebbi, V. Ploshikhin, “Implementation of Nucleation in Cellular Automaton Simulation of Microstructural Evolution during Additive Manufacturing of Al Alloys,” Additive Manufacturing 36 (2020): 101726.
